# *COMPOSITUM 2*/ *WHEAT FRIZZY PANICLE* dosage dependently regulates inflorescence and root architecture in *Triticeae* cereals

**DOI:** 10.1111/tpj.71040

**Published:** 2026-07-23

**Authors:** Christian W. Hertig, Ravi Koppolu, Victor H. Rabesquine Nogueira, Cornelia Marthe, Nagaveni Budhagatapalli, Stefan Hiekel, Kavya Amte, Irene M. Fontana, Yongyu Huang, Astrid Junker, Amanda S. Câmara, Thorsten Schnurbusch, Jochen Kumlehn

**Affiliations:** ^1^ Leibniz Institute of Plant Genetics and Crop Plant Research (IPK) Gatersleben Germany; ^2^ Martin Luther University Halle‐Wittenberg, Faculty of Natural Sciences III Institute of Agricultural and Nutritional Sciences 06120 Halle Germany

**Keywords:** *Triticum aestivum* (wheat), *Hordeum vulgare* (barley), genome editing, spike development, grain yield, cereal inflorescence, root architecture, spike branching, yield components

## Abstract

The distinctive spike‐shaped inflorescence architecture in temperate cereals is governed by a complex interplay of genetic factors, including the orthologous *WHEAT FRIZZY PANICLE* (*WFZP*) and barley *COMPOSITUM 2* (*COM2*), whose products belong to the APETALA 2/ ETHYLENE RESPONSE FACTOR (AP2/ERF) family of transcription factors. However, the precise regulatory mechanism driving their function as well as the influence of allele dosage on spike formation and fertility remain to be elucidated specifically in wheat. A comprehensive analysis of the phenotype and gene function was conducted to unravel the genetic and molecular mechanisms underlying the peculiar *rattail 1* (*rtt1*) barley phenotype. In wheat, new *WFZP* alleles were generated, including knockouts and in‐frame mutations in all three homoeoalleles. Our genetic analysis revealed that the *rtt1* phenotype is caused by a conserved *COM2* mutation. The subsequent analysis of the transcriptome data elucidated the molecular mechanisms of *rtt1* for spikelet meristem identity, underscored by a predicted compromised DNA‐binding capacity of rtt1. The combination of our newly generated mutant alleles in wheat has revealed the intricate regulation of inflorescence, grain, and root development, which is controlled by WFZP. This provides evidence of the indispensable role of functional WFZP proteins, with a particular focus on the *WFZP‐B* allele, which has hitherto received limited attention. This study also demonstrates the allele‐specific impact of WFZP on root development, influencing branching and length. The findings of this study demonstrate that COM2 and WFZP exert dosage‐dependent regulatory influence on the inflorescence and root architecture in *Triticeae* cereals.

## Significance Statement

FRIZZY PANICLE has been described as a principal regulator of cereal inflorescence development; however, the precise regulatory mechanism, specifically, the role of the *WFZP*‐*B* allele in spike architectural regulation is unknown. This study employs a combination of advanced genomics, biotechnology, and computational methods to elucidate the uncharacterized role of WFZP and COM2 in fine‐tuning both spike and root architecture, thereby enabling the enhancement of the yield potential of temperate cereals.

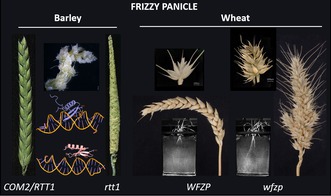

## INTRODUCTION

To meet the demands of a growing global human population and the necessity for more sustainable food production, it is imperative to enhance the yield of crops (Ray et al., 2013). The *Triticeae* cereals wheat (*Triticum aestivum* L.) and barley (*Hordeum vulgare* L.) are of particular agronomical importance and are among the most widely grown crops.

Historically, an increase in yield was achieved through a linear rise in the number of grains per square meter (Ferrante et al., 2017), while the number of spikelets, grain weight, and biomass remained almost unchanged (Sanchez‐Garcia et al., 2013). Similarly, the introduction of short‐straw varieties increased grain yield through a higher number of grains per ear without altering the number of spikelets (Youssefian et al., 1992a, 1992b). These prospects engage in a competitive dynamic for the scarce resources available. The number of spikelets per ear (Dobrovolskaya et al., 2015; Poursarebani et al., 2015), the number of florets and grains per spikelet, and grain size (Guo et al., 2017; Sakuma et al., 2019) are of particular relevance.

*WHEAT FRIZZY PANICLE* (*WFZP*) and barley *COMPOSITUM 2* (*COM2*) are orthologous members of the APETALA2/ETHYLENE‐RESPONSIVE FACTOR (AP2/ERF) transcription factor (TF) family. They were shown to play similar roles for the suppression of inflorescence branch outgrowth and specification of spikelet identity (Dobrovolskaya et al., 2015; Poursarebani et al., 2015). Moreover, *SHAM RAMIFICATION 2* (*SHR2*) was identified as a co‐acting locus for regulating inflorescence branch outgrowth (Dobrovolskaya et al., 2017). Additionally, Du et al. (2021) demonstrated that WFZP targets *BARREN STALK 1* (*BA1*), a bHLH transcription factor essential for lateral meristem initiation and spikelet formation. Importantly, previously identified orthologous mutant alleles of rice (*FZP*, Komatsu et al., 2003; and *BRANCHED FLORETLESS 1, BFL1*, Zhu et al., 2003), *Brachypodium distachyon* (*MORE SPIKELETS 1, MOS1*, Derbyshire & Byrne, 2013), and maize (*BRANCHED SILKLESS 1, BD1*, Chuck et al., 2002) were also shown to cause perturbed inflorescence branch outgrowth regulation, signifying the critical and conserved role of these genes in maintaining cereal inflorescence architecture.

Previous studies revealed the impact of conserved amino acid (AA) substitutions and knockout mutations in *COM2* and *WFZP* on inflorescence architecture (Dobrovolskaya et al., 2015, 2017; Du et al., 2021; Li, Li, et al., 2021; Poursarebani et al., 2015). Poursarebani et al. (2015) identified a branching pattern with reduced floret fertility in spikes of barley *com2.g* (Ser221Arg). In the tetraploid wheat *T. turgidum* convar. *compositum*, the A genome *WFZP*‐homoeolog harbors a conserved AA substitution (Leu96Pro) in “Miracle wheat” accessions (*bh* ^*t*^ *‐A1* allele) that exhibit strong spike branching as well as elevated grain number and overall yield potential (Poursarebani et al., 2015). The enhanced yield potential in “Miracle wheats” is primarily due to the replacement of spikelet meristems with “branch‐like” meristems during early spike development (Wolde & Schnurbusch, 2019). Du et al. (2021) revealed the perturbed interaction between BA1 and the mutated *WFZP‐A* allele Leu96Pro that was introgressed from tetraploid “Miracle wheat.” In diploid (*T. monococcum*; Cys70Tyr) and hexaploid wheat (*WFZP‐A*, *WFZP‐D*), multiple spontaneous and induced mutant alleles have been described (Dobrovolskaya et al., 2015, 2017; Du et al., 2021; Li, Li, et al., 2021) that showed the formation of supernumerary spikelets. However, genuine spike branching was not evident in the respective mutants. Moreover, it has been demonstrated that *wfzp‐A/D* double mutants exert a suppressive influence on root branching (Du et al., 2025), while the impact of single *WFZP* mutant alleles remains to be clarified. In addition, the influence of *WFZP‐B* allele has not been elucidated until now probably due to the integration of two transposable elements (TEs) that has been attributed to reduced expression in tetra‐ and hexaploid wheats (Du et al., 2021). Thus, the Du et al. (2021) study raises an intriguing question regarding the potential for a complete loss of WFZP or COM2 and its implications for plant growth, which has not been previously described and may suggest a critical need for functional WFZP/COM2 protein for spike development in wheat and barley.

RNA‐directed Cas9 endonuclease represents a valuable addition to the existing breeders' tool kit, as this technique allows for the directed modification of traits. Its potential for engineering barley and wheat has been demonstrated for numerous yield‐ or quality‐related traits (Budhagatapalli et al., 2020; Egorova et al., 2024; Gerasimova et al., 2020; Thiel et al., 2021; Wang, Pan, et al., 2018; Wang, Simmonds, et al., 2018). By employing genetic engineering along with manual crossing and haploid technology in wheat, we demonstrated that combinations of newly generated *WFZP* homoeoallelic variants resulted in distinct spike, grain, and also root phenotypes, depending on the degree of reduction in WFZP functionality. Along with wheat, we also investigated the genetics of the peculiar spike branching characteristic of barley *rattail 1* (*rtt1.a*), and revealed the allelism of *rtt1* with *COM2*. The *rtt1.a* allele is associated with a complete loss of fertility in developing spikes and can only be maintained in a heterozygous state (Robertson, 1967). Our transcriptome studies on *rtt1* in comparison with wild‐type barley elucidated the gene‐regulatory network of the *rtt1* mutant. Lastly, protein models of available and newly induced mutations were compared for functionally significant structural changes using AlphaFold's prediction of protein structure and of molecular dynamics simulations (MD). Thus, our study sheds light on some of the open questions concerning the function and significance of WFZP and COM2 protein in wheat and barley inflorescence development.

## RESULTS

### Barley *rattail 1* mutation leads to reversion of floret meristem to inflorescence meristem

**Figure 1:**
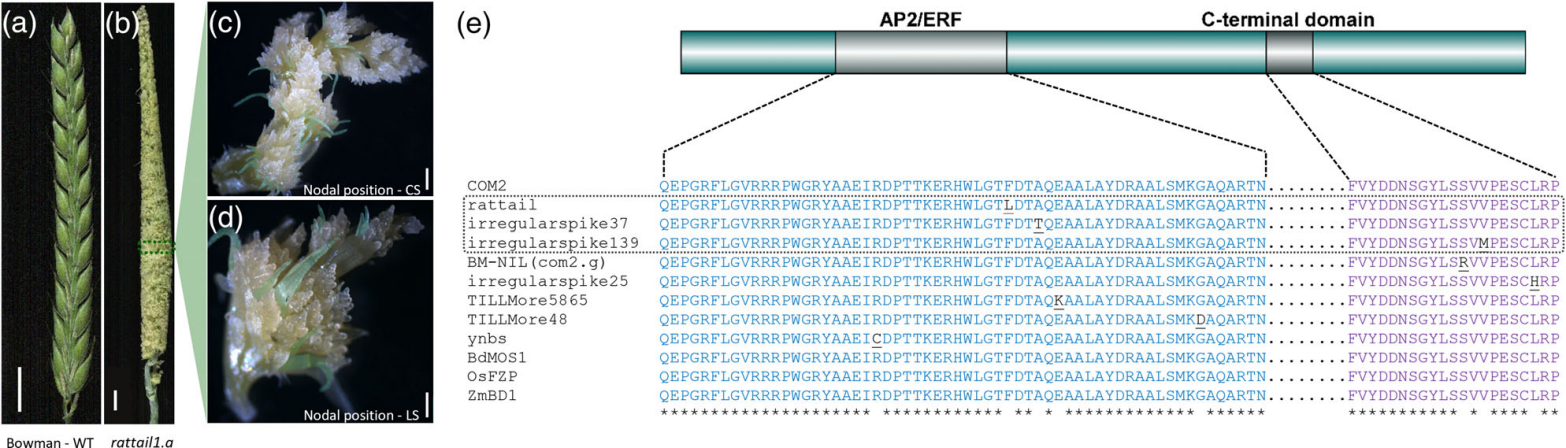
*rattail 1* spike phenotype and *COM2* mutations. (a) Wild‐type (wt) two‐rowed spike with normal spike development. (b) *rattail 1* spike with heavily branched inflorescence. (c, d) Nodal positions of central (c) and lateral spikelets (d) showing heavily proliferated inflorescence meristem‐like structures. (e) COM2 protein model localized with the newly identified mutant alleles *rattail 1* (AP2/ERF domain), *irregular spike 37* and *139* (C‐terminal domain). The remaining barley *COM2* alleles were reported in Poursarebani et al. (2015) or Zhou et al. (2023). CS—Central Spikelet, LS—Lateral Spikelet. White size bars represent 1 cm (a, b) and 500 μm (c, d).

The barley *rtt1.a* displayed a peculiar spike inflorescence type that resembles the tail of a rat, thus acquiring the appellation *rattail*. In contrast to less branched spike‐type inflorescences of barley, the *rtt1.a* mutant showed prolific branch outgrowth and complete spike sterility (Figure 1a–d). To untangle the phenotypic anomalies associated with *rtt1.a*, we undertook a pre‐anthesis developmental time‐course analysis of developing spikes that showed interesting differences between the wild‐type (*RTT1.b*) and mutant (*rtt1.a*). Initially, both inflorescences shared a similar developmental progression until the initiation of the glume primordium stage marking similar specification patterns of spikelet meristem identity (Figure S1). Followed by glume primordium stage, the *RTT1.b* inflorescence proceeded with the specification of floret meristem identity and floral organ differentiation (Figure S1). However, the developmental progression of *rtt1.a* inflorescence ceased at glume primordium stage, and an additional mound was initiated in the glume anlagen (Figure S1). Also, the presumptive floret meristem in *rtt1.a* attained inflorescence meristem‐like identity resulting in repeated proliferation of inflorescences in place of floret meristems, which led to overly branched inflorescences (Figure 1; Figure S1). This scenario was observed in both central and lateral spikelet meristems with varying branching intensities along the inflorescence axis (Figure 1c,d). From these developmental aberrations, it became apparent that the observed sterility in *rtt1.a* is indeed due to the lack of both specification of floret meristem identity and floral organ differentiation (Figure S1).

Since the developmental progression did not cross the glume primordium stage and inflorescence meristem‐like structures continuously emerged in place of floret meristems, *rtt1.a* spikes retained their juvenile state even at complete plant maturity (Figure 1b–d). Glumes were the lone differentiated spikelet organs observed in *rtt1.a*, consistent with the initiation of the glume primordium stage prior to the termination of floral organ differentiation (Figure 1c,d). Thus, our pre‐anthesis time‐course analysis revealed impaired specification of floret meristem identity and its reversion to a primitive inflorescence meristem‐like structure in the *rtt1.a* mutant.

### Genetic and genomic analyses reveal the causal gene for the *rtt1* phenotype

To understand the genetics underlying the *rtt1 phenotype*, we screened heterozygous germplasm obtained from the USDA, ARS. A total of 57 individuals were evaluated initially to phenotypically sort wild‐type and mutant (branched spike) types. Among these, 52 wild‐type and five mutant individuals were identified. Progeny of the wild‐type individuals (Table S1) were then evaluated to find those segregating for wild‐type and mutant phenotypes. This was the case in progeny from five individuals that were considered as genotypically heterozygous (*RTT1.b/rtt1.a*), and their progeny were treated as *rtt1* F_2_ populations for further genetic mapping (*rtt1* populations 1–5; Table S1).

Given the difficulty in mapping the *rtt1* locus owing to complete spike sterility in homozygous recessive state, we undertook a candidate gene approach for the identification of the gene underlying this locus. Previously, the *rtt1* locus had been genetically mapped to barley chromosome 2H (Robertson, 1967). Hence, we sequenced the genes located on chromosome 2H such as *COM2* (44.95 Mb), *SISTER OF RAMOSA 3* (*SRA3*, 87.23 Mb), *UNUSUAL FLORAL ORGANS‐LIKE* (*UFO*, 96.88 Mb), *FRUITFUL 3* (*FUL3*, 111.60 Mb), *FUL2* (381.29 Mb), *and LEAFY* (*LFY*, 618.06 Mb) that had previously been implicated in inflorescence developmental regulation in related species (Ikeda et al., 2007; Li, Debernardi, et al., 2021; Poursarebani et al., 2015; Satoh‐Nagasawa et al., 2006; Selva et al., 2021). Among the six sequenced putative candidate genes, only *COM2* showed a functional single‐nucleotide polymorphism (SNP) in *rtt1.a* plants (c.273C>A) that leads to a non‐synonymous AA substitution in the conserved AP2/ERF domain (Phe91Leu; Figure 1e). Single AA substitution analysis of Phe91Leu using the Plant Protein Variation Effect Detector pipeline (Gou et al., 2022) indicated that the substitution is deleterious (prediction score 0.998). Importantly, *COM2* had previously been implicated in spikelet meristem identity and as an inhibitor of branch outgrowth in barley and several other grass species (Derbyshire & Byrne, 2013; Huang et al., 2018; Poursarebani et al., 2015), reinforcing it as a strong candidate gene for the *rtt1* locus.

To further validate *COM2* as the *rtt1* candidate gene, we genotyped a *COM2*‐CAPS marker (c.273C>A, based on *rtt1.a* SNP; Table S2) in five *rtt1* F_2_ populations (*rtt1_pop 1–5*, Table S1; comprising 260 individuals). The marker data from all five populations showed 100% co‐segregation of the *COM2* marker with the *rtt1* spike phenotype (c.273C and c.273C/A—unbranched spike; c.273A—branched spike). Further evaluation of F_2/3_ progeny from eight heterozygous F_2_ families of *rtt1* population 1 (470 individuals) showed complete co‐segregation of *COM2* with the spike branching phenotype.

To rule out the possibility of large deletions, or far upstream/downstream variations within the *COM2* physical region as a cause of the *rtt1* phenotype, we conducted whole genome shotgun sequencing (WGS) of wild‐type and mutant DNA pools (≈20×). The resultant data confirmed the previously identified functional SNP in *rtt1.a* (c.273C>A). However, no other upstream/downstream sequence variations were evident within the *COM2* physical region (44.95 Mb; Figure S2). We also identified two other homozygous missense SNPs in a receptor‐like protein kinase (HORVU.MOREX.r2.2HG0102770; 95.88 Mb) and an AT hook motif‐containing protein (HORVU.MOREX.r2.2HG0159580; 614.04 Mb). However, these SNPs were located far away from the *COM2* physical region (Table S3), further reinforcing that the Phe91Leu substitution in COM2 is indeed responsible for the peculiar *rtt1.a*‐associated spike branching phenotype.

To further explore *com2* mutant alleles, we sequenced a collection of 15 barley *irregular spike* mutants (Nordgen, Sweden) that had been previously characterized for spike branching. Of these, two mutants harbored missense mutations in the conserved AP2/ERF domain or C‐terminal domain (*irregular spike 37*—c.280G>A; Ala94Thr; *irregular spike 139*—c.667G>A; Val223Met) (Figure 1e; Table S4). The branching intensity in the two new *irregular spike* mutants is comparable to those of previously identified *com2* alleles (Figure S3; Poursarebani et al., 2015) with proper floret differentiation/organ formation, thus making the *rtt1.a* allele a unique *COM2* functional mutation with impaired floret meristem identity/floral organ specification.

As the next validation step, heterozygous *RTT1.b*/*rtt1.a* individuals were crossed with those carrying the *com2.g* mutant allele. The F_1s_ were analyzed to reveal the zygosity of diagnostic *COM2* SNPs in *com2.g* (c.663C>A) and *rtt1.a* (c.273C>A) (Figure S4a,b). Of the 31 F_1s_ analyzed, 11 with heterozygosity at both SNPs displayed branched spikes, whereas 18 F_1s_ with homozygous wild‐type alleles at either *com2* or *rtt1* with heterozygosity at the respective locus displayed wild‐type unbranched spikes (Figure S4c). Of note, the double heterozygous F_1s_ (*RTT1.b/rtt1.a* + *COM2/com2*) displayed less severe branching compared to homozygous *rtt1.a* single mutants (Figure S4d). Such F_1_ plants often showed floral organ differentiation with developed central and lateral spikelets with occasional grain set (Figure S4e). The genetic and genomic analyses clearly established the mutation in *COM2* as being responsible for the excessive branch proliferation in the *rtt1.a* mutant.

### Transcriptome analysis reveals the gene regulatory network affected by the *rtt1* allele

**Figure 2:**
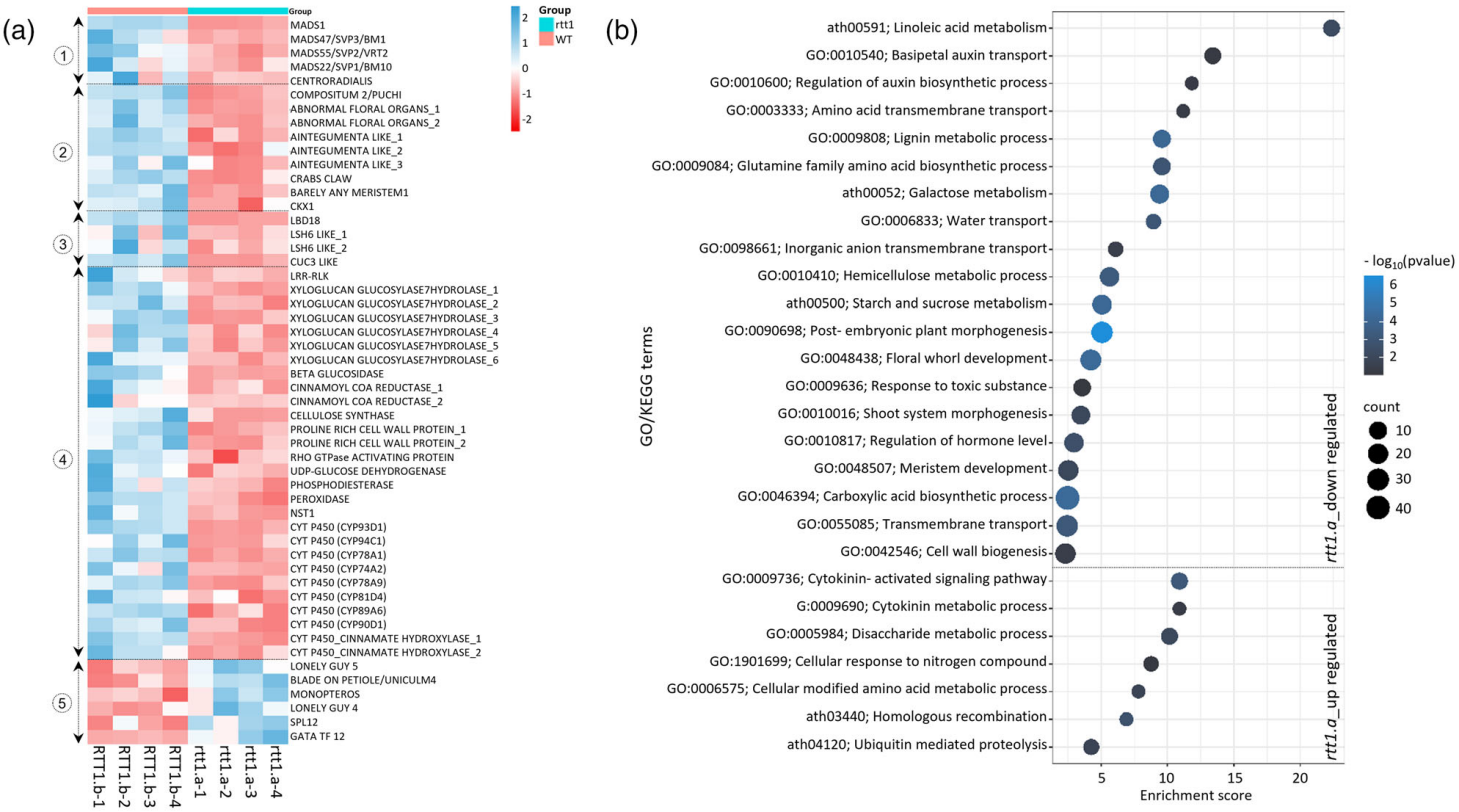
*rattail 1* differential expression analysis from RNA‐seq. (a) A subset of differentially expressed genes between *rtt1.a* and *RTT1.b* allele types. The full set of DEGs is available in Table S5. The down‐regulated genes in *rtt1.a* were partitioned into four groups based on functional relevance. Group 1 comprises genes relevant for maintenance of vegetative state, group 2 includes genes important for floral organ identity and floral determinacy. Group 3 represents genes functioning in organ boundary establishment, whereas that of group 4 belongs to genes important for cell wall patterning/modifications. Group 5 includes genes upregulated in *rtt1.a*. (b) Gene ontologies of *rtt1.a* down‐regulated and upregulated genes. The ontologies in both categories were sorted based on enrichment score. The complete set of ontologies are reported in Table S22.

To elucidate the molecular mechanisms underlying the regulation of spike branch outgrowth and deficiencies in floret meristem specification/floral organ differentiation, we performed RNA‐seq analysis from immature spike meristems of homozygous *RTT1.b* wild‐type at lemma primordium stage, while *rtt1.a* reproductive meristems were sampled 2–3 days post glume primordium initiation. From the analysis of wild‐type and mutant RNA‐seq data, we identified 838 differentially expressed genes (DEGs; 452 down‐regulated + 386 upregulated; Log2 FC ≥ 0.5; adjusted *P* < 0.05; Table S5) in *rtt1.a* mutants. Genes involved in delayed transition from vegetative to reproductive development, including three members of *SHORT VEGETATIVE PHASE* (*SVP*) gene family, *SVP1*, *VEGETATIVE TO REPRODUCTIVE TRANSITION* (*VRT2/SVP2*), *SVP3*, and *CENTRORADIALIS* were down‐regulated in *rtt1.a* plants (Figure 2a; Figure S5; Li, Debernardi, et al., 2021). Concurrently, the three *SQUAMOSA* genes *VERNALIZATION 1* (*VRN1*), *FUL2*, and *FUL3* important for timely reproductive transition were slightly upregulated in *rtt1.a* background (Figure S5; Li et al., 2019). Also, the *SEPALLATA* (*SEP*) class genes *HvMADS1* and *HvMADS5* essential for lemma identity specification and development (Shen et al., 2024; Zhang et al., 2024) were highly down‐regulated, which is consistent with the arrested specification of lemma identity in *rtt1.a* plants (Figures S1 and S5). Apart from these, several other genes important for proper floral organ initiation and development were down‐regulated, which is indicative of defective floral meristem identity in *rtt1.a* plants (Figure 2a,b).

Transcripts of *CUP SHAPED COTYLEDON 3*, *LIGHT DEPENDANT SHORT HYPOCOTYL 6‐LIKE*, and *LOB* domain genes important for organ boundary establishment proved to be down‐regulated, which is indicative of defective organ boundary establishment in *rtt1.a* mutants (Figure 2a). In addition, the cytokinin signaling pathway important for regulation of cell proliferation appears to be altered in the *rtt1.a* mutant. Transcripts of cytokinin biosynthetic genes (*LONELYGUY 4* and *5*) were upregulated, whereas that of cytokinin oxygenase/dehydrogenase (*CKX1*), a cytokinin degrading enzyme, was down‐regulated in *rtt1.a* mutants (Figure 2a,b; Chen, Jameson, et al., 2022). Gene ontology (GO) enrichment of genes down‐regulated in *rtt1.a* mutants also showed impaired auxin biosynthetic and transport processes, which corresponds to the floral developmental defects observed in these plants (Figure 2b).

Several genes important for maintaining cell wall properties and integrity were down‐regulated in the *rtt1.a* mutant, a phenomenon that is also evident in another barley spike branching mutant *com1* (Figure 2a,b). Several xyloglucan endotransglucosylase/hydrolases important for cell wall reconstruction processes showed reduced expression in *rtt1.a* mutants (Eklöf & Brumer, 2010). Also, the *CINNAMOYL CO‐A REDUCTASE* genes LRR‐RLK and CYP450s, which are important for lignin biosynthesis/cell wall lignin deposition, were down‐regulated (Barros et al., 2015; Gou et al., 2018; Van der Does et al., 2017). Apart from these, Rho GTPase‐activating protein and *NAC SECONDARY WALL THICKENING PROMOTING FACTOR 1‐LIKE* genes essential for cell wall patterning and cell wall thickening, respectively, were down‐regulated too (Mitsuda et al., 2005; Moon & Zheng, 2003).

We further carried out *in silico* analyses to interrogate whether the *RTT1* DEGs can be directly targeted by COM2 for their transcriptional regulation. Toward this end, we screened promoter/5′‐untranslated regions (UTRs) of *RTT1* DEGs (LOG2 FC ≥1.0; 221 down & 74 up) for scanning/enrichment of the COM2 consensus DNA‐binding motif (SCCGCC; Li, Li, et al., 2021) using targeted and untargeted analyses in the FIMO and EXTREME pipelines (MEME suite) (Tables S6 and S7). Both analyses revealed COM2‐binding motif enrichment in most of the DEGs (174 down & 60 up). The physical interaction between COM2 and the consensus DNA‐binding motif of its downstream targets had been validated in previous studies (Li, Li, et al., 2021; Lin et al., 2024; Wang et al., 2022), indicating a possible direct transcriptional control mediated by COM2 in the *RTT1*‐dependent DEGs identified. Thus, our RNA‐seq analysis revealed impaired transcriptional mechanisms important for timely vegetative‐to‐reproductive transition, as well as organ boundary and floret identity specification in *rtt1* plants. On top of that, our data showed that cell wall‐related signals may potentially impact the cell wall properties and signaling in the boundary regions to establish identity of the adjacent meristems, which is similar to the proposed function of COM1 (Poursarebani et al., 2020).

### Targeted mutagenesis of *COM2* ortholog *WFZP* in wheat results in novel allelic variants

**Figure 3:**
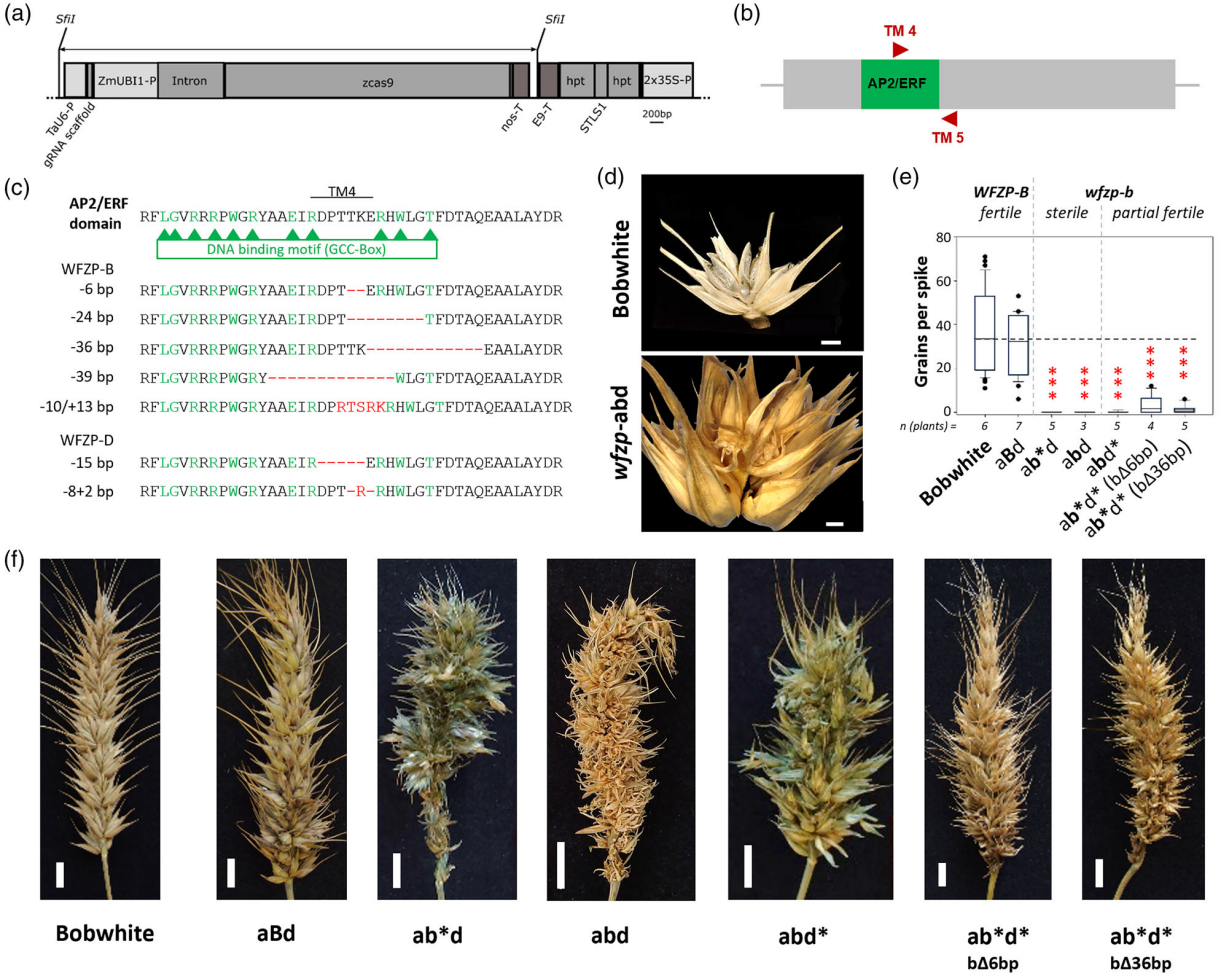
Modification of *WFZP* and its influence on fertility. (a) Schematic illustration of T‐DNA located on binary vectors pCH28 and pCH29; TaU6‐P: promotor of wheat *U6* gene; gRNA scaffold: unspecific 3′ part of gRNA (target‐specific nucleotides are 5′‐located); ZmUBI1‐P: promotor of maize *POLYUBIQUITIN 1* gene; Intron: 5′ UTR with first intron of maize *POLYUBIQUITIN 1* gene; zcas9: maize codon‐optimized *cas9* gene from *Streptococcus pyogenes*; nos‐T: terminator of *Agrobacterium tumefaciens nopalinsynthase* gene; E9‐T: terminator of pea *rbcS‐E9* gene; hpt: *E. coli hygromycin phosphotransferase* gene; STLS1: portable intron 2 (PIV2) of potato *ST‐LS1* gene; 2x35S‐P: doubled enhanced promotor of cauliflower mosaic virus *35S* gene; SfiI: cutting sites for SfiI restriction enzyme used for binary vector cloning. (b) Gene structure and location of selected target motifs (TMs) 4 and 5. (c) Amino acid sequence of AP2/ERF domain from in‐frame mutants; green: responsible amino acid residues for GCC‐Box DNA binding function based on NCBI conserved domain database (https://www.ncbi.nlm.nih.gov/cdd/; NCBI‐CDD‐ID: cd00018); red: mutations. (d) Spikelet of spring wheat breeding line Bobwhite wild‐type and *WFZP*‐*A/B/D* knockout (KO) mutant, white size bar represents 1 mm. (e) Grain number per spike from Bobwhite wild‐type compared to *WFZP*‐*A/D* and *WFZP*‐*A/B/D* mutants in dependence on the status of the *WFZP‐B* allele, *n* = 3–7 plants/variant, Kruskal‐Wallis test and Dunn's method were performed for statistical analysis, *P*‐values ≤0.001: ***. (f) Spike phenotypes of Bobwhite wild‐type and multiple *WFZP* mutants after growing in single pots, white size bar represents 1 cm; black asterisks represent in‐frame mutations.

In order to transfer the findings from barley COM2 to wheat and to determine the effects of reduced WFZP functionality by new allelic combinations, ballistic transformation of the wheat line “Bobwhite” was carried out using the binary gRNA/*cas9* vectors pCH28_WFZP_TM4 and pCH29_WFZP_TM5 (Figure 3a; Figure S6) addressing conserved target motifs (TM) located within (TM4) or downstream (TM5) of the AP2/ERF domain (Figure 3b). Off‐target analysis identified alternative target motifs with an incomplete NGG‐PAM or a maximum of 14 out of 20 target‐specific nucleobases, indicating that no mutagenic activity is to be expected (Table S8).

The co‐transfer of both binary vectors to 1200 isolated immature wheat embryos resulted in the formation of 128 plants, 12 of which exhibited integration of the *cas9*‐containing T‐DNA into the genome. Seven plants exhibited mutations in each of at least one target motif and one *WFZP* homoeoallele. Four of these plants were non‐transgenic and hence carried mutations introduced via transient *cas9* expression (Figure S7a). Only mutations induced in TM4 proved heritable.

To achieve higher diversity of mutated alleles and their combinations, the offspring of primary mutants were produced by self‐pollination and crosses in part followed by anther culture to generate doubled haploid (DH, entirely homozygous) segregants (Table S9; Figure S7c). The generation of genetically stable allele combinations for self‐pollinated and crossed plants usually requires up to five generations. By haploid technology, 82 progeny were produced from 11 primary mutants (Table S10), resulting in 32 fertile hexaploid regenerants and, ultimately, three DH plants carrying mutations in homozygous condition.

A total of 14 mutated and heritable *wzfp* alleles were generated comprising two, seven, and five residing in the A, B, and D subgenomes, respectively (Figure S8; Table S11). Of these, four B and two D alleles harbored in‐frame mutations that do not alter the translational reading frame for the WFZP protein but affected the AA composition of the GCC‐Box DNA‐binding motif (NCBI CDD‐ID cd00018) within the AP2 domain (Figure 3c; Table S12). The newly generated alleles occurred in plants carrying combinations of one, two, or three mutated *WFZP* homoeoalleles (Table 1). Furthermore, combinations of non‐inherited alleles that underwent mutations (Table S13) were deemed worthy due to the significant phenotypic changes observed. The prevalence of in‐frame mutations among the inherited alleles provides indications of the pivotal role of functional WFZP protein in relation to plant fertility.

**Table 1:** Generated, heritable *WFZP* alleles and their combinations

Allelic code	Plant‐IDs	*WFZP‐A*	*WFZP‐B*	*WFZP‐D*	T‐DNA
Bobwhite (wt)	E108	*wt*	*wt*	*wt*	
Plants with one mutated homoeoallele					
aBD	E111‐4‐2‐1‐1	Δ1 bp	*wt*	*wt*	
	C6/A‐1‐17‐1 C6/A‐1‐20‐5	Δ20 bp	*wt*	*wt*	
AbD	E101‐1‐1‐1‐2	*wt*	+1 bp	*wt*	
	E113‐3‐7	*wt*	Δ11 bp	*wt*	
Ab*D	97x113‐3‐1‐2‐3	*wt*	Δ10/+13 bp	*wt*	
	C6/A‐1‐15‐15	*wt*	Δ6 bp	*wt*	
ABd	97x113‐5‐3‐1‐1	*wt*	*wt*	Δ11/+1 bp	
	E111‐4‐8‐DH1	*wt*	*wt*	Δ18/+7 bp	
Plants with two mutated homoeoalleles					
abD	C4/A‐4‐9	Δ1 bp	+1 bp	*wt*	
ab*D	C6/A‐1‐15‐13	Δ20 bp	Δ6 bp	*wt*	
Abd	C3/A‐1‐2	*wt*	+1 bp	Δ11/+1 bp	
Ab*d	E113‐3‐1‐2‐2	*wt*	Δ39 bp	Δ1 bp	
aBd	C2/A‐3‐2‐8	Δ1 bp	*wt*	Δ11/+1 bp	
	E111‐4‐8‐DH3	Δ1 bp	*wt*	Δ18/+7 bp	
aBd*	C6/A‐1‐17‐16	Δ20 bp	*wt*	Δ15 bp	
Plants with three mutated homoeoalleles					
ab*d* bΔ6bp	E6‐3‐2‐2‐1	Δ20 bp	Δ6 bp	Δ15 bp	gRNA4, *cas9, hpt*
ab*d* bΔ36bp	E6‐3‐4‐5‐10	Δ20 bp	Δ36 bp	Δ15 bp	gRNA4, *cas9, hpt*
abd*	97x113‐4‐3‐DH8	Δ1 bp	Δ5/+31 bp	Δ8/+2 bp	gRNA4, *cas9, hpt*

Black asterisks and green font represent in‐frame mutations.

### Functional WFZP protein is necessary for grain formation

Observations of *WFZP* knockout phenotypes revealed a crucial role of functional WFZP protein. All generated plants featuring frameshift mutations in all three *WFZP* homoeoalleles (*abd*) exhibited many supernumerary spikelets and conspicuous spike branching but no grain production due to floret sterility (Figure 3d,e), as had also been described for barley carrying the *rtt1.a* allele in homozygous condition. A partially functional *WFZP‐B* allele carrying an in‐frame mutation (ab*d; with * indicating an in‐frame mutated allele) instead of frameshift was incapable of rescuing floret meristem development. The DH sister lines 97x113‐4‐3‐DH5 (*abd*) and 97x113‐4‐3‐DH8 (abd*) that just differ in their mutations within the D subgenome (frameshift vs. in‐frame) were phenotypically compared with each other. In contrast to DH5, grain formation was observed in DH8 plants most likely due to a partially functional WFZP‐D protein (Figure 3e,f; Tables S9 and S14). These findings illustrate the high relevance of functional WFZP protein being present and the specific role of *WFZP‐B* that carries a transposable element (TE) insertion in its promoter, which is thought to be the reason of lower gene expression (Dobrovolskaya et al., 2015; Du et al., 2021). It is noteworthy that the primary mutant E52 exhibited excessive spike branching along with spikelet and floret sterility (Figure S7b). It featured a chimeric state involving multiple alleles for all three *WFZP* subgenomic copies including wild‐type alleles (Figure S7a; Table S9). According to these observations, it is evident that a threshold dosage of functional WFZP protein is essential for the development of fertile florets and grain set.

### Mutated *WFZP* homoeoalleles affect wheat plant performance in various ways

**Figure 4:**
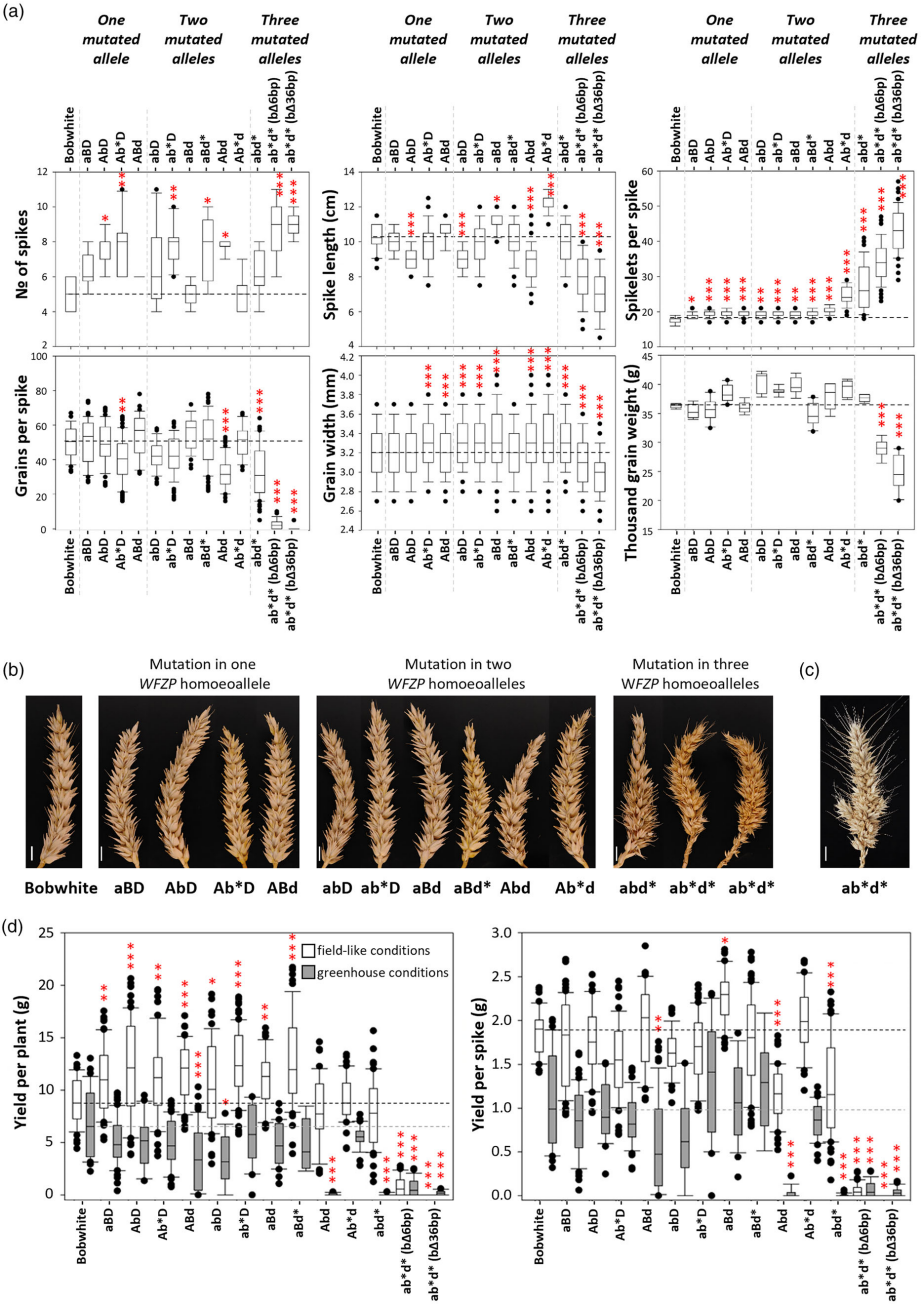
Phenotypic analyses of *WFZP* mutants in wheat. Plants growing in a plot under field‐like conditions with two rows of 8 plants of each mutant line surrounded by Bobwhite plants (a,b,d) or under standard greenhouse conditions in individual plant pots (c,d). (a) Investigation of plant architecture and yield‐related parameters under field‐like growing conditions, *n* = 10 plants/genotype. (b) Spike phenotypes of plants with one, two, or three mutated *WFZP* homoeoalleles under field‐like growing conditions. (c) Branched spike of triple mutated plant growing in single pot under standard greenhouse conditions. (d) Grain yield per plant and spike of *WFZP* mutants growing under field‐like conditions (white, *n* = 10 plants/genotype) or in single pots under standard greenhouse conditions (gray, *n* = 2–12 plants/genotype). Black asterisks represent in‐frame mutations (without shift of translational reading frame); black or gray dashed line represents median of Bobwhite wild‐type; red asterisks represent significant differences compared to Bobwhite wild‐type, ANOVA and Holm–Sidak method or Kruskal–Wallis test and Dunn's method were performed for statistical analysis, *P*‐values ≤0.05: *; ≤0.01: **, ≤0.001: ***; white size bar represents 1 cm.

To determine the different effects of mutated *WFZP* allele combinations, plants were grown in native soil in a small greenhouse, mimicking field‐like conditions. This environment resulted in distinctive patterns of plant development and performance that correlates with the allelic status of *WFZP*. Phenotypic investigations included plant and spike architecture traits (Figure 4a; Figure S9; Tables S15 and S16). Plants carrying frameshift (*aBD*, *AbD* or *ABd*) or in‐frame (Ab*D) mutations in one homoeoallele showed a moderate increase on spikelet numbers per spike and minimal influence on grain size. The examination of plants with two mutated homoeoalleles, either carrying frameshift (*abD*, *aBd*, or *Abd*) or a combination of frameshift and in‐frame mutations (ab*D, aBd*, Ab*d), revealed specific alterations on the whole‐plant architecture. Mutations in the *WFZP*‐*A* and ‐*B* subgenomic alleles (abD, ab*D) caused increased plant height and the production of a greater number of shorter spikes, producing moderately more spikelets but less grains. The grains were larger and had a higher thousand‐grain weight (TGW). Plants carrying *WFZP*‐*A/D* mutations (aBd, aBd*) displayed increased height, accompanied by a greater number of longer spikes that contain slightly more spikelets, potentially resulting in increased grain number. In comparison with wild‐type, the grains were observed to vary in width, either being wider (*aBd*) or narrower (aBd*), contingent upon the specific allelic combination present. There were no effects on plant height in plants carrying *WFZP*‐*B/D* (Abd, Ab*d) alleles. The combined knockout alleles of the *WFZP*‐*B* and ‐*D* copies resulted in reduced numbers of larger spikes with more spikelets; however, with a reduction in grain number. The mutation of all three *WFZP* homoeoalleles caused drastic changes in fertility. Plants with allele combination abd* produced slightly smaller spikes with more developed spikelets that contained less grains per spike due to higher floret sterility. The resulting grains were shorter while having more width. Compared with these plants, individuals carrying the allelic status ab*d* displayed reduced plant height and smaller spikes as well as significantly increased spikelet numbers per spike (Figure 4a,b). Here, the formation of grains was severely affected with smaller and lighter grains. In contrast to the cultivation as single plants in pots under standard greenhouse conditions (Figure 4c), spike branching was not observed under field‐like conditions (Figure 4b). These findings highlight the influence of WFZP on above‐ground plant tissue and the differential effects of combined mutated homoeoalleles for plant performance.

### Wheat grain yield potential depends on homoeoallelic combinations of *WFZP* mutants and their growth conditions

To better understand the effects of growth conditions, grain yield‐associated traits were evaluated in various *WFZP* allele combinations under standard greenhouse and field‐like conditions. The number of spikes, TGW, and grain number per spike were determined to derive grain yield per spike and plant (Figure 4d), respectively. Under field‐like conditions, the grain yield per plant increased in plants with single‐mutated *WFZP* alleles as well as combined mutations in *WFZP*‐*A/B*, or *WFZP*‐*A/D*, while the yields of *WFZP*‐*B/D* double mutants and all triple mutants were comparable to the Bobwhite wild‐type or reduced, respectively. At the whole‐plant level, the highest grain yield was observed in single B or D mutants (*AbD*, *ABd*), as well as in the combinations ab*D, aBd*. By contrast, at the spike level, the largest gain of yield was observed in plants harboring the dual frameshift mutation in the *WFZP*‐*A* and *‐D* alleles (*aBd*) (Table S15). In comparison, the plants cultivated under standard greenhouse conditions featured generally reduced yield due to reduced grain number per spike, except the double mutants ab*D, *aBd*, and aBd* that were comparable to the Bobwhite wild‐type (Table S17). This study reports the first detection of positive effects of a single *WFZP*‐*B* mutation and its combination with *WFZP*‐*A* mutation, respectively, on grain yield. In summary, the findings of this investigation demonstrate that the types and combinations of mutated *WFZP* alleles have a significant impact on yield‐related traits. This underscores the importance of a balanced regulation of WFZP for spike development and grain formation.

### WFZP regulates root branching

**Figure 5:**
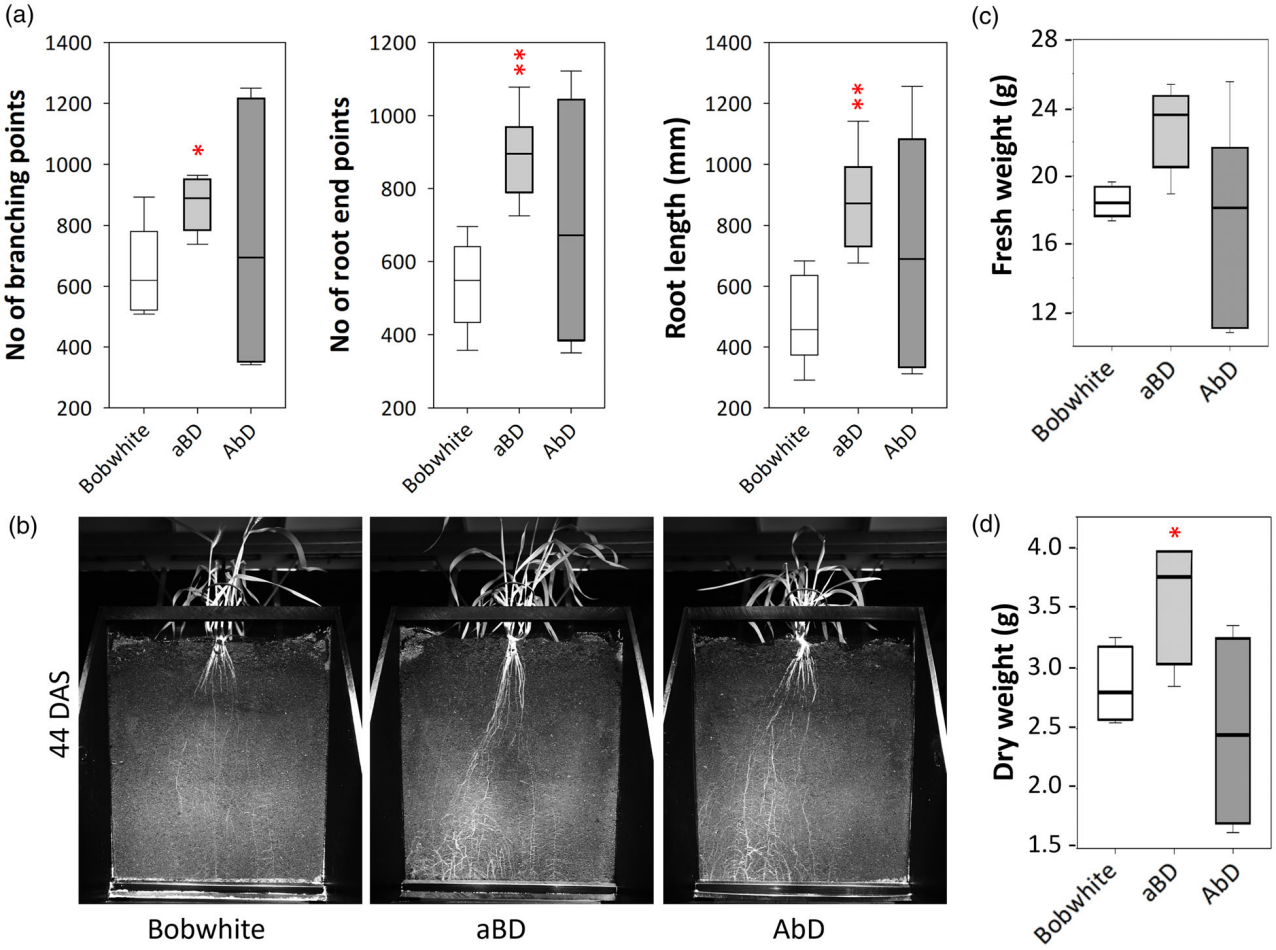
Root growth and shoot biomass of wheat plants carrying knockout mutations in *WFZP*‐*A* or ‐*B* homoeoalleles (aBD, AbD). (a) Automatically measured root phenotyping parameters for branching and root length at 44 days after sowing (DAS). (b) Documentation of root growth at 44 DAS in root phenotyping pots with long‐pass near‐infrared (NIR) filter. (c, d) Shoot biomass measurement of fresh (c) and dried (d) shoot material, performed after final plant documentation. Red asterisks represent significant differences compared to Bobwhite wild‐type, *n* = 7 plants/genotype, ANOVA and Holm–Sidak method or Kruskal–Wallis test and Dunn's method were performed for statistical analysis, *P*‐values ≤0.05: *; ≤0.01: **.

Similar to spikes, roots also function as sink‐related organs. An automated root phenotyping system was utilized to investigate the effects of *WFZP*‐*A* or ‐*B* knockout alleles (*aBD*, *AbD*) on root development. *WFZP*‐A mutants exhibited significantly longer and more branched roots, forming a higher number of root end points (Figure 5a,b and Figure S10a; Table S18) and higher shoot biomass (Figure 5c,d; Table S19) compared to the Bobwhite wild‐type and *WFZP*‐*B* knockouts. Both *WFZP*‐*A* and ‐*B* knockouts displayed diminished plant height and shoot surface area, with *WFZP*‐*B* knockouts showing a pronounced reduction (Figure S10b; Table S20). The observations presented here demonstrate new findings about the role of WFZP in root development. Furthermore, based on the observed higher grain yield potential of *WFZP* mutants, increased nutrient uptake and grain‐filling capacity can be hypothesized to be caused by the gain of root branching.

### Protein variants of *rtt1* and *WFZP* mutant alleles feature reduced DNA binding affinity

**Figure 6:**
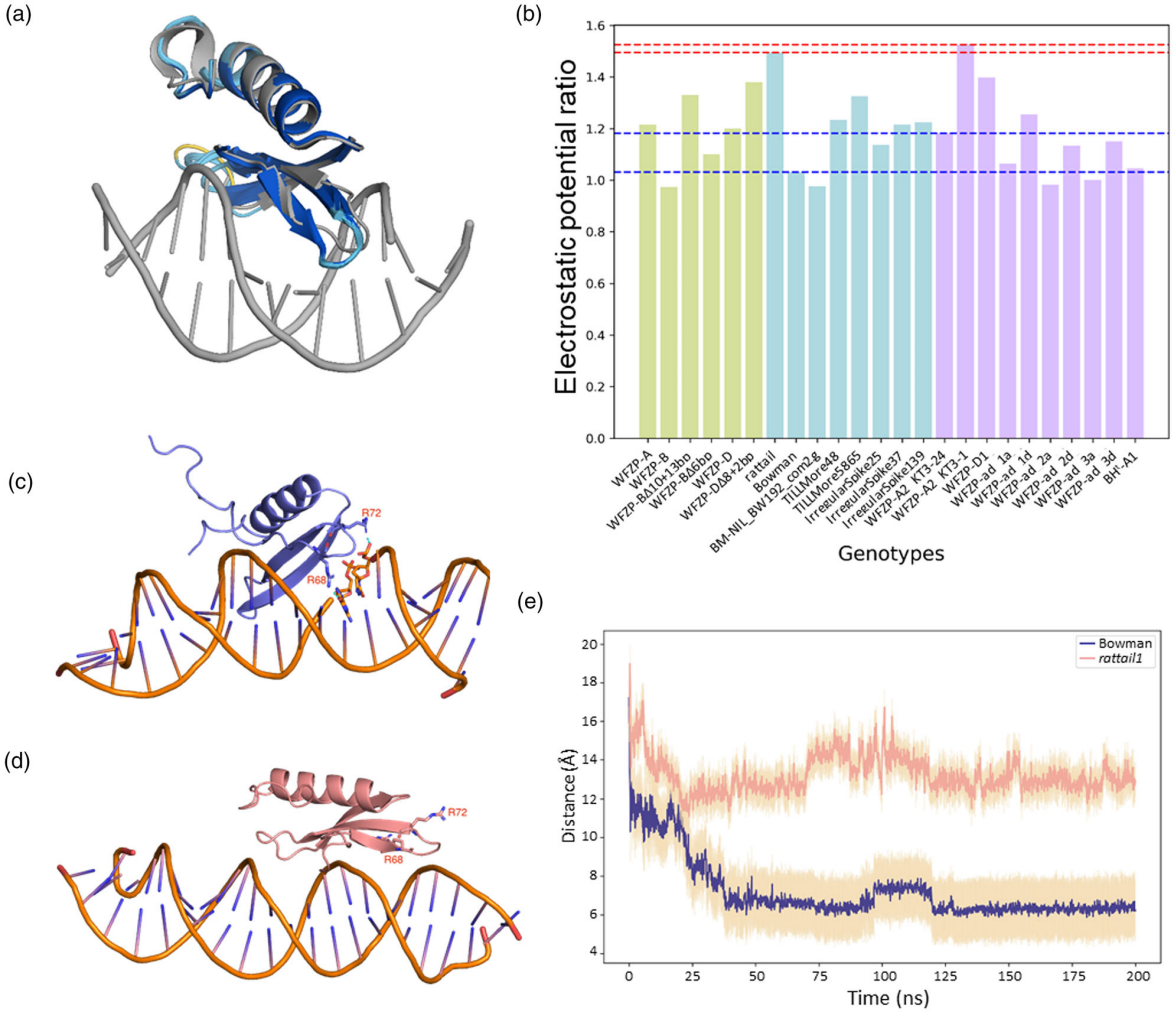
Protein structure analyses of *COM2* and *WFZP* mutants. (a) All predicted proteins with conserved AP2/ERF domain (colored according to AlphaFold's confidence) superposed with the AP2 domain of the flowering repressor TEM1 of *Arabidopsis thaliana* bound to DNA, solved by crystallography (gray, PDBID 7et4). (b) Electrostatic potential ratio between key arginines R68(65)/R72(69) for all genotypes whose predicted structure presented a complete AP2/ERF domain. Green bars indicate wheat *WFZP* alleles described in our study, blue bars indicate barley genotypes, and violet bar indicates wheat alleles from the literature. Red lines indicate most deleterious mutations of *rattail 1* and WFZP‐A2‐KT3‐1, and blue lines indicate the wild‐type Bowman and WFZP‐A2‐KT3‐24. Representative molecular dynamics simulation poses for (c) Bowman and (d) *rattail 1*, showing the positions of residues R68 and R72 with respect to the binding site. Hydrogen bonds (cyan) illustrate interactions within the complex. (e) Average distance between the Cζ atom of residue R68 and the binding site center of mass during molecular dynamics simulations for Bowman (blue) and *rattail 1* (coral).

We conducted an *in silico* protein structural analysis of WFZP/COM2 of wild‐types and mutants to predict the influence of conserved AA substitutions or in‐frame/frameshift mutations on protein functionality. To identify functionally significant structural changes, AlphaFold2 predictions were conducted based on four wild‐type (barley; wheat A, B, and D) and 21 mutant alleles (7× barley; 14× wheat; Table S21). Only the AP2/ERF domain was predicted to have an ordered globular structure in all analyzed proteins except frameshift mutations or deletions >6 bp. The predicted protein structure superposed well (RMSD = 0.45 Å) with the AP2/ERF domain from *Arabidopsis* solved by crystallography (Figure 6a) (PDBID 7et4). In a total of 11 predictions based upon *WFZP* alleles generated in the present study, the AP2/ERF domain was disrupted. With the exception of WFZP‐BΔ39bp, which lost the DNA‐binding interface, the disrupted domain left only a small stretch of the *β*‐sheet that directly interacted with the DNA in the solved complex of *Arabidopsis* and predicted complex models (Figure S11). Among the 10 protein predictions in this study described for barley and wheat mutants containing the complete AP2/ERF domain, only seven harbored a mutation within the domain, whereas the others had mutations in the C‐terminal region that were, however, predicted with very low confidence (Table S21). These seven mutations did not alter the secondary nor the tertiary structures of the AP2/ERF domain, which was confirmed in AlphaFold3 predictions with the DNA‐binding motif as exemplified for Bowman and the *rattail 1* mutant (Figure S12), showing no apparent reason for drastic functional roles.

Qualitative analyses of electrostatic surface distribution suggested changes at the DNA‐binding interface (Figure S13). Therefore, we developed an unconventional approach to the Adaptive Poisson‐Boltzmann Solver (APBS) calculation for electrostatic surfaces that usually delivers only visual inference. We retrieved the electrostatic potential per residue. The electrostatic potential of the arginines at Positions 68 and 72 changed most drastically for the barley rtt1 protein variant (Figure 6b) that is associated with the most pronounced spike branching phenotype. Furthermore, we predicted the structure and tested 12 previously reported WFZP mutants (Dobrovolskaya et al., 2015, 2017; Du et al., 2021; Poursarebani et al., 2015). We found a similar electrostatic shift in the wheat mutant for WFZP‐A2_KT3‐1 described as deleterious with Tyr70Cys mutation, neighboring the key arginines discovered in our analysis (Figure 6b; Table S21) (Li, Zhang, et al., 2022).

In our protein‐DNA complex models, Arg68 showed specific affinity toward DNA bases, while Arg72 interacted with the phosphate groups (Figure 6c). From this result, we hypothesized that a fine‐tuned distribution of charges between the two arginines is essential to coordinate the interaction with the DNA, directing one Arg to unspecific interactions with the phosphate groups and the other to specific interactions with the DNA bases. To test this hypothesis, we simulated the approach of the AP2/ERF domain of barley wild‐type (Bowman) and rattail 1 transcription factors to the DNA by molecular dynamics (MD) simulations. In the wild‐type, we observed that Arg68 consistently interacted with the DNA bases, while Arg72 interacted with the phosphates (Figure 6c–e and Figure S14; Movie S1), thereby adopting a similar conformation to the one predicted by AlphaFold3 (Figure S12). However, in the rtt1.a protein, challenging the prediction of AlphaFold3, Arg68 interacted only transiently with the DNA bases and remained mostly pointing away from the DNA (Movie S2). From this, we concluded that the rtt1.a‐specific Phe91Leu substitution, sitting opposite to two key arginines in the *β*‐sheet of the protein, possibly rearranges the surface charges in this locale and hinders the correct binding with the DNA.

## DISCUSSION

### Spikelet fertility in barley and wheat inflorescences is dependent on a fine‐tuned regulation of COM2/WFZP

Various studies in the past elucidated the functional role of WFZP and COM2 in maintaining spike inflorescence shape via active suppression of branch outgrowth (Dobrovolskaya et al., 2015; Du et al., 2021; Li, Li, et al., 2021; Poursarebani et al., 2015).

Our genetic and genomic analyses revealed that a single missense mutation (Phe91Leu) in the highly conserved AP2/ERF domain of COM2 is responsible for the massive branch proliferation phenotype in the *rtt1* mutant. Previous studies also identified several COM2 missense variants (AP2/ERF or C‐terminal domains) showing spike branching; however, in all those variants, a proper floret development was established leading to functional floral organs and hence grains (Poursarebani et al., 2015), a key feature completely missing in the *rtt1* mutant. The specification of floret meristem identity in the *rtt1* mutant was partially restored in hybrid plants carrying both *rtt1.a* and *com2.g* alleles, leading to occasional grain set. The restoration of floret meristem identity and spike branch suppression phenotypes in those F_1_ heterozygous genotypes shed light on the fine‐tuned regulation of COM2 in maintaining inflorescence shape and thus grain yield.

Our analysis of the *rtt1* mutation (Phe91Leu) in *COM2* showed no disruption of the protein structure, which, however, exerted a dramatic influence on spike development. This underlines that at the protein level a fine‐tuned regulation is necessary for proper functioning. We identified two key arginines (Arg 68 & 72) on the interaction front, required for proper fitting of the AP2/ERF domain into the major groove of the target DNA. The molecular dynamics simulation of rtt1.a protein gave hints for an unspecific DNA binding as well as misplacement in the DNA groove, affecting the rest of the regulatory machinery.

The analysis of our newly generated hexaploid *WFZP* knockout lines also revealed that supernumerary spikelet formation and spike branch outgrowth was greatly enhanced in plants carrying three mutated *WFZP* alleles (abd, ab*d) compared to single or double allele knockouts, further reinforcing the importance of WFZP dosage in maintaining the spike‐type inflorescence shape of wheat. The presence of functional WFZP protein was demonstrated to be essential, either by the presence of wildtype or in‐frame mutations. The dosage of functional protein was clearly shown by a direct comparison of ab*d* plants with different deletion sizes in the B allele, where plants with the smaller deletion exhibited an advantage over their counterparts with the larger deletion (6 bp vs. 36 bp). In addition, our data showed that a combination of the wild‐type B homoeoallele and the mutant A‐ and D‐copies (*aBd*) is required for better grain formation. Conversely, in‐frame and frameshift mutations present in all three subgenomic copies severely disrupted meristem identity, frequently resulting in sterility. The precise expression of WFZP in wheat is divergent for all three homoeoalleles that exhibit differential expression (Dobrovolskaya et al., 2015). This results in the manifestation of more distinct phenotypes depending on the in‐frame mutated homoeoallele and the number of removed or altered amino acids within the protein. The present study demonstrated a clear dosage‐dependent effect of WFZP, which is consistent with the observations obtained for the barley rtt1.a/com2.g hybrids developed in this study.

The null mutant alleles often result in abnormal inflorescences as evident in the maize *CLAVATA* pathway (Li, Meng, et al., 2022). However, milder knock‐down alleles of these genes can strike a balance between desirable phenotype and optimal yields (Aguirre et al., 2023; Liu et al., 2021; Wang et al., 2021), comparable to our in‐frame *WFZP* mutants or the Leu96Pro substitution in *bh* ^*t*^ *‐A1* (Poursarebani et al., 2015).

Frameshift mutations in the orthologous maize *BD1* led to undetermined branches in place of initial spikelets (Chuck et al., 2002). In rice, various missense mutations in FZP provided clear indications of the precise effects of individual amino acid changes on the *frizzy panicle* phenotype, with the loss of function resulting from a premature stop within the AP2/ERF domain (Komatsu et al., 2003). The observations from maize and rice corroborate those from barley and wheat, thereby substantiating the hypothesis of a conserved regulation of the entire spikelet meristem development among these species. The importance of FZP dosage in the refinement of panicle architecture was demonstrated in rice plants harboring the *small grain and dense panicle 7* (*sgdp7*) mutation, resulting in a reduced FZP dosage, which in turn led to prolonged branch identity specification and, consequently, an augmented grain yield (Bai et al., 2017). Rice FZP translation is regulated by posttranscriptional binding of POLYPYRIMIDINE TRACT‐BINDING PROTEIN 1 and 2 (PTB1/2) to CU‐rich regions within the 3′ UTR of the *FZP* mRNA (Chen, Tian, et al., 2022). Such CU repeats are also evident in the 3′ UTR of *COM2* and all *WFZP* homoeoalleles (Figure S15), suggesting comparable regulation in all three species.

### *rattail 1* mutants show delayed reproductive transition and altered floral fate

Our transcriptome analysis of the *rtt1* mutant showed that the gene repertoire essential for regulating reproductive fate was reprogrammed. The negative regulators of vegetative‐to‐reproductive transition such as *SVP1, 2, 3*, and *CEN* were highly down‐regulated in the *rtt1* mutant, whereas the *SQUAMOSA* genes including *VRN1*, *FUL2*, and *FUL3*, important for timely reproductive transition, were slightly upregulated. It has been shown that *SQUAMOSA* genes promote spikelet identity by repressing *SVP1, 2*, and *3* (Li, Debernardi, et al., 2021). Thus, a strong suppression of these *SVPs* in concert with slightly elevated expression of *SQUAMOSAs* in the *rtt1* mutant is in line with the associated phenotype, where the spikelet meristem identity is perpetually reiterated due to continuous proliferation of inflorescence meristems in place of floret meristems. Also, the *rtt1* mutants showed a higher number of spikelet nodes per spike, delayed heading and shorter plants (Figure S16). Similar phenotypes were also reported in wheat *svp1*, and *vrt2* mutants probably indicating a conserved developmental regulation mediated by these genes across the *Triticeae* (Li, Debernardi, et al., 2021). Notably, *SEP1* and *5*, essential for lemma identity specification (Shen et al., 2024), were also highly down‐regulated in the *rtt1* mutant, which is consistent with failed specification of lemma identity.

### WFZP activity at the whole‐plant level

Our mutant *WFZP* alleles entail specific phenotypes at the spike, and also grain levels, depending on their combination. The combination of the developed *WFZP*‐*A/D* double mutants raised the number of supernumerary spikelets and showed a gain in the number and size of grains. The increase in grain size is contradictory to previous studies that had reported smaller grains in case of *WFZP*‐*A/D* double mutants (Du et al., 2021; Li, Li, et al., 2021). This discrepancy can be attributed to the mutation's location downstream of the AP2/ERF domain, which did not affect the domain structure in our protein predictions (Table S21; TMs see Figure S17). Additionally, we here show for the first time that the single mutation of *WFZP*‐*B* and its combination with mutated *WFZP*‐*A* have a positive influence on the grain yield per plant. Until now, no further mutated *WFZP*‐*B* alleles have been described.

In addition to previous work, the present study demonstrates an impact of WFZP on wheat root development, which was anticipated based on its expression in root apical meristems (Figure S18; Ramirez‐Gonzalez et al., 2018). We detected enhanced root branching and length in the individual *WFZP*‐*A* knockout mutants. This observation contrasts with the effects of *wfzp‐A/D* double mutants, resulting in reduced root length and lateral root numbers (Du et al., 2025). Following work should include the root investigation of all mutant allele combinations generated in this study. These novel findings may have an impact on the yield increase observed in this study and give rise to a deeper look at the influence of below‐ground material on total plant performance. Future studies aiming at increased yield should generally consider both compartments of the plant: the shoot with the inflorescence and the underground located roots.

In summary, the present study delineates the specific function of COM2/WFZP in establishing spikelet meristem fate and grain formation. It was demonstrated that severe mutant alleles of COM2/WFZP result in strong spike branching and altered meristem identity. The specific function of the wheat *WFZP*‐*B* allele was investigated and compared with biallelic *rtt1.a/com2.g* hybrids in barley. This finding, together with the phenotypic effects observed in different mutant homoeoalleles of *WFZP*, indicates a dosage‐dependent functionality of COM2/WFZP. Our simulations of protein molecular dynamics clearly predicted the influence of the highly conserved Phe91Leu substitution in *rattail 1* for the DNA binding ability. Finally, we provided evidence on the impact of WFZP on root branching that needs to be further investigated for its agronomic relevance. In the future, it will be essential to elucidate the intricate regulation of spike branching to develop sustainable crops with augmented yield potential. A systematic approach, entailing precise modifications of individual amino acids within the AP2/ERF domain of WFZP/COM2 and downstream genes, has the potential to generate plants with higher and more stable grain yield. Such an approach should be best executed in elite material and phenotypically investigated in field trials under varying environmental conditions.

## MATERIALS AND METHODS

### Gene IDs in wheat and barley

Barley and wheat gene sequences are available with the following gene IDs: *COM2*—HORVU.MOREX.r3.2HG0114260 (Mascher et al., 2021); *WFZP‐A*—TraesCS2A02G116900; *WFZP‐B*—TraesCS2B02G136100; *WFZP‐D*—TraesCS2D02G118200 (International Wheat Genome Sequencing Consortium (IWGSC), 2018).

### Plant cultivation

Wheat donor material (*Triticum aestivum*, L., spring type breeding line Bobwhite) was cultivated for a period of 13 weeks under controlled conditions at 14/12°C (day/night) with 12 h of light (400–470 μE) and 80% relative humidity. This was followed by a 3–4‐week period in a greenhouse at 18/16°C (day/night) with 16 h of light (200 μE). Regenerated wheat mutants and their progeny were cultivated under controlled conditions for a period of 4–5 weeks at a temperature of 14/12°C (day/night) with 12 h of light (200 μE) and 85% relative humidity, followed by a cultivation phase in greenhouses at 18/15°C (day/night) with 16 h of light.

(200 μE). During the post‐ripening phase, the plants were cultivated at a temperature of 21/19°C (day/night) with 16 h of light (200 μE). Donor and regenerated plants were germinating or transferred initially into Substrate 1 (Klasmann‐Deilmann GmbH, Geeste, Germany) and the developed plantlets were subsequently transferred to a mix of Substrate 2 (Klasmann‐Deilmann GmbH), compost soil and sand (2:2:1). For automatic phenotyping of roots and shoots, the plants were germinating and growing in root phenotyping pots (Shi et al., 2023) filled with Potground P soil (Klasmann‐Deilmann GmbH) under greenhouse conditions at 26/20°C (day/night) with 10 h of light (200 μE) and 33% relative humidity. Plants cultivated in a field‐like environment were initially germinated for 2 weeks as described for regenerated plants and subsequently transferred to native soil in small greenhouses located on the campus of the Leibniz Institute of Plant Genetics and Crop Plant Research (IPK, Seeland, Germany) in the season from March to August 2021. The plants were arranged in plots with 2 × 8 plants per genotype, arranged in consecutive rows. A single row of Bobwhite plants was positioned at the border of each plot.

Barley plants (*Hordeum vulgare*, L.) used for *rtt1.a* mapping, WGS, and RNAseq analysis were grown under controlled greenhouse conditions at IPK, with 18/16°C (day/night) and 16 h of light.

### Genetic analysis of the *rtt1* mutant

The *rattail 1* germplasm of (GSHO216) was maintained in a heterozygous state at USDA, ARS, USA. Fifty‐seven of these individuals were evaluated to identify phenotypic wild‐type (unbranched spike) and mutant (branched spike) plants. Of these, 52 phenotypic wild‐type individuals were identified and their progeny were further screened to identify segregants for wild‐type and mutant phenotypes. Progeny without segregation for spike branching were considered as genotypically wild‐type *RTT1.b* allele types and not continued. Segregating progeny with branched and unbranched spikes were considered genotypically heterozygous (*RTT1.b/rtt1.a*) and used as *rtt1* mapping populations.

### Candidate gene sequencing, *COM2‐* CAPS marker design and co‐segregation analysis in *rtt* mapping populations

For checking the *COM2* sequence variation in *rtt*, DNA from representative wild‐type and mutant plants of the segregating *rtt* population was selected. The cds + 1 kb upstream and downstream regions of start and stop codons were Sanger sequenced. The SNP identified at the cds nucleotide position +273 was converted to a TaqI‐based CAPS marker (Table S2). This marker was used for screening all *rtt1* segregation populations to evaluate the *COM2* genotype and respective phenotype co‐segregation.

### WGS analysis of barley *rtt1*

Whole‐genome shotgun sequencing (WGS) was performed with independent DNA pools prepared from five *RTT* wild‐type and mutant individuals, respectively. The resulting WGS data was analyzed based on the Morex reference genome v2 (Monat et al., 2019) utilizing previously published tools (Li et al., 2009; Danecek et al., 2011; Li, 2013) as described in Methods S1. (Repository ID PRJEB85878).

### Allelism between *rtt1* × *com2*

For allelism analysis, heterozygous individuals of *rtt1* (*RTT1.b*/*rtt1.a*) were crossed with the *com2.g* mutant. The heterozygous individuals at the *rtt1* locus were selected based on the marker genotypes at SNP273 within the *COM2* gene. Thirty‐one F_1s_ from *RTT/rtt* × *com2.g* cross were Sanger sequenced to check the allele types based on *COM2* diagnostic SNPs in *com2.g* (SNP663) and *rtt1.a* (SNP273) (Figure S4). All spikes from the true F_1_ plants were scored either as wild‐type or mutant based on the presence or absence of the spike branching phenotype, and the corresponding *rtt1* and *com2.g* genotype scores were evaluated to reveal the allelism.

### Transcriptome analyses of the barley *rtt1* mutant

RNA was prepared from spike meristems of *RTT1.b and rtt1.a* individuals (10 to 15 per replication; four biological replications) at the lemma primordium stage according to Poursarebani et al. (2020). RNA‐seq was performed using the Illumina platform and sequence reads were analyzed using previously published tools (Bolger et al., 2014; Bray et al., 2016). DEG analysis was conducted based on a published pipeline (Sangket et al., 2022). GO and motif enrichment analysis (Table S22) were done based on previously established pipelines (Bailey et al., 2015; Tang et al., 2023; Zhou et al., 2019). Detailed information is given in Methods S2. (Repository ID PRJEB85879).

### Selection of wheat *WFZP* ‐specific Cas9 target motifs and transformation vector construction

*WFZP*‐specific target motifs conserved across all three homoeoalleles were selected according to Koeppel et al. (2019) and Egorova et al. (2024). Investigation for OFF targets was conducted using the IPK Galaxy Blast Suite (https://galaxy‐web.ipk‐gatersleben.de/).

Target‐specific gRNA/*cas9* vectors (Table S2) were constructed using generic vector pNB38 (Figure S6; GenBank‐ID OR479081) as described in Methods S3. The gRNAs are driven by the wheat *U6* promoter (Shan et al., 2013) and *cas9* of *Streptococcus pyogenes* by the maize *POLYUBIQUITIN 1* promoter. The binary vectors pCH28_WFZP‐TM4 and pCH29_WFZP‐TM5 additionally contain an expression unit for the *hygromycin phosphotransferase* (*hpt*) gene for plant selection. All generated vectors were sequenced by a service provider (LGC Genomics GmbH, Berlin, Germany) using Sanger technology.

### Ballistic DNA transfer to wheat and plant regeneration

The wheat transformation was performed using immature embryos of spring wheat breeding line “Bobwhite” (Warburton et al., 2002; provided by University of Florida) according to the protocol of Ismagul et al. (2014) with some modifications (for detailed description see Methods S4, media composition is given in Table S23).

### Genotypic screening of regenerated wheat plants and their progeny

For plant genotyping, genomic DNA was extracted (Pallotta et al., 2000; Stein et al., 2001) and the targeted region of *WFZP* homoeoalleles amplified using Touchdown‐PCRs with subgenome‐specific primers CH24‐WFZP‐A‐F/CH26‐WFZP‐A‐R for the A copy, CH29‐WFZP‐B‐F/CH31‐WFZP‐B‐R for the B copy, and CH5‐WFZP‐D‐F/CH6‐WFZP‐D‐R for the D copy followed by Sanger sequencing.

The presence or absence of T‐DNAs was validated through amplification of various plasmid regions, including gRNAs (primers TaU6‐F1 and WFZP‐TM4/5‐R), *cas9* (primers Bie475 and zCas9‐R1), and *hpt* (primers 35S‐F2 and Hyg‐R5) (Table S2).

### Crossing and production of doubled haploids in wheat

To obtain higher allelic diversity, crossings were performed between different primary mutants as well as between mutant progeny, and doubled haploids were generated and their ploidy level confirmed (Lippmann et al., 2015; Otto et al., 2015; Table S23; for detailed description see Methods S5).

### Phenotyping of mutagenized wheat plants

The generated *WFZP* mutants were investigated by automatic phenotyping (Junker et al., 2015; Narisetti et al., 2021; Shi et al., 2023) of roots (Table S19, *n* = 7 plants/mutant) and shoots (Tables S19 and S20, *n* = 7 plants/mutant) as well as by manual and semi‐automatic phenotyping of shoot, spike and grain parameters (Tables S14, *n* = 3–7 plants/mutant; Tables S15 and S16, *n* = 10 plants/mutant; Table S17, *n* = 2–12 plants/mutant). Root analysis was performed using *semi‐automated Root Image Analysis* software (saRIA, Narisetti et al., 2019). The corresponding shoot datasets were analyzed using the *Integrated Analysis Platform* software (IAP, Klukas et al., 2014). A detailed description of this procedure is given in Methods S6. (Raw images and image analysis results available at https://doi.org/10.5447/ipk/2026/3).

Statistical outlier values of wheat phenotyping data were identified by calculation of the median absolute deviation (MAD, threshold 2.5×) for each series of measurements. Significant differences were analyzed and figures were designed using SigmaStat 4.0 and SigmaPlot 14.0 software (Systat Software, Inpixon, Palo Alto, CA, USA). The normality of the data was assessed through the application of the Shapiro–Wilk test (Shapiro & Wilk, 1965), while the equality of variance was determined using the Brown–Forsythe test (Brown & Forsythe, 1974). In the event that both tests were passed, a one‐way analysis of variance (ANOVA) with the Holm–Sidak method (Holm, 1979; Šidák, 1967) for multiple comparisons versus the control group was performed. Conversely, in instances where the normality and/or equal variance tests yielded unsatisfactory outcomes, the Kruskal–Wallis test by ranks (Kruskal & Wallis, 1952) was employed and followed by the Dunn's method (Dunn, 1964) for multiple comparisons versus the control group.

### Protein modeling

Protein structural predictions were performed with a local installation of AlphaFold2 (Jumper et al., 2021), except for the models including DNA, which were conducted with AlphaFold3 server (Abramson et al., 2024). Python scripts based on ColabFold code were used to retrieve predicted local distance difference test (pLDDT) confidence scores, multiple sequence alignments, and predicted aligned errors (https://github.com/amandascamara/AlphaFold_analysis). Figures were generated with PyMOL (DeLano, 2002).

Surface charge distribution analyses were done with PDB2PQR and APBS software (Jurrus et al., 2018). Per residue, electrostatic potential was calculated as average for the five models provided in each prediction run of AlphaFold2 (https://github.com/amandascamara/ElectrostaticsPerResidue/).

### Molecular dynamics analysis

The systems were prepared using the CHARMM‐GUI Solution Builder (Jo et al., 2008; Lee et al., 2016; Lee et al., 2020). Protein structures were obtained from AlphaFold2 predictions, and protein–DNA complexes were parameterized with the CHARMM36m force field (Huang et al., 2017). Systems were solvated with the TIP3P water model, a 3‐site rigid water molecule with charges and Lennard–Jones parameters assigned to a single oxygen and two hydrogen atoms (Jorgensen et al., 1983; MacKerell Jr et al., 1998), and neutralizing ions (K+/Cl‐) were added to achieve a 0.15 M KCl concentration, ensuring charge neutrality. A rectangular simulation box with a 10.0 Å edge distance was used, and periodic boundary conditions were applied.

All molecular dynamics simulations were performed in triplicate using NAMD 3.0 (Phillips et al., 2020), following energy minimization, equilibration, and production phases. Minimization was carried out for 10 000 steps using the conjugate gradient method to relax the system. Equilibration was performed in the NVT ensemble at 303.15 K for 1 ns with a Langevin thermostat (damping coefficient: 1 ps^−1^). Unrestrained production simulations were conducted in the NPT ensemble (constant temperature and pressure) for 200 ns at 303.15 K and 1 atm. The temperature was controlled in the simulation with a Langevin thermostat, and pressure with a Langevin piston barostat (damping time: 1 ps^−1^, oscillation period: 50 fs). A 2‐fs time step was used, and the SHAKE algorithm constrained bonds involving hydrogen atoms. Trajectory visualization and rendering were performed using VMD (Humphrey et al., 1996) and PyMol (DeLano, 2002). Average distance between the Cζ atom of residue R68 and the binding site center of mass during molecular dynamics simulations was computed with PyMol.

## AUTHOR CONTRIBUTIONS

CWH, RK, ACS, AJ, TS, and JK designed the research; CWH, RK, VHRN, CM, NB, SH, KA, IMF, YH, AJ, and ASC performed the research; CWH and RK conducted statistical analysis; CWH, RK, VHRN, and ASC wrote the original draft; all authors edited and reviewed the manuscript and approved their authorship.

## CONFLICT OF INTEREST

We declare not to have any conflicts of interest.

## Supporting information


**Figure S1.** Spike meristem developmental time course of *rtt1.a* mutant.
**Figure S2.** WGS mapping of *RTT1.b* and *rtt1.a* reads.
**Figure S3.** Spike phenotypes of new *compositum 2* mutant alleles.
**Figure S4.** Allelism analysis of *rattail 1.a* and *compositum 2.g*.
**Figure S5.** Expression patterns of *SHORT VEGETATIVE PHASE, SQUAMOSA*, and *SEPALLATA MADS* box genes in *RTT1.b* and *rtt1.a*.
**Figure S6.** Generic cloning vector pNB38 (GenBank‐ID OR479081).
**Figure S7.** Genotypic analyses and documentation of *WFZP* primary mutants.
**Figure S8.** Sanger chromatograms of all mutated and inherited *WFZP* mutant alleles.
**Figure S9.** Measured and normalized parameters of plant phenotyping of *WFZP* mutants growing under field‐like conditions.
**Figure S10.** Automatic phenotyping of root and shoot growth of *WFZP* mutants.
**Figure S11.** Disrupted AP2/ERF domains.
**Figure S12.** Bowman and *rattail 1* COM2 AP2/ERF domains predicted in the presence of DNA with AlphaFold3.
**Figure S13.** Electrostatic surface distribution of COM2 AP2/ERF domains of Bowman and *rattail 1*.
**Figure S14.** Arginine hydrogen bonds during molecular dynamics simulation.
**Figure S15.** CU sequence repeats in 3′ UTR of *COM2 and WFZP*.
**Figure S16.** Spike and plant architecture related traits in *RTT1.b* and *rtt1.a*.
**Figure S17.** Coding sequences of *WFZP* homoeoalleles.
**Figure S18.** Root expression of *WFZP* homoeoalleles.
**Table S1.** Germplasm screened for *rtt1* segregation analysis.
**Table S2.** Oligonucleotides and vectors used.
**Table S3.** SNP and deletion mapping in rtt1.a and RTT1.b based on WGS (extra file).
**Table S4.** *irregular spike* mutants with spike branching screened for identifying mutations in *COM2*.
**Table S5.** Differentially expressed genes in the rtt1 mutant (extra file).
**Table S6.** FIMO motif scanning in rtt1‐dependently up‐ and down‐regulated genes (extra file).
**Table S7.** XSTREME motif enrichment in rtt1‐dependently up‐ and down‐regulated genes (extra file).
**Table S8.** Off‐target analyses in wheat.
**Table S9.** Genotyping of primary *WFZP* mutants and their progeny.
**Table S10.** Ploidy and fertility of haploids generated via anther culture.
**Table S11.** Overview of heritable *WFZP* alleles generated.
**Table S12.** Overview of gene products of mutated *WFZP* alleles.
**Table S13.** Overview of non‐inherited *WFZP* alleles generated.
**Table S14.** Grain number per spike from *WFZP* mutants growing under greenhouse conditions.
**Table S15.** Phenotyping of WFZP mutants grown under field‐like conditions (extra file).
**Table S16.** MARVIN grain analysis of WFZP mutants grown under field‐like conditions (extra file).
**Table S17.** Grain yield from WFZP mutants grown under greenhouse conditions (extra file).
**Table S18.** Automatic root phenotyping of WFZP mutants—saRIA‐data (extra file).
**Table S19.** Automatic shoot phenotyping of *WFZP* mutants—fresh and dry mass.
**Table S20.** Automatic shoot phenotyping of WFZP mutants—IAP‐data (extra file).
**Table S21.** Predicted structures of modified COM2 and WFZP proteins (extra file).
**Table S22.** GO enrichment analysis of the rtt1 DEGs (extra file).
**Table S23.** Media composition for ballistic wheat transformation and production of wheat doubled haploids.
**Methods S1.** Whole‐genome shotgun sequencing.
**Methods S2.** Transcriptome studies of barley *rtt1*.
**Methods S3.** Construction of generic vector pNB38 and gRNA/*cas9* vectors.
**Methods S4.** Procedure of ballistic gene transfer to wheat and plant regeneration.
**Methods S5.** Practical hints about crosses and production of doubled haploids in wheat.
**Methods S6.** Wheat plant phenotyping.

**Movie S1.** Molecular dynamics simulation of the protein‐DNA complex in Bowman.

**Movie S2.** Molecular dynamics simulation of the protein‐DNA complex for *rattail 1.a*.

## Data Availability

The accession numbers of the *rattail* plants provided by the USDA are listed in Table S4. The raw data for the *rtt1* WGS (PRJEB85878, https://www.ebi.ac.uk/ena/browser/view/PRJEB85878) and the transcriptome study (PRJEB85879, https://www.ebi.ac.uk/ena/browser/view/PRJEB85879) are available in European Nucleotide Archive (ENA). The pNB38 vector information is stored at NCBI (GenBANK‐ID OR479081). The raw images and image analysis results from the automatic phenotyping of *WFZP* mutants can be found in the e!DAL‐PGP repository (https://doi.org/10.5447/ipk/2026/3). Information about protein models are available on GitHub (https://github.com/amandascamara/AlphaFold_analysis and https://github.com/amandascamara/ElectrostaticsPerResidue/). Further raw data are provided in the Supporting Information.

## References

[tpj71040-bib-0001] Abramson, J. , Adler, J. , Dunger, J. , Evans, J. , Green, T. , Pritzel, A. et al. (2024) Accurate structure prediction of biomolecular interactions with AlphaFold3. Nature, 630, 493–500. Available from: 10.1038/s41586-024-07487-w 38718835 PMC11168924

[tpj71040-bib-0002] Aguirre, L. , Hendelman, A. , Hutton, S.F. , McCandlish, D.M. & Lippman, Z.B. (2023) Idiosyncratic and dose‐dependent epistasis drives variation in tomato fruit size. Science, 382, 315–320. Available from: 10.1126/science.adi5222 37856609 PMC10602613

[tpj71040-bib-0003] Bai, X. , Huang, Y. , Hu, Y. , Liu, H. , Zhang, B. , Smaczniak, C. et al. (2017) Duplication of an upstream silencer of FZP increases grain yield in rice. Nature Plants, 3, 885–893. Available from: 10.1038/s41477-017-0042-4 29085070

[tpj71040-bib-0004] Bailey, T.L. , Johnson, J. , Grant, C.E. & Noble, W.S. (2015) The MEME suite. Nucleic Acids Research, 43, W39–W49. Available from: 10.1093/nar/gkv416 25953851 PMC4489269

[tpj71040-bib-0005] Barros, J. , Serk, H. , Granlund, I. & Pesquet, E. (2015) The cell biology of lignification in higher plants. Annals of Botany, 115, 1053–1074. Available from: 10.1093/aob/mcv046 25878140 PMC4648457

[tpj71040-bib-0006] Bolger, A.M. , Lohse, M. & Usadel, B. (2014) Trimmomatic: a flexible trimmer for Illumina sequence data. Bioinformatics, 30, 2114–2120. Available from: 10.1093/bioinformatics/btu170 24695404 PMC4103590

[tpj71040-bib-0007] Bray, N.L. , Pimentel, H. , Melsted, P. & Pachter, L. (2016) Near‐optimal probabilistic RNA‐seq quantification. Nature Biotechnology, 34, 525–527. Available from: 10.1038/nbt.3519 27043002

[tpj71040-bib-0008] Brown, M.B. & Forsythe, A.B. (1974) The small sample behavior of some statistics which test the equality of several means. Technometrics, 16, 129–132. Available from: 10.1080/00401706.1974.10489158

[tpj71040-bib-0009] Budhagatapalli, N. , Halbach, T. , Hiekel, S. , Buchner, H. , Müller, A.E. & Kumlehn, J. (2020) Site‐directed mutagenesis in bread and durum wheat via pollination by cas9/guide RNA‐transgenic maize used as haploidy inducer. Plant Biotechnology Journal, 18, 2376–2378. Available from: 10.1111/pbi.13415 32426943 PMC7680543

[tpj71040-bib-0010] Chen, L. , Jameson, G.B. , Guo, Y. , Song, J. & Jameson, P.E. (2022) The LONELY GUY gene family: from mosses to wheat, the key to the formation of active cytokinins in plants. Plant Biotechnology Journal, 20, 625–645. Available from: 10.1111/pbi.13783 35108444 PMC8989509

[tpj71040-bib-0011] Chen, Q. , Tian, F. , Cheng, T. , Jiang, J. , Zhu, G. , Gao, Z. et al. (2022) Translational repression of FZP mediated by CU‐rich element/OsPTB interactions modulates panicle development in rice. The Plant Journal, 110, 1319–1331. Available from: 10.1111/tpj.15737 35293072

[tpj71040-bib-0012] Chuck, G. , Muszynski, M. , Kellogg, E. , Hake, S. & Schmidt, R.J. (2002) The control of spikelet meristem identity by the branched silkless1 gene in maize. Science, 298, 1238–1241. Available from: 10.1126/science.1076920 12424380

[tpj71040-bib-0013] Danecek, P. , Auton, A. , Abecasis, G. , Albers, C.A. , Banks, E. , DePristo, M.A. et al. (2011) The variant call format and VCFtools. Bioinformatics, 27, 2156–2158. Available from: 10.1093/bioinformatics/btr330 21653522 PMC3137218

[tpj71040-bib-0014] DeLano, W.L. (2002) Pymol: an open‐source molecular graphics tool. CCP4 Newsletter on Protein Crystallography, 40, 82–92. Available from: 10.1093/bioinformatics/btae139

[tpj71040-bib-0015] Derbyshire, P. & Byrne, M.E. (2013) MORE SPIKELETS1 is required for spikelet fate in the inflorescence of Brachypodium. Plant Physiology, 161, 1291–1302. Available from: 10.1104/pp.112.212340 23355632 PMC3585597

[tpj71040-bib-0016] Dobrovolskaya, O. , Pont, C. , Sibout, R. , Martinek, P. , Badaeva, E. , Murat, F. et al. (2015) FRIZZY PANICLE drives supernumerary spikelets in bread wheat. Plant Physiology, 167, 189–199. Available from: 10.1104/pp.114.250043 25398545 PMC4281007

[tpj71040-bib-0017] Dobrovolskaya, O.B. , Amagai, Y. , Popova, K.I. , Dresvyannikova, A.E. , Martinek, P. , Krasnikov, A.A. et al. (2017) Genes WHEAT FRIZZY PANICLE and SHAM RAMIFICATION 2 independently regulate differentiation of floral meristems in wheat. BMC Plant Biology, 17, 252. Available from: 10.1186/s12870-017-1191-3 29297328 PMC5751757

[tpj71040-bib-0018] Du, D. , Li, Z. , Jiang, Z. , Yuan, J. , Zhang, X. , Zhao, H. et al. (2025) The transcription factor WFZP interacts with the chromatin remodeler TaSYD to regulate root architecture and nitrogen uptake efficiency in wheat. Advanced Science, 12, 2416433. Available from: 10.1002/advs.202416433 39992804 PMC12005776

[tpj71040-bib-0019] Du, D. , Zhang, D. , Yuan, J. , Feng, M. , Li, Z. , Wang, Z. et al. (2021) FRIZZY PANICLE defines a regulatory hub for simultaneously controlling spikelet formation and awn elongation in bread wheat. New Phytologist, 231, 814–833. Available from: 10.1111/nph.17388 33837555

[tpj71040-bib-0020] Dunn, O.J. (1964) Multiple comparisons using rank sums. Technometrics, 6, 241–252. Available from: 10.1080/00401706.1964.10490181

[tpj71040-bib-0021] Egorova, A.A. , Zykova, T.E. , Hertig, C.W. , Hoffie, I. , Morozov, S.V. , Chernyak, E.I. et al. (2024) Accumulation of anthocyanin in the Aleurone of barley grains by targeted restoration of the MYC2 gene. International Journal of Molecular Sciences, 25, 12705. Available from: 10.3390/ijms252312705 39684416 PMC11641404

[tpj71040-bib-0022] Eklöf, J.M. & Brumer, H. (2010) The XTH gene family: an update on enzyme structure, function, and phylogeny in xyloglucan remodeling. Plant Physiology, 153, 456–466. Available from: 10.1104/pp.110.156844 20421457 PMC2879796

[tpj71040-bib-0023] Ferrante, A. , Cartelle, J. , Savin, R. & Slafer, G.A. (2017) Yield determination, interplay between major components and yield stability in a traditional and a contemporary wheat across a wide range of environments. Field Crops Research, 203, 114–127. Available from: 10.1016/j.fcr.2016.12.028

[tpj71040-bib-0024] Gerasimova, S.V. , Hertig, C. , Korotkova, A.M. , Kolosovskaya, E.V. , Otto, I. , Hiekel, S. et al. (2020) Conversion of hulled into naked barley by Cas endonuclease‐mediated knockout of the NUD gene. BMC Plant Biology, 20, 255. Available from: 10.1186/s12870-020-02454-9 33050877 PMC7556925

[tpj71040-bib-0025] Gou, M. , Ran, X. , Martin, D.W. & Liu, C.J. (2018) The scaffold proteins of lignin biosynthetic cytochrome P450 enzymes. Nature Plants, 4, 299–310. Available from: 10.1038/s41477-018-0142-9 29725099

[tpj71040-bib-0026] Gou, X. , Feng, X. , Shi, H. , Guo, T. , Xie, R. , Liu, Y. et al. (2022) PPVED: a machine learning tool for predicting the effect of single amino acid substitution on protein function in plants. Plant Biotechnology Journal, 20, 1417–1431. Available from: 10.1111/pbi.13823 35398963 PMC9241370

[tpj71040-bib-0027] Guo, Z. , Chen, D. , Alqudah, A.M. , Röder, M.S. , Ganal, M.W. & Schnurbusch, T. (2017) Genome‐wide association analyses of 54 traits identified multiple loci for the determination of floret fertility in wheat. New Phytologist, 214, 257–270. Available from: 10.1111/nph.14342 27918076

[tpj71040-bib-0028] Holm, S. (1979) A simple sequentially rejective multiple test procedure. Scandinavian Journal of Statistics, 6, 65–70.

[tpj71040-bib-0029] Huang, J. , Rauscher, S. , Nawrocki, G. , Ran, T. , Feig, M. , de Groot, B.L. et al. (2017) CHARMM36m: an improved force field for folded and intrinsically disordered proteins. Nature Methods, 14, 71–73. Available from: 10.1038/nmeth.4067 27819658 PMC5199616

[tpj71040-bib-0030] Huang, Y. , Zhao, S. , Fu, Y. , Sun, H. , Ma, X. , Tan, L. et al. (2018) Variation in the regulatory region of FZP causes increases in secondary inflorescence branching and grain yield in rice domestication. The Plant Journal, 96, 716–733. Available from: 10.1111/tpj.14062 30101570

[tpj71040-bib-0031] Humphrey, W. , Dalke, A. & Schulten, K. (1996) VMD—visual molecular dynamics. Journal of Molecular Graphics, 14, 33–38. Available from: 10.1016/0263-7855(96)00018-5 8744570

[tpj71040-bib-0032] Ikeda, K. , Ito, M. , Nagasawa, N. , Kyozuka, J. & Nagato, Y. (2007) Rice ABERRANT PANICLE ORGANIZATION 1, encoding an F‐box protein, regulates meristem fate. The Plant Journal, 51, 1030–1040. Available from: 10.1111/j.1365-313X.2007.03200.x 17666027

[tpj71040-bib-0033] International Wheat Genome Sequencing Consortium (IWGSC) . (2018) Shifting the limits in wheat research and breeding using a fully annotated reference genome. Science, 361, eaar7191. Available from: 10.1126/science.aar7191 30115783

[tpj71040-bib-0034] Ismagul, A. , Iskakova, G. , Harris, J.C. & Eliby, S. (2014) Biolistic transformation of wheat with Centrophenoxine as a synthetic auxin. Methods in Molecular Biology, 1145, 191–202. Available from: 10.1007/978-1-4939-0446-4_15 24816669

[tpj71040-bib-0035] Jo, S. , Kim, T. , Iyer, V.G. & Im, W. (2008) CHARMM‐GUI: a web‐based graphical user interface for CHARMM. Journal of Computational Chemistry, 29, 1859–1865. Available from: 10.1002/jcc.20945 18351591

[tpj71040-bib-0036] Jorgensen, W.L. , Chandrasekhar, J. , Madura, J.D. , Impey, R.W. & Klein, M.L. (1983) Comparison of simple potential functions for simulating liquid water. The Journal of Chemical Physics, 79, 926–935. Available from: 10.1063/1.445869

[tpj71040-bib-0037] Jumper, J. , Evans, R. , Pritzel, A. , Green, T. , Figurnov, M. , Ronneberger, O. et al. (2021) Highly accurate protein structure prediction with AlphaFold. Nature, 596, 583–589. Available from: 10.1038/s41586-021-03819-2 34265844 PMC8371605

[tpj71040-bib-0038] Junker, A. , Muraya, M.M. , Weigelt‐Fischer, K. , Arana‐Ceballos, F. , Klukas, C. , Melchinger, A.E. et al. (2015) Optimizing experimental procedures for quantitative evaluation of crop plant performance in high throughput phenotyping system. Frontiers in Plant Science, 5, 770. Available from: 10.3389/fpls.2014.00770 25653655 PMC4299434

[tpj71040-bib-0039] Jurrus, E. , Engel, D. , Star, K. , Monson, K. , Brandi, J. , Felberg, L.E. et al. (2018) Improvements to the APBS biomolecular solvation software suite. Protein Science, 27, 112–128. Available from: 10.1002/pro.3280 28836357 PMC5734301

[tpj71040-bib-0040] Klukas, C. , Chen, D. & Pape, J.M. (2014) Integrated analysis platform: an open‐source information system for high‐throughput plant phenotyping. Plant Physiology, 165, 506–518. Available from: 10.1104/pp.113.233932 24760818 PMC4044849

[tpj71040-bib-0041] Koeppel, I. , Hertig, C. , Hoffie, R. & Kumlehn, J. (2019) Cas Endonuklease technology—a quantum leap in the advancement of barley and wheat genetic engineering. International Journal of Molecular Sciences, 20, 2647. Available from: 10.3390/ijms20112647 31146387 PMC6600890

[tpj71040-bib-0042] Komatsu, M. , Chujo, A. , Nagato, Y. , Shimamoto, K. & Kyozuka, J. (2003) FRIZZY PANICLE is required to prevent the formation of axillary meristems and to establish floral meristem identity in rice spikelets. Development, 130, 3841–3850. Available from: 10.1242/dev.00564 12835399

[tpj71040-bib-0043] Kruskal, W.H. & Wallis, W.A. (1952) Use of ranks in one‐criterion variance analysis. Journal of the American Statistical Association, 47, 583–621. Available from: 10.1080/01621459.1952.10483441

[tpj71040-bib-0044] Lee, J. , Cheng, X. , Swails, J.M. , Yeom, M.S. , Eastman, P.K. , Lemkul, J.A. et al. (2016) CHARMM‐GUI input generator for NAMD, GROMACS, AMBER, OpenMM, and CHARMM/OpenMM simulations using the CHARMM36 additive force field. Journal of Chemical Theory and Computation, 12, 405–413. Available from: 10.1021/acs.jctc.5b00935 26631602 PMC4712441

[tpj71040-bib-0045] Lee, J. , Hitzenberger, M. , Rieger, M. , Kern, N.R. , Zacharias, M. & Im, W. (2020) CHARMM‐GUI supports the Amber force fields. Journal of Chemical Physics, 153, 035103. Available from: 10.1063/5.0012280 32716185

[tpj71040-bib-0046] Li, C. , Lin, H. , Chen, A. , Lau, M. , Jernstedt, J. & Dubcovsky, J. (2019) Wheat VRN1, FUL2 and FUL3 play critical and redundant roles in spikelet development and spike determinacy. Development, 146, dev175398. Available from: 10.1242/dev.175398 31337701 PMC6679363

[tpj71040-bib-0047] Li, H. (2013) Aligning sequence reads, clone sequences and assembly contigs with BWA‐MEM. *arXiv*:1303.3997. 10.48550/arXiv.1303.3997

[tpj71040-bib-0048] Li, H. , Handsaker, B. , Wysoker, A. , Fennell, T. , Ruan, J. , Homer, N. et al. (2009) The sequence alignment/map format and SAMtools. Bioinformatics, 25, 2078–2079. Available from: 10.1093/bioinformatics/btp352 19505943 PMC2723002

[tpj71040-bib-0049] Li, J. , Zhang, L. , Elbaiomy, R.G. , Chen, L. , Wang, Z. , Jiao, J. et al. (2022) Evolution analysis of FRIZZY PANICLE (FZP) orthologs explored the mutations in DNA coding sequences in the grass family (Poaceae). PeerJ, 10, e12880. Available from: 10.7717/peerj.12880 35295554 PMC8919851

[tpj71040-bib-0050] Li, K. , Debernardi, J.M. , Li, C. , Lin, H. , Zhang, C. , Jernstedt, J. et al. (2021) Interactions between SQUAMOSA and SHORT VEGETATIVE PHASE MADS‐box proteins regulate meristem transitions during wheat spike development. The Plant Cell, 33, 3621–3644. Available from: 10.1093/plcell/koab243 34726755 PMC8643710

[tpj71040-bib-0051] Li, S. , Meng, S. , Weng, J. & Wu, Q. (2022) Fine‐tuning shoot meristem size to feed the world. Trends in Plant Science, 27, 355–363. Available from: 10.1016/j.tplants.2021.10.004 34743928

[tpj71040-bib-0052] Li, Y. , Li, L. , Zhao, M. , Guo, L. , Guo, X. , Zhao, D. et al. (2021) Wheat FRIZZY PANICLE activates VERNALIZATION1‐a and HOMEOBOX4‐a to regulate spike development in wheat. Plant Biotechnology Journal, 19, 1141–1154. Available from: 10.1111/pbi.13535 33368973 PMC8196646

[tpj71040-bib-0053] Lin, X. , Xu, Y. , Wang, D. , Yang, Y. , Zhang, X. , Bie, X. et al. (2024) Systematic identification of wheat spike developmental regulators by integrated multi‐omics, transcriptional network, GWAS, and genetic analyses. Molecular Plant, 17, 438–459. Available from: 10.1016/j.molp.2024.01.010 38310351

[tpj71040-bib-0054] Lippmann, R. , Friedel, S. , Mock, H.P. & Kumlehn, J. (2015) The low molecular weight fraction of compounds released from immature wheat pistils supports barley pollen embryogenesis. Frontiers in Plant Science, 6, 498. Available from: 10.3389/fpls.2015.00498 26217352 PMC4493395

[tpj71040-bib-0055] Liu, L. , Gallagher, J. , Arevalo, E.D. , Chen, R. , Skopelitis, T. , Wu, Q. et al. (2021) Enhancing grain‐yield‐related traits by CRISPR‐Cas9 promoter editing of maize CLE genes. Nature Plants, 7, 287–294. Available from: 10.1038/s41477-021-00858-5 33619356

[tpj71040-bib-0056] MacKerell, A.D., Jr. , Bashford, D. , Bellott, M. , Dunbrack, R.L. , Evanseck, J.D. , Field, M.J. et al. (1998) All‐atom empirical potential for molecular modelling and dynamics studies of proteins. Journal of Physical Chemistry B, 102, 3586–3616. Available from: 10.1021/jp973084f 24889800

[tpj71040-bib-0057] Mascher, M. , Wicker, T. , Jenkins, J. , Plott, C. , Lux, T. , Koh, C.S. et al. (2021) Long‐read sequence assembly: a technical evaluation in barley. The Plant Cell, 33, 1888–1906. Available from: 10.1093/plcell/koab077 33710295 PMC8290290

[tpj71040-bib-0058] Mitsuda, N. , Seki, M. , Shinozaki, K. & Ohme‐Takagi, M. (2005) The NAC transcription factors NST1 and NST2 of Arabidopsis regulate secondary wall thickenings and are required for anther dehiscence. The Plant Cell, 17, 2993–3006. Available from: 10.1105/tpc.105.036004 16214898 PMC1276025

[tpj71040-bib-0059] Monat, C. , Padmarasu, S. , Lux, T. , Wicker, T. , Gundlach, H. , Himmelbach, A. et al. (2019) TRITEX: chromosome‐scale sequence assembly of Triticeae genomes with open‐source tools. Genome Biology, 20, 284. Available from: 10.1186/s13059-019-1899-5 31849336 PMC6918601

[tpj71040-bib-0060] Moon, S.Y. & Zheng, Y. (2003) Rho GTPase‐activating proteins in cell regulation. Trends in Cell Biology, 13, 13–22. Available from: 10.1016/s0962-8924(02)00004-1 12480336

[tpj71040-bib-0061] Narisetti, N. , Henke, M. , Seiler, C. , Junker, A. , Ostermann, J. , Altmann, T. et al. (2021) Fully‐automated root image analysis (faRIA). Scientific Reports, 11, 16047. Available from: 10.1038/s41598-021-95480-y 34362967 PMC8346561

[tpj71040-bib-0062] Narisetti, N. , Henke, M. , Seiler, C. , Shi, R. , Junker, A. , Altmann, T. et al. (2019) Semi‐automated root image analysis (saRIA). Scientific Reports, 9, 19674. Available from: 10.1038/s41598-019-55876-3 31873104 PMC6928233

[tpj71040-bib-0063] Otto, I. , Müller, A. & Kumlehn, J. (2015) Barley (*Hordeum vulgare* L.) transformation using embryogenic pollen cultures. Methods in Molecular Biology, 1223, 85–99. Available from: 10.1007/978-1-4939-1695-5_7 25300833

[tpj71040-bib-0064] Pallotta, M.A. , Graham, R.D. , Langridge, P. , Sparrow, D.H.B. & Barker, S.J. (2000) RFLP mapping of manganese efficiency in barley. Theoretical and Applied Genetics, 101, 1100–1108. Available from: 10.1007/s001220051585

[tpj71040-bib-0065] Phillips, J.C. , Hardy, D.J. , Maia, J.D.C. , Stone, J.E. , Ribeiro, J.V. , Bernardi, R.C. et al. (2020) Scalable molecular dynamics on CPU and GPU architectures with NAMD. The Journal of Chemical Physics, 153, 44130. Available from: 10.1063/5.0014475 PMC7395834 32752662

[tpj71040-bib-0066] Poursarebani, N. , Seidensticker, T. , Koppolu, R. , Trautewig, C. , Gawronski, P. , Bini, F. et al. (2015) The genetic basis of composite spike form in barley and ‘miracle‐wheat’. Genetics, 201, 155–165. Available from: 10.1534/genetics.115.176628 26156223 PMC4566260

[tpj71040-bib-0067] Poursarebani, N. , Trautewig, C. , Melzer, M. , Nussbaumer, T. , Lundqvist, U. , Rutten, T. et al. (2020) COMPOSITUM 1 contributes to the architectural simplification of barley inflorescence via meristem identity signals. Nature Communications, 11, 5138. Available from: 10.1038/s41467-020-18890-y PMC7550572 33046693

[tpj71040-bib-0068] Ramirez‐Gonzalez, R.H. , Borrill, P. , Lang, D. , Harrington, S.A. , Brinton, J. , Venturini, L. et al. (2018) The transcriptional landscape of polyploid wheat. Science, 361, eaar6089. Available from: 10.1126/science.aar6089 30115782

[tpj71040-bib-0069] Ray, D.K. , Mueller, N.D. , West, P.C. & Foley, J.A. (2013) Yield trends are insufficient to double global crop production by 2050. PLoS One, 8, e66428. Available from: 10.1371/journal.pone.0066428 23840465 PMC3686737

[tpj71040-bib-0070] Robertson, D.W. (1967) Linkage studies of various barley mutations (*Hordeum* species). Crop Science, 7, 41–42. Available from: 10.2135/cropsci1967.0011183X000700010015x

[tpj71040-bib-0071] Sakuma, S. , Golan, G. , Guo, Z. , Ogawa, T. , Tagiri, A. , Sugimoto, K. et al. (2019) Unleashing floret fertility in wheat through the mutation of a homeobox gene. PNAS, 116, 5182–5187. Available from: 10.1073/pnas.1815465116 30792353 PMC6421441

[tpj71040-bib-0072] Sanchez‐Garcia, M. , Royo, C. , Aparicio, N. , Martin‐Sanchez, J.A. & Alvaro, F. (2013) Genetic improvement of bread wheat yield and associated traits in Spain during the 20^th^ century. Journal of Agricultural Science, 151, 105–118. Available from: 10.1017/s0021859612000330 23365469 PMC3518273

[tpj71040-bib-0073] Sangket, U. , Yodsawat, P. , Nuanpirom, J. & Sathapondecha, P. (2022) bestDEG: a web‐based application automatically combines various tools to precisely predict differentially expressed genes (DEGs) from RNA‐Seq data. PeerJ, 10, e14344. Available from: 10.7717/peerj.14344 36389403 PMC9657178

[tpj71040-bib-0074] Satoh‐Nagasawa, N. , Nagasawa, N. , Malcomber, S. , Sakai, H. & Jackson, D. (2006) A trehalose metabolic enzyme controls inflorescence architecture in maize. Nature, 441, 227–230. Available from: 10.1038/nature04725 16688177

[tpj71040-bib-0075] Selva, C. , Shirley, N.J. , Houston, K. , Whitford, R. , Baumann, U. , Li, G. et al. (2021) HvLEAFY controls the early stages of floral organ specification and inhibits the formation of multiple ovaries in barley. The Plant Journal, 108, 509–527. Available from: 10.1111/tpj.15457 34382710

[tpj71040-bib-0076] Shan, Q. , Wang, Y. , Li, J. , Zhang, Y. , Chen, K. , Liang, Z. et al. (2013) Targeted genome modification of crop plants using a CRISPR‐Cas system. Nature Biotechnology, 31, 686–688. Available from: 10.1038/nbt.2650 23929338

[tpj71040-bib-0077] Shapiro, S.S. & Wilk, M.B. (1965) An analysis of variance test for normality. Biometrika, 52, 591–611. Available from: 10.1093/biomet/52.3-4.591

[tpj71040-bib-0078] Shen, C. , Yang, X. , Wang, D. , Li, G. & Tucker, M.R. (2024) Functional retrogression of LOFSEPs in specifying floral organs in barley. aBIOTECH, 6, 1–11. Available from: 10.1007/s42994-024-00182-4 40060184 PMC11889289

[tpj71040-bib-0079] Shi, R. , Seiler, C. , Knoch, D. , Junker, A. & Altmann, T. (2023) Integrated phenotyping of root and shoot growth dynamics in maize reveals specific interaction patterns in inbreds and hybrids and in response to drought. Frontiers in Plant Science, 14, 1233553. Available from: 10.3389/fpls.2023.1233553 37719228 PMC10502302

[tpj71040-bib-0080] Šidák, Z. (1967) Rectangular confidence regions for the means of multivariate normal distributions. Journal of the American Statistical Association, 62, 626–633. Available from: 10.1080/01621459.1967.10482935

[tpj71040-bib-0081] Stein, N. , Herren, G. & Keller, B. (2001) A new DNA extraction method for high‐throughput marker analysis in a large‐genome species such as *Triticum aestivum* . Plant Breeding, 120, 354–356. Available from: 10.1046/j.1439-0523.2001.00615.x

[tpj71040-bib-0082] Tang, D. , Chen, M. , Huang, X. , Zhang, G. , Zeng, L. , Zhang, G. et al. (2023) SRplot: a free online platform for data visualization and graphing. PLoS One, 18, e0294236. Available from: 10.1371/journal.pone.0294236 37943830 PMC10635526

[tpj71040-bib-0083] Thiel, J. , Koppolu, R. , Trautewig, C. , Hertig, C. , Kale, S.M. , Erbe, S. et al. (2021) Transcriptional landscapes of floral meristems in barley. Science Advances, 7, eabf0832. Available from: 10.1126/sciadv.abf0832 33910893 PMC8081368

[tpj71040-bib-0084] Van der Does, D. , Boutrot, F. , Engelsdorf, T. , Rhodes, J. , McKenna, J.F. , Vernhettes, S. et al. (2017) The Arabidopsis leucine‐rich repeat receptor kinase MIK2/LRR‐KISS connects cell wall integrity sensing, root growth and response to abiotic and biotic stresses. PLoS Genetics, 13, e1006832. Available from: 10.1371/journal.pgen.1006832 28604776 PMC5484538

[tpj71040-bib-0085] Wang, W. , Pan, Q. , He, F. , Akhunova, A. , Chao, S. , Trick, H. et al. (2018) Transgenerational CRISPR‐Cas9 activity facilitates multiplex gene editing in allopolyploid wheat. CRISPR Journal, 1, 65–74. Available from: 10.1089/crispr.2017.0010 30627700 PMC6319321

[tpj71040-bib-0086] Wang, W. , Simmonds, J. , Pan, Q. , Davidson, D. , He, F. , Battal, A. et al. (2018) Gene editing and mutagenesis reveal inter‐cultivar differences and additivity in the contribution of TaGW2 homoeologues to grain size and weight in wheat. Theoretical and Applied Genetics, 131, 2463–2475. Available from: 10.1007/s00122-018-3166-7 30136108 PMC6208945

[tpj71040-bib-0087] Wang, X. , Aguirre, L. , Rodriguez‐Leal, D. , Hendelman, A. , Benoit, M. & Lippman, Z.B. (2021) Dissecting cis‐regulatory control of quantitative trait variation in a plant stem cell circuit. Nature Plants, 7, 419–427. Available from: 10.1038/s41477-021-00898-x 33846596

[tpj71040-bib-0088] Wang, Y. , Du, F. , Wang, J. , Wang, K. , Tian, C. , Qi, X. et al. (2022) Improving bread wheat yield through modulating an unselected AP2/ERF gene. Nature Plants, 8, 930–939. Available from: 10.1038/s41477-022-01197-9 35851621

[tpj71040-bib-0089] Warburton, L. , Skovmand, B. & Mujeeb‐Kazi, A. (2002) The molecular genetic characterization of the ‘bobwhite’ bread wheat family using AFLPs and the effect of the T1BL.1RS translocation. Theoretical and Applied Genetics, 104, 868–873. Available from: 10.1007/s00122-001-0816-x 12582648

[tpj71040-bib-0090] Wolde, G.M. & Schnurbusch, T. (2019) Inferring vascular architecture of the wheat spikelet based on resource allocation in the branched head(t) (bh(t)‐A1) near isogenic lines. Functional Plant Biology, 46, 1023–1035. Available from: 10.1071/FP19041 32172750

[tpj71040-bib-0091] Youssefian, S. , Kirby, E.J.M. & Gale, M.D. (1992a) Pleiotropic effects of the GA‐insensitive Rht dwarfing genes in wheat. 1. Effects on development of the ear, stem and leaves. Field Crops Research, 28, 179–190. Available from: 10.1016/0378-4290(92)90039-C

[tpj71040-bib-0092] Youssefian, S. , Kirby, E.J.M. & Gale, M.D. (1992b) Pleiotropic effects of the GA‐insensitive Rht dwarfing genes in wheat. 2. Effects on leaf, stem, ear and floret growth. Field Crops Research, 28, 191–210. Available from: 10.1016/0378-4290(92)90040-G

[tpj71040-bib-0093] Zhang, Y. , Shen, C. , Li, G. , Shi, J. , Yuan, Y. , Ye, L. et al. (2024) MADS1‐regulated lemma and awn development benefits barley yield. Nature Communications, 15, 301. Available from: 10.1038/s41467-023-44457-8 PMC10770128 38182608

[tpj71040-bib-0094] Zhou, H. , Hu, J. , Wang, M. , Wang, X. & Chen, S. (2023) Gene locus mapping and candidate gene screening for branched spike and its associated traits of the Ynbs mutant in barley. Agriculture, 13, 1934. Available from: 10.3390/agriculture13081934

[tpj71040-bib-0095] Zhou, Y. , Zhou, B. , Pache, L. , Chang, M. , Khodabakhshi, A.H. , Tanaseichuk, O. et al. (2019) Metascape provides a biologist‐oriented resource for the analysis of systems‐level datasets. Nature Communications, 10, 1523. Available from: 10.1038/s41467-019-09234-6 PMC6447622 30944313

[tpj71040-bib-0096] Zhu, Q.H. , Hoque, M.S. , Dennis, E.S. & Upadhyaya, N.M. (2003) Ds tagging of BRANCHED FLORETLESS 1 (BFL1) that mediates the transition from spikelet to floret meristem in rice (*Oryza sativa* L). BMC Plant Biology, 3, 6. Available from: 10.1186/1471-2229-3-6 14503923 PMC270090

