# Supplementary material for: COMPOSITUM 2/ WHEAT FRIZZY PANICLE dosage dependently regulates inflorescence and root architecture in Triticeae cereals
Source: Plant J. 2026 Jul 23;127(2):e71040. doi: 10.1111/tpj.71040 (PMC13394734; doi:10.1111/tpj.71040)
Supplement: Supplementary file 1 — Figure S1. Spike meristem developmental time course of rtt1.a mutant. Figure S2. WGS mapping of RTT1.b and rtt1.a reads. Figure S3. Spike phenotypes of new compositum 2 mutant alleles. Figure S4. Allelism analysis of rattail 1.a and compositum 2.g. Figure S5. Expression patterns of SHORT VEGETATIVE PHASE, SQUAMOSA, and SEPALLATA MADS box genes in RTT1.b and rtt1.a. Figure S6. Generic cloning vector pNB38 (GenBank‐ID OR479081). Figure S7. Genotypic analyses and documentation of WFZP primary mutants. Figure S8. Sanger chromatograms of all mutated and inherited WFZP mutant alleles. Figure S9. Measured and normalized parameters of plant phenotyping of WFZP mutants growing under field‐like conditions. Figure S10. Automatic phenotyping of root and shoot growth of WFZP mutants. Figure S11. Disrupted AP2/ERF domains. Figure S12. Bowman and rattail 1 COM2 AP2/ERF domains predicted in the presence of DNA with AlphaFold3. Figure S13. Electrostatic surface distribution of COM2 AP2/ERF domains of Bowman and rattail 1. Figure S14. Arginine hydrogen bonds during molecular dynamics simulation. Figure S15. CU sequence repeats in 3′ UTR of COM2 and WFZP. Figure S16. Spike and plant architecture related traits in RTT1.b and rtt1.a. Figure S17. Coding sequences of WFZP homoeoalleles. Figure S18. Root expression of WFZP homoeoalleles. Table S1. Germplasm screened for rtt1 segregation analysis. Table S2. Oligonucleotides and vectors used. Table S3. SNP and deletion mapping in rtt1.a and RTT1.b based on WGS (extra file). Table S4. irregular spike mutants with spike branching screened for identifying mutations in COM2. Table S5. Differentially expressed genes in the rtt1 mutant (extra file). Table S6. FIMO motif scanning in rtt1‐dependently up‐ and down‐regulated genes (extra file). Table S7. XSTREME motif enrichment in rtt1‐dependently up‐ and down‐regulated genes (extra file). Table S8. Off‐target analyses in wheat. Table S9. Genotyping of primary WFZP mutants and their progeny. Table S1 [file TPJ-127-0-s002.zip › tpj71040-sup-0001-FigureS1-S18-TableS1-S23.docx]

***COMPOSITUM 2*/ *WHEAT FRIZZY PANICLE* dosage-dependently regulates inflorescence and root architecture in *Triticeae* cereals**

Christian W. Hertig, Ravi Koppolu, Victor H. Rabesquine Nogueira, Cornelia Marthe, Nagaveni Budhagatapalli, Stefan Hiekel, Kavya Amte, Irene M. Fontana, Yongyu Huang, Astrid Junker, Amanda S. Câmara, Thorsten Schnurbusch, Jochen Kumlehn

**Supporting Information**

1. *Supporting figures*


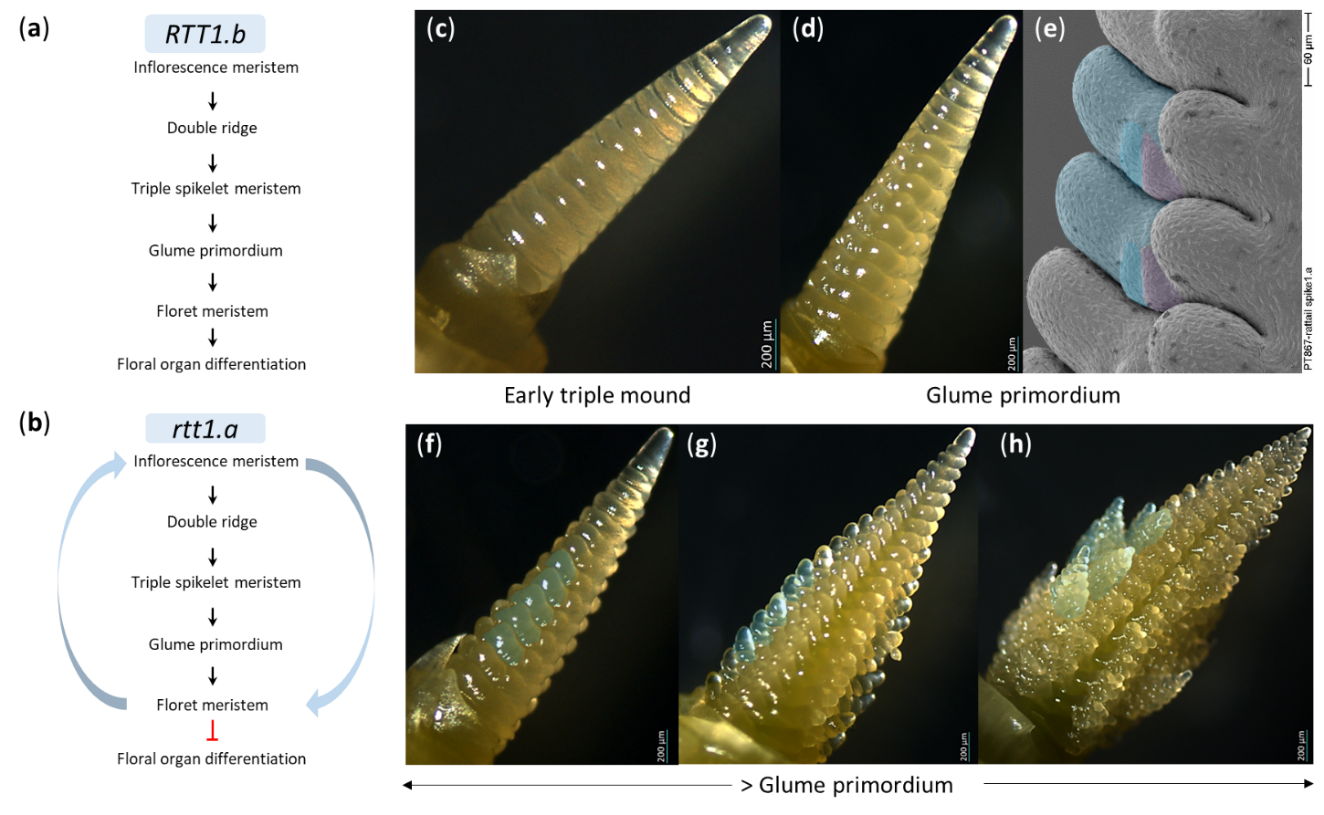


**Figure S1** Spike meristem developmental time course of *rtt1.a* mutant; (a,b) Developmental chronology of wild-type (Bowman) (a; *RTT1.b*) and mutant (b; *rtt1.a*) spikes. (c-e) *rtt1.a* spike meristems at early triple mound (c) and glume primordia (GP) stages (d). (e) Scanning electron micrograph of *rtt1.a* spike meristem showing additional mound in the glume primordium (glume primordium in purple, additional mound in dark blue, presumptive floret meristem in light blue). (f-h) *rtt1.a* spike meristems at various stages after GP initiation. The blue highlighted portions from central spikelet region depict the presumptive floret meristems transitioned to inflorescence-like meristems.

**Figure S2** WGS mapping of *RTT1.b* and *rtt1.a* reads; WGS reads of *rtt1.a* and *RTT1.B* alleles mapped against references Bowman, Bonus, Foma, Kristina, and Montcalm. The vertical red line at the base 44954483 denotes the position of non-synonymous SNP in *COM2* underlying the *rtt1.a* mutant phenotype.


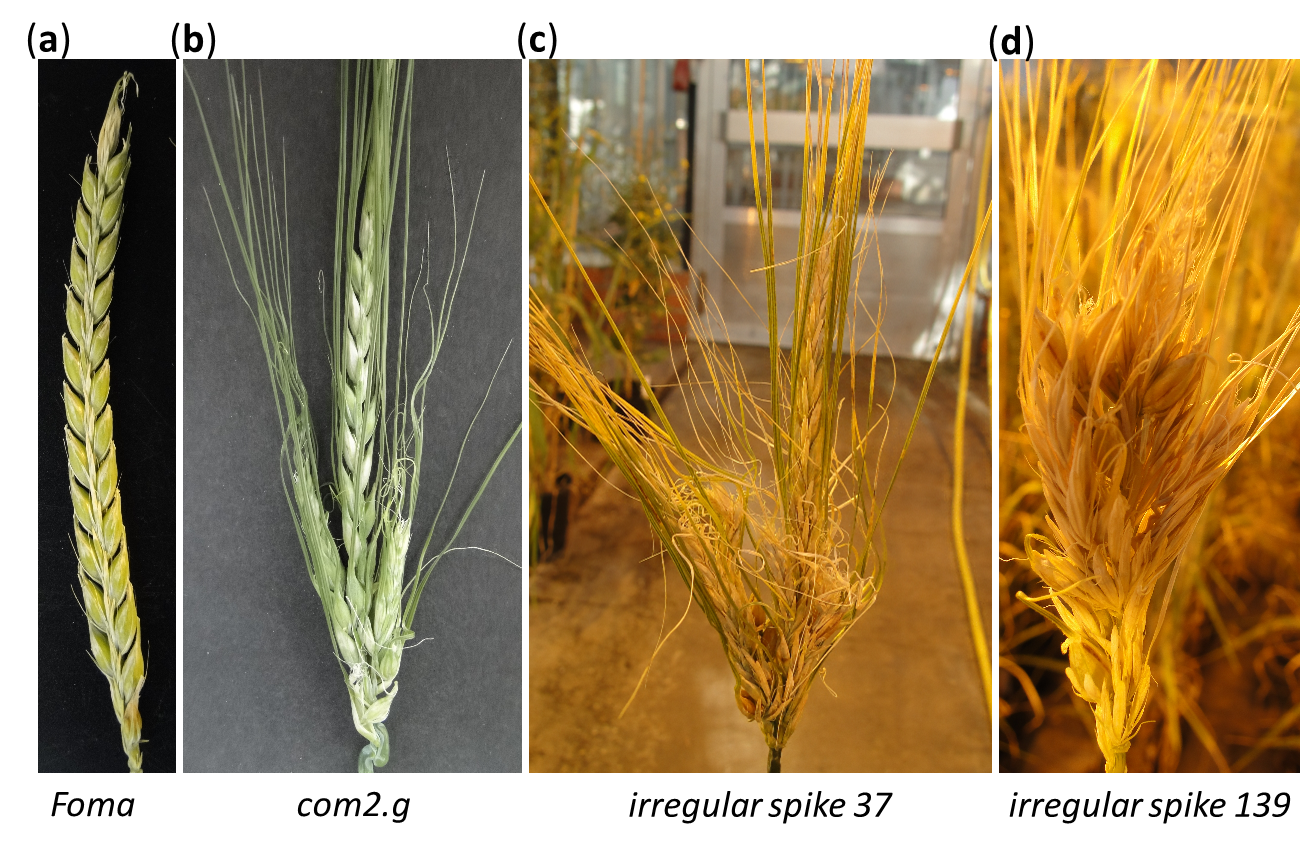


**Figure S3** Spike phenotypes of new *compositum 2* mutant alleles; (a-d) Mature spike phenotypes of unbranched wild-type Foma (a), and branched spikes of *compositum2.g* (b), *irregular spike 37* (c), and *irregular spike 139* (d) mutants.


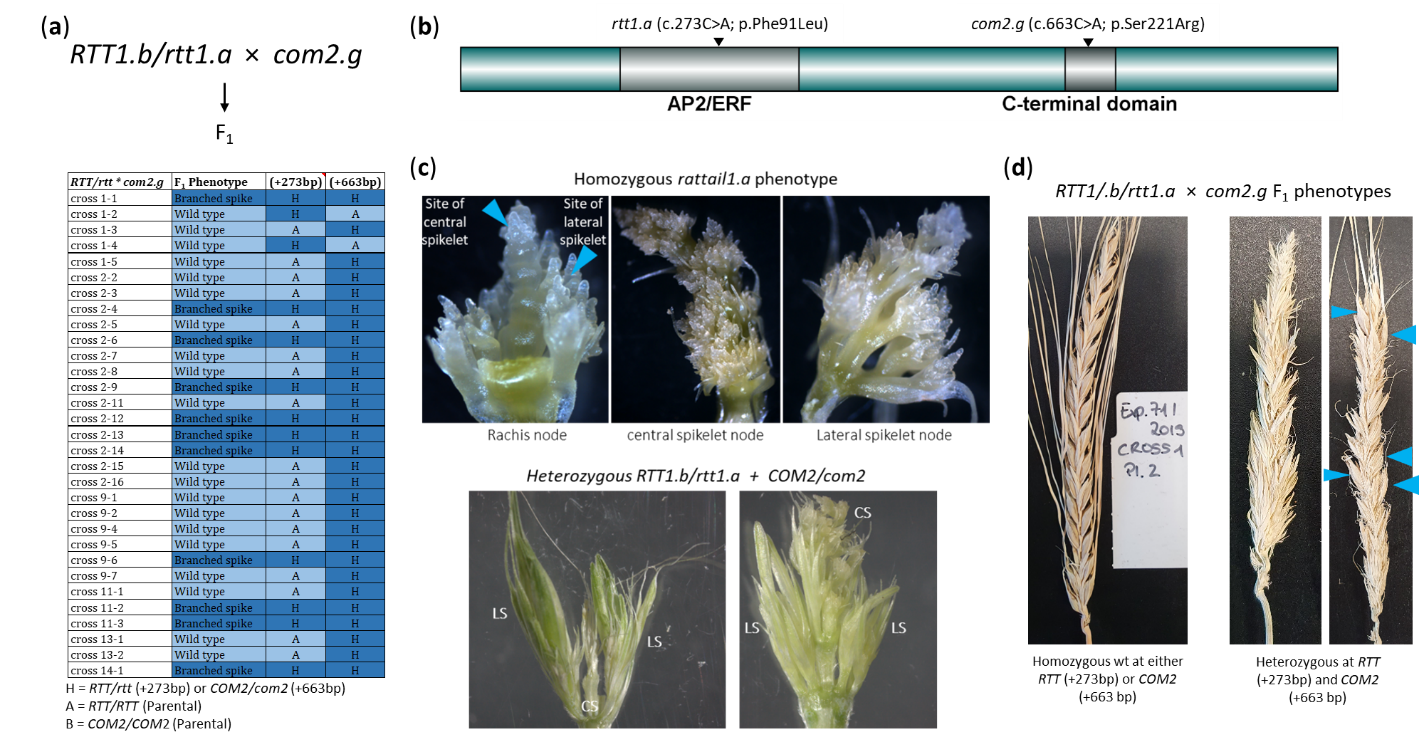


**Figure S4** Allelism analysis of *rattail 1.a* and *compositum 2.g*; (a) F_1_ analysis of the *RTT1.B/rtt1.a* × *com2.g* cross. (b) *COM2* gene model annotated with *rtt1.a* and *com2.g* diagnostic SNPs that were used for screening the F_1s_. (c) central and lateral spike phenotypes of homozygous *rtt1.a* (top) in comparison to that of F_1s_ carrying heterozygous *RTT/rtt* and *COM2/com2* alleles. (d) Mature spikes of F_1s_ with homozygous wild-type alleles at either *RTT1* or *COM2* in comparison to that of F_1s_ carrying heterozygous alleles at both loci.


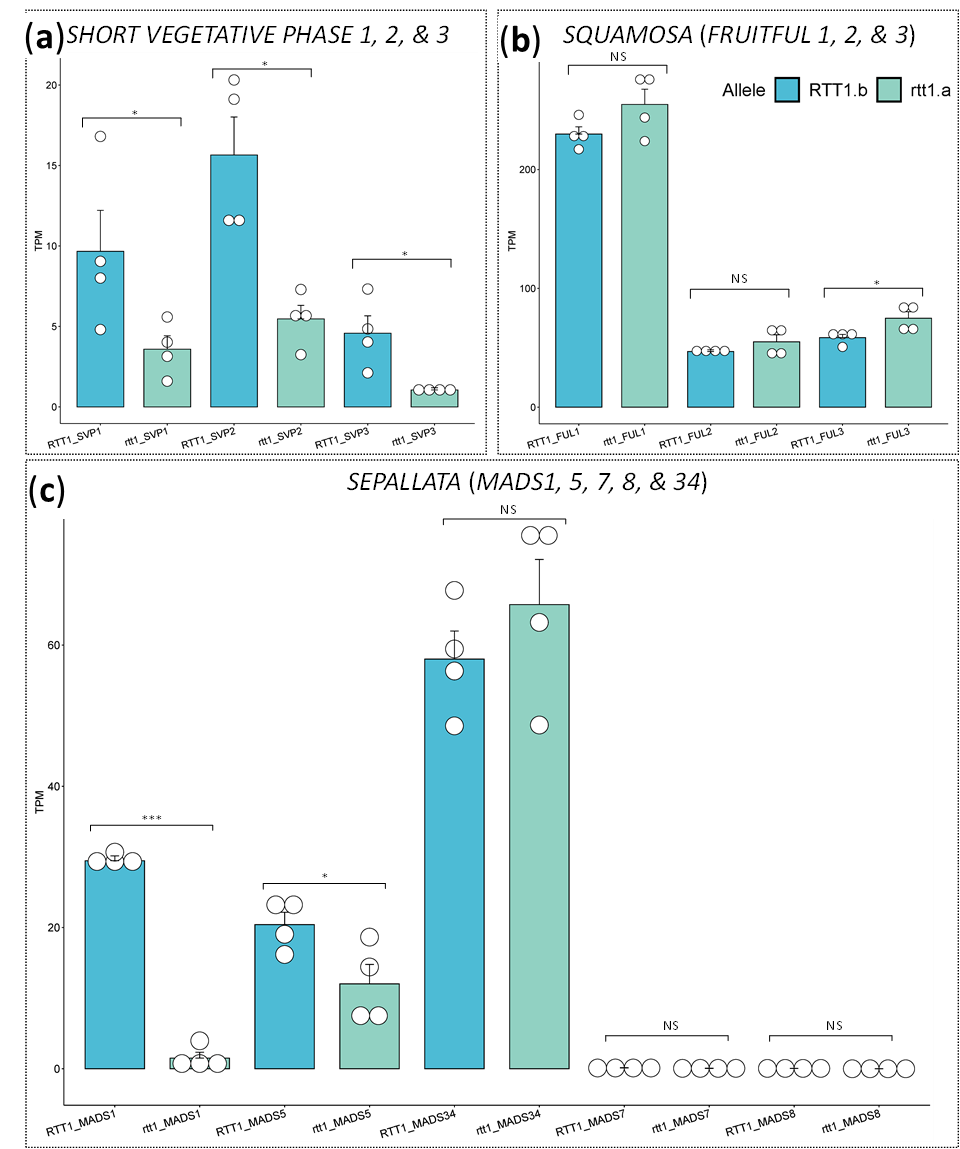


**Figure S5** Expression patterns of *SHORT VEGETATIVE PHASE, SQUAMOSA*, and *SEPALLATA* *MADS* box genes in *RTT1.b* and *rtt1.a*; (a) Down regulated expression of of *SVP1*, *SVP2* and *SVP3* in *rtt1.a.* (b) Higher expression of *FRUITFUL 3* (*FUL3*) and slightly increased expression of *FUL1* and *FUL2* (not significant) in *rtt1.a*. (c) The *SEPALLATA MADS* box genes, *MADS1* and *MADS5* were down regulated in *rtt1.a*, expression of *MADS34* remained unchanged, while the *MADS7* and *MADS8* were not expressed in the tissue sample. *p*-values ≤0,05: *; ≤0,01: **, ≤0,001: ***, NS not significant.


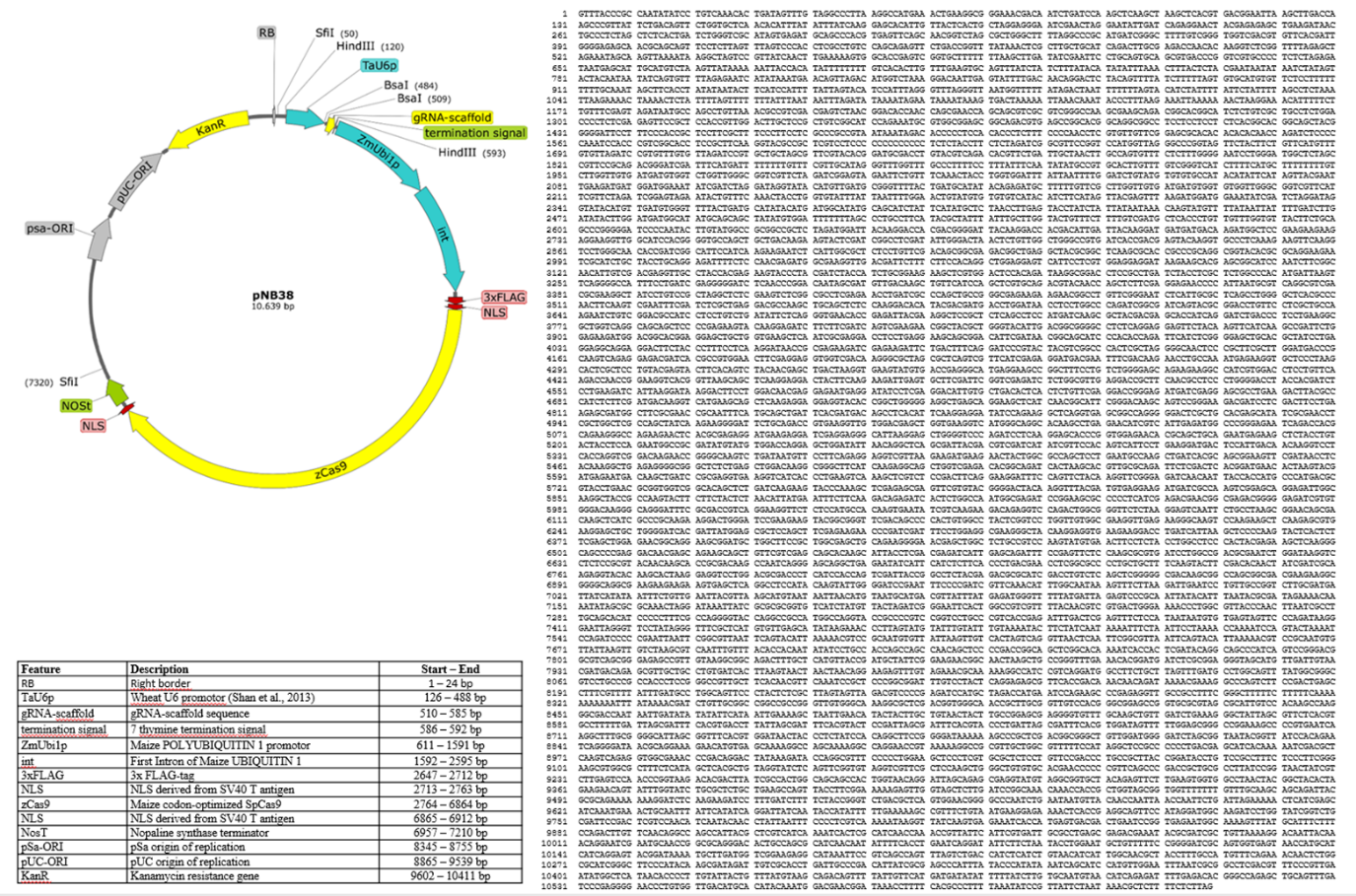


**Figure S6** Generic cloning vector pNB38 (GenBank-ID OR479081); plasmid map, feature descriptions and sequence.


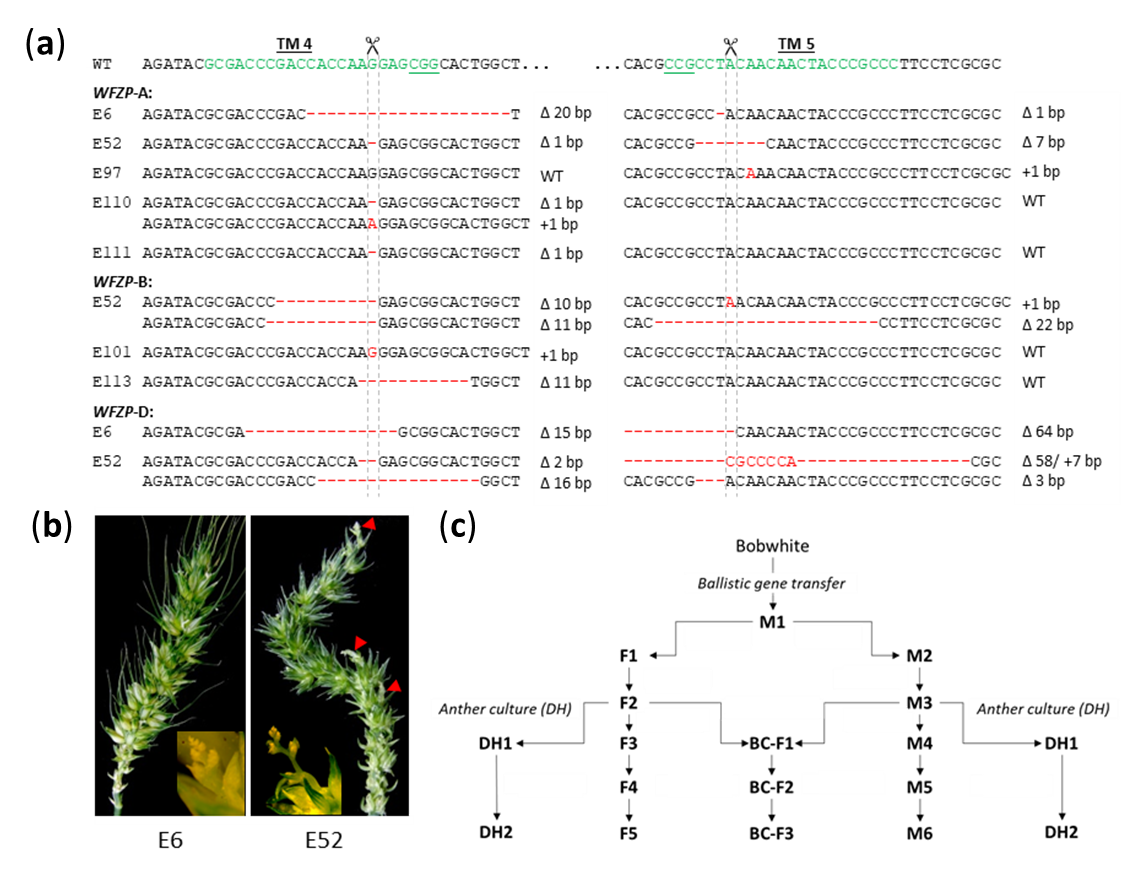


**Figure S7** Genotypic analyses and documentation of *WFZP* primary mutants; (a) wild-type (WT) and mutated alleles of regenerated plants carrying mutations in target motifs (TM) *WFZP*-TM4 and/or TM5, grey dashed lines: cutting positions of Cas9, red: mutation, green: TM with underlined PAM; (b) Spike and spikelet phenotypes of multi-mutated regenerants E6 and E52, red arrows mark branches; (c) schematic illustration of the generation of progeny from regenerated plants, M1-6: mutated regenerants (M1) and their progeny (M2-6) obtained by self-pollination, F1-5: filial generation of primary crosses (F1) and their progeny obtained by self-pollination (F2-F5), BC-F1-3: filial generation of backcrosses (BC-F1) and their progeny obtained by self-pollination (BC-F2/F3), DH1-2: generated doubled haploids (DH1) and their progeny obtained by self-pollination (DH2).


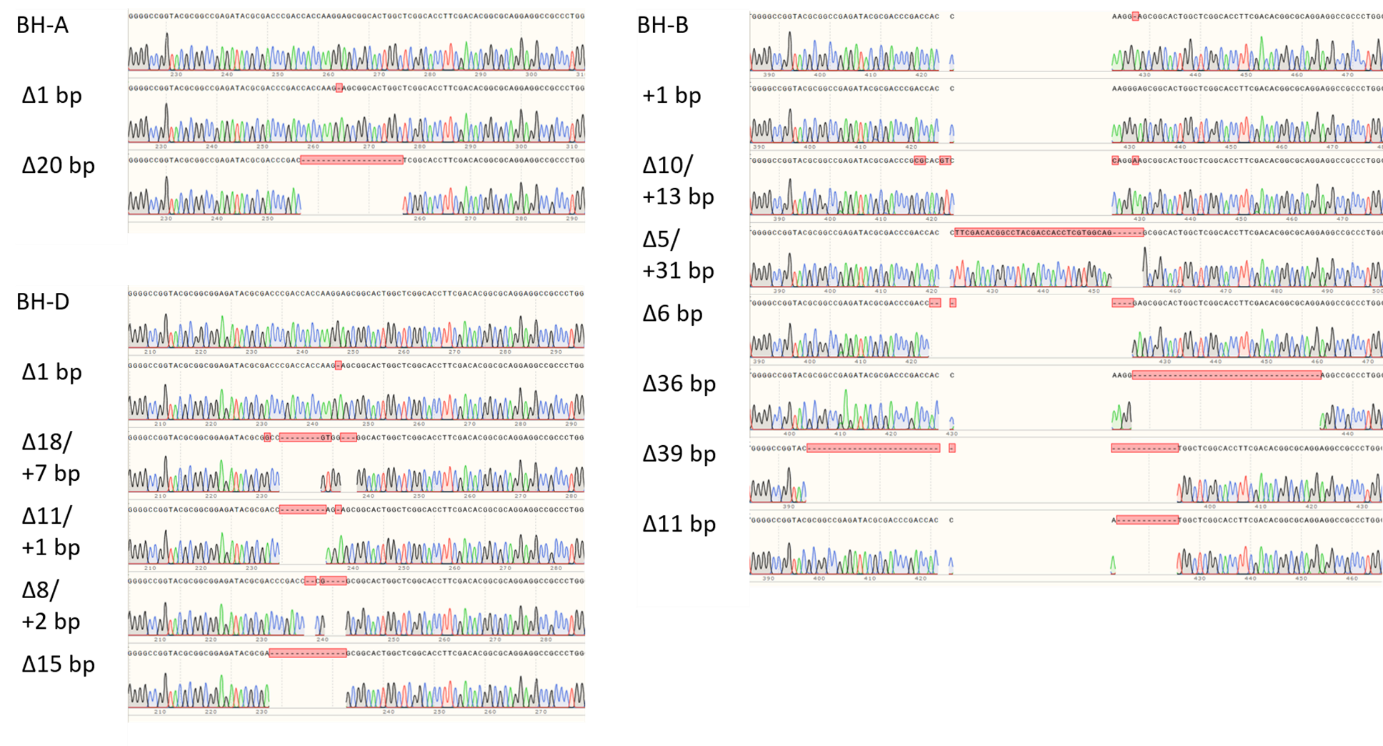


**Figure S8** Sanger chromatograms of all mutated and inherited *WFZP* mutant alleles.


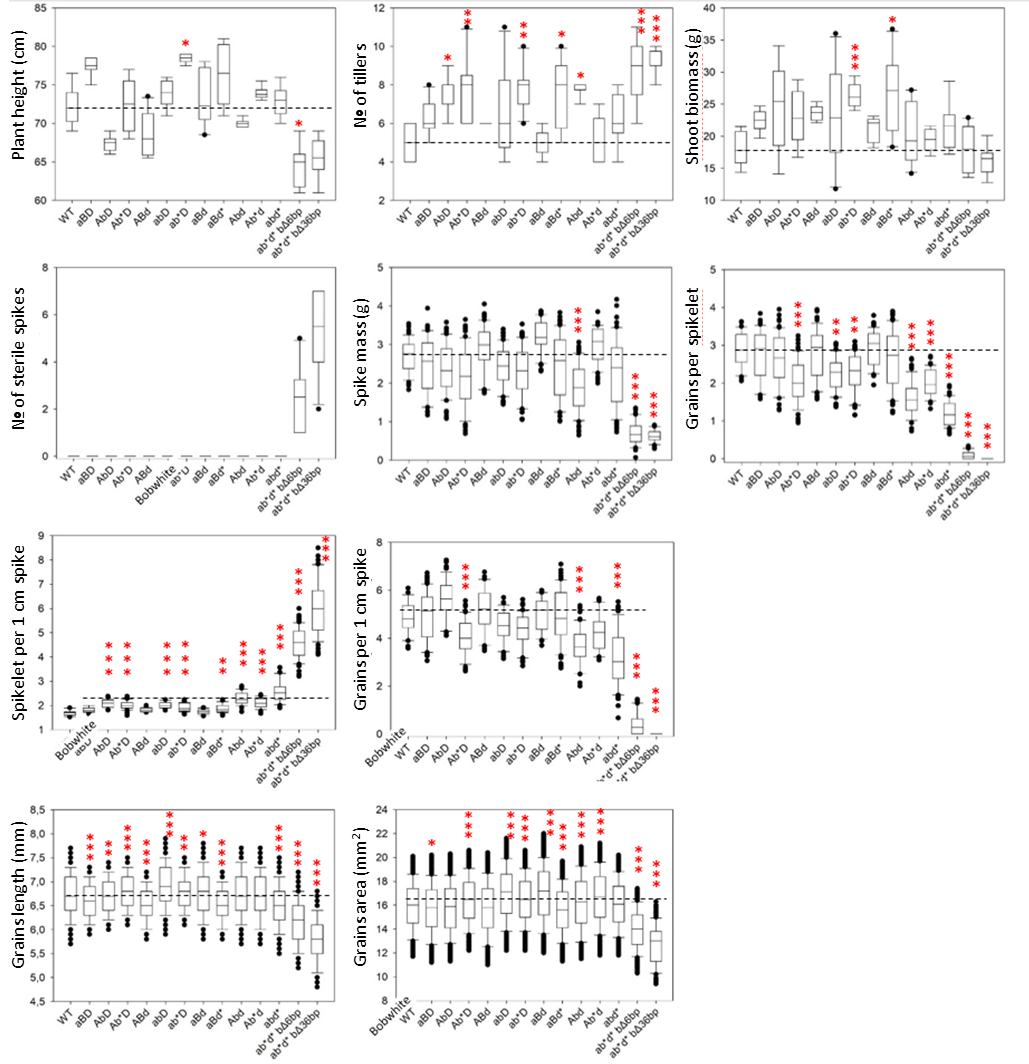


**Figure S9** Measured and normalized parameters of plant phenotyping of *WFZP* mutants growing under field-like conditions; black asterisks represent mutations without shift of reading frame; red asterisks represent significant difference level compared to wild-type Bobwhite, *p*-values ≤0,05: *; ≤0,01: **, ≤0,001: ***


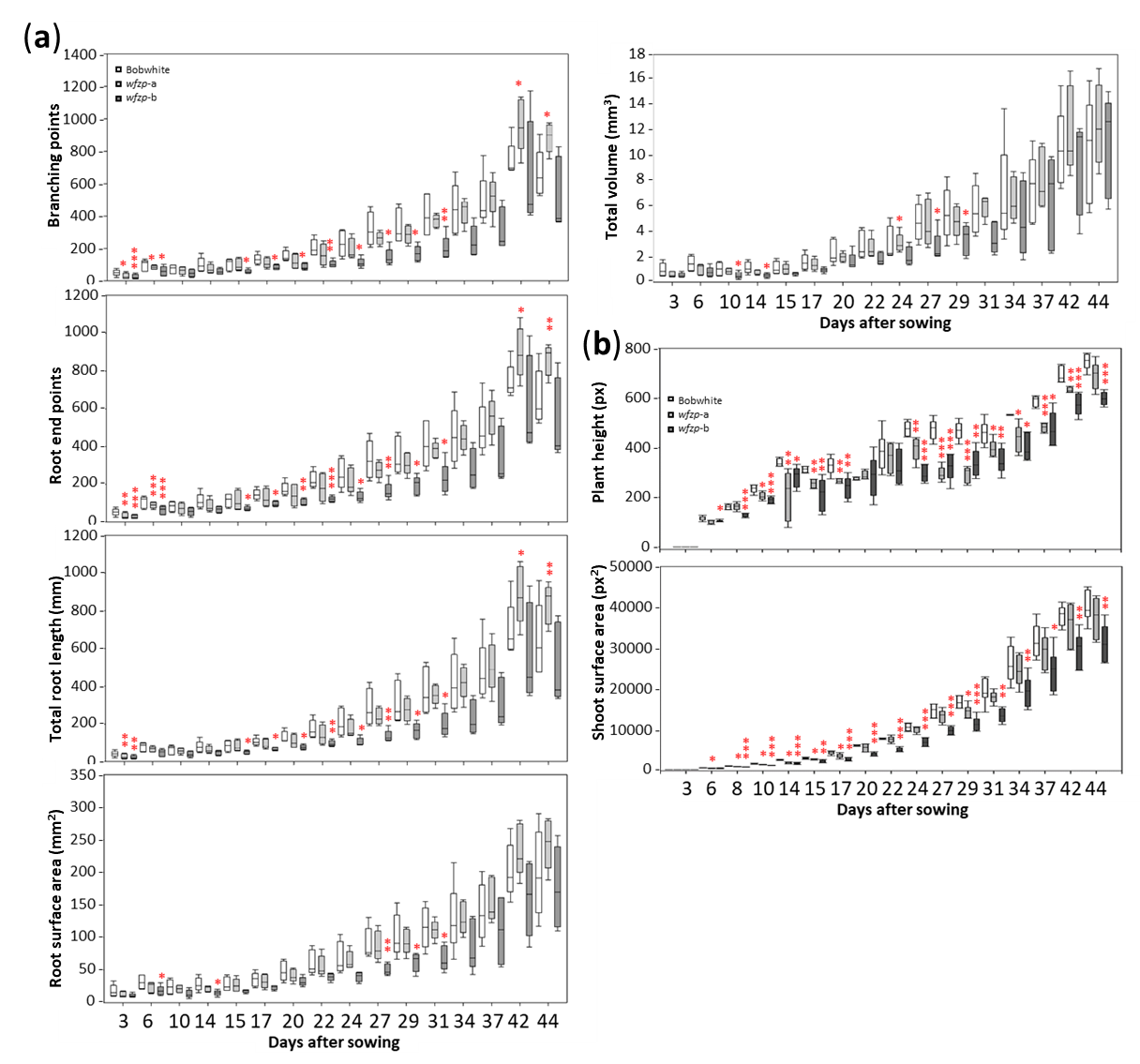


**Figure S10** Automatic phenotyping of root and shoot growth of *WFZP* mutants; (a) Root phenotyping parameters: branching points, end points, length, surface and volume; (b) Shoot phenotyping parameters: plant height and surface; red asterisks represent significant difference level compared to wild-type Bobwhite, *p*-values ≤0,05: *; ≤0,01: **, ≤0,001: ***


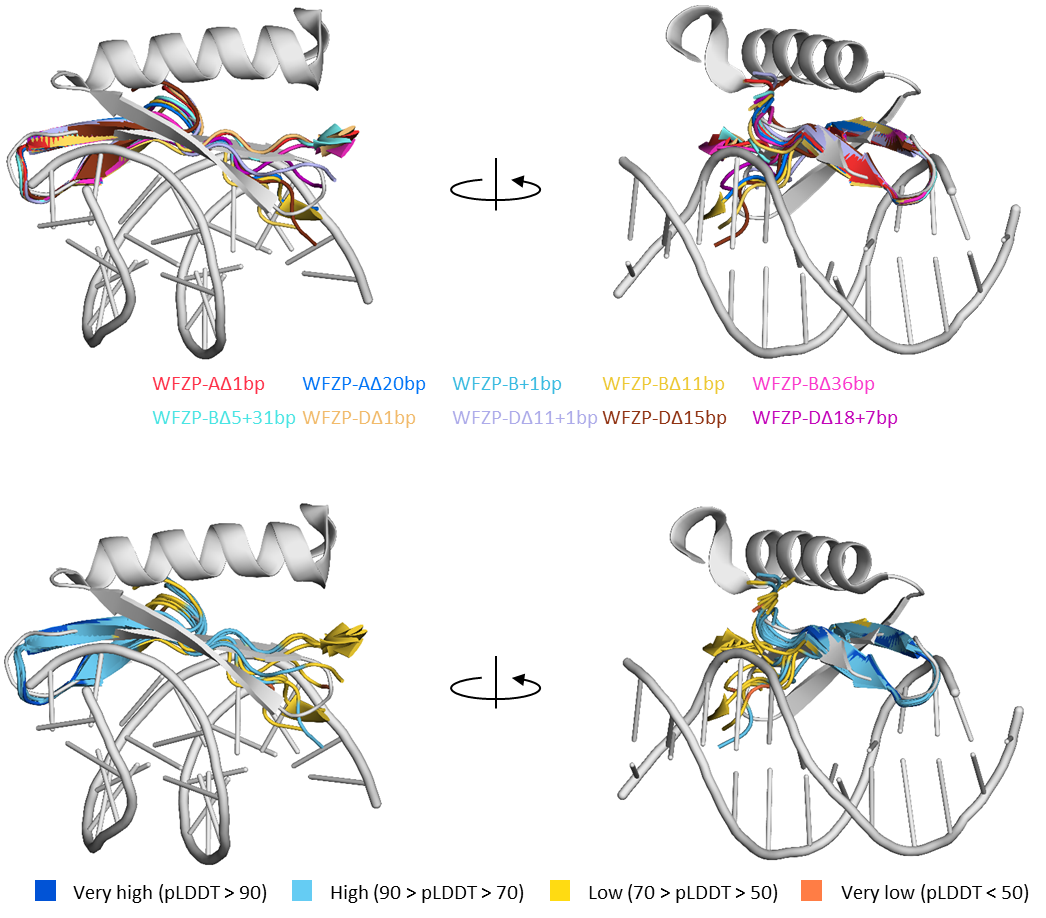


**Figure S11** Disrupted AP2/ERF domains; all predicted COM2 and WFZP protein structures with disrupted AP2/ERF domain (top: colored by mutant; bottom: colored by AlphaFold2's confidence score) are superposed to the homolog of *Arabidopsis* solved by crystallography (colored in grey) (PDBID 7et4), except WFZP-BΔ39bp which does not present any β-sheet stretch; same structures are displayed under two perspectives.


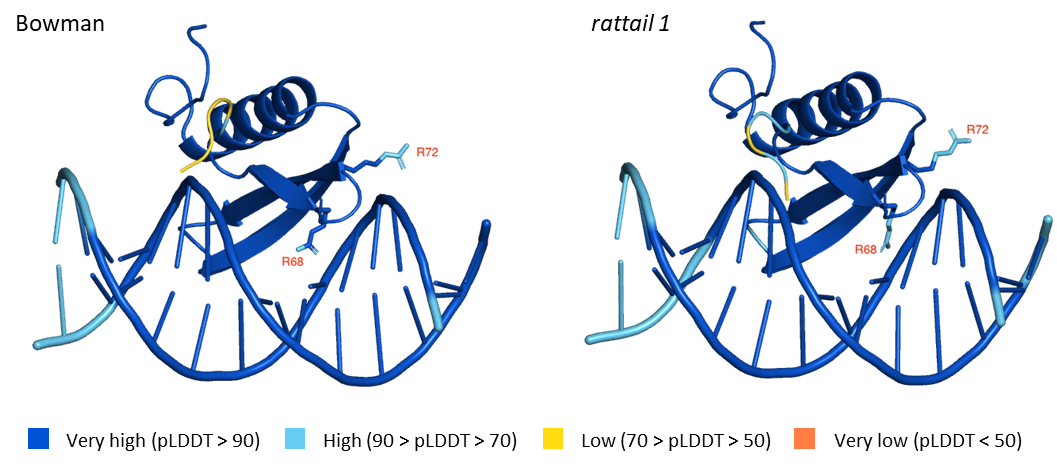


**Figure S12** Bowman and *rattail 1* COM2 AP2/ERF domains predicted in the presence of DNA with AlphaFold3; Alphafold3 predicted both the AP2/ERF domains of wild-type Bowman (left) and *rattail 1* (right) to be in similar conformation, especially regarding R68 and R72 interactions with the DNA; predicted structures colored by AlphaFold3 predicted local distance difference test (pLDDT) confidence score.


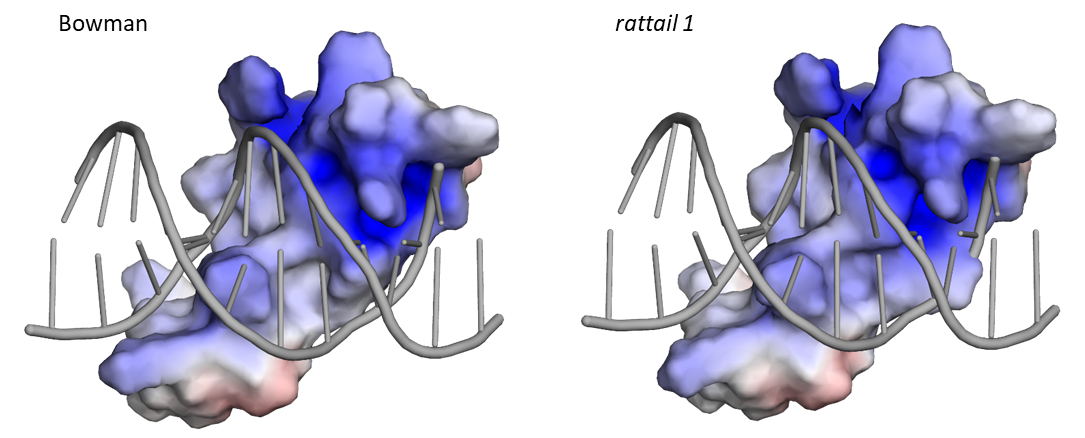


**Figure S13** Electrostatic surface distribution of COM2 AP2/ERF domains of Bowman and *rattail 1*; APBS calculated electrostatic surface distribution of the COM2 AP2/ERF domains of wild-type Bowman (left) and *rattail 1* (right) at the interface with the DNA; DNA was added by the superposition of each predicted structure with the crystallographically-solved homolog of *Arabidopsis* (PDBID 7et4); subtle differences in the shades of blue can be observed.


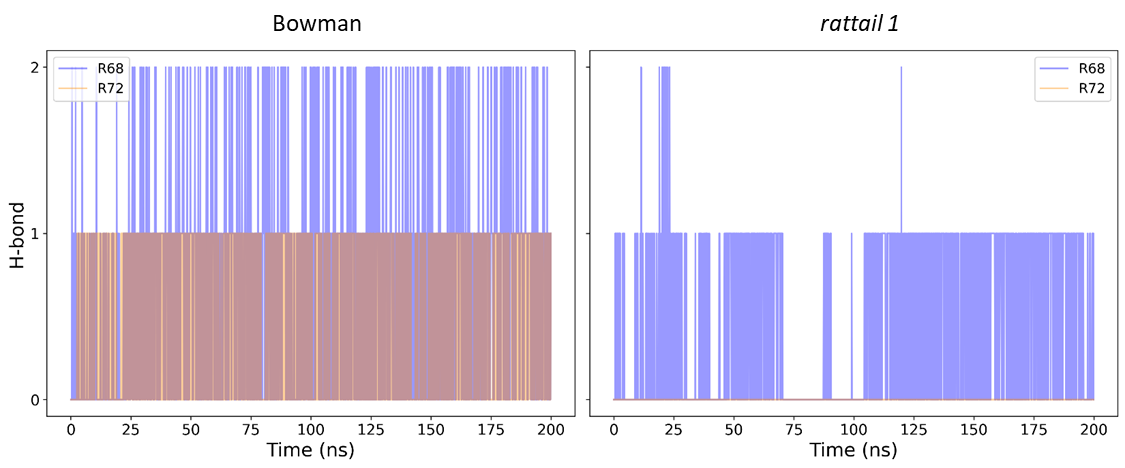


**Figure S14** Arginine hydrogen bonds during molecular dynamics simulation; Hydrogen bonds between R68 (blue) and R72 (orange) and the DNA are shown for both the COM2 AP2/ERF domains of wild-type Bowman (left) and *rattail 1* (right); bonds include interactions with both the DNA bases and phosphate groups.


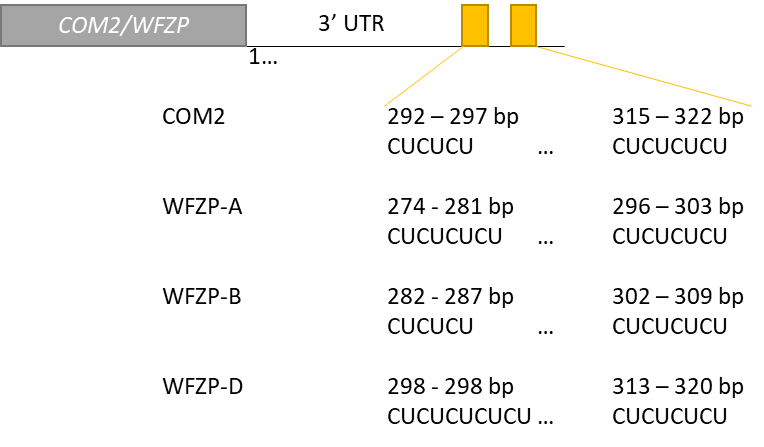


**Figure S15** CU sequence repeats in 3’ UTR of *COM2 and WFZP*; yellow boxes represent the two in 3’ untranslated region (3’ UTR) located CU sequence repeats; base pair coordinates calculated from start of 3’ UTR.


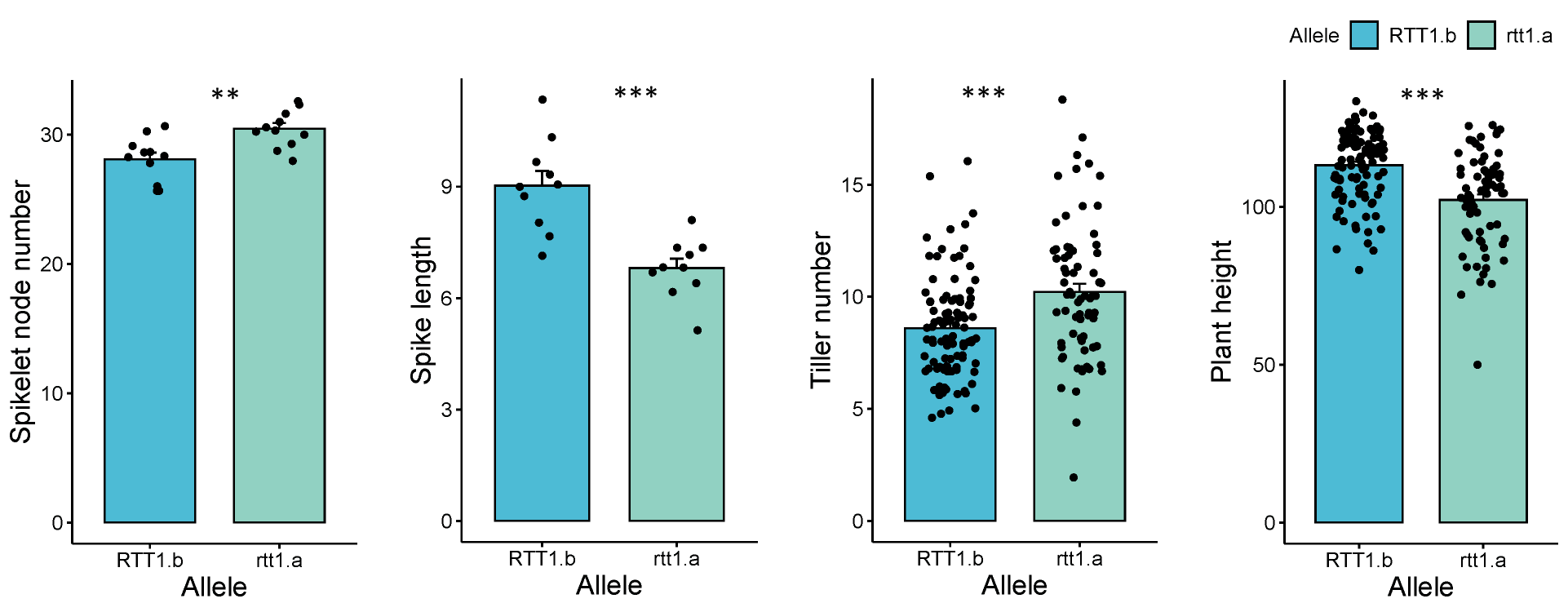


**Figure S16** Spike and plant architecture related traits in *RTT1.b* and *rtt1.a*; Differences between *RTT1.b* and *rtt1.a* for number of spikelet bearing nodes/spike (a), spike length (b), tiller numbers (c), and plant height (d). *p*-values ≤0,05: *, ≤0,01: **, ≤0,001: ***


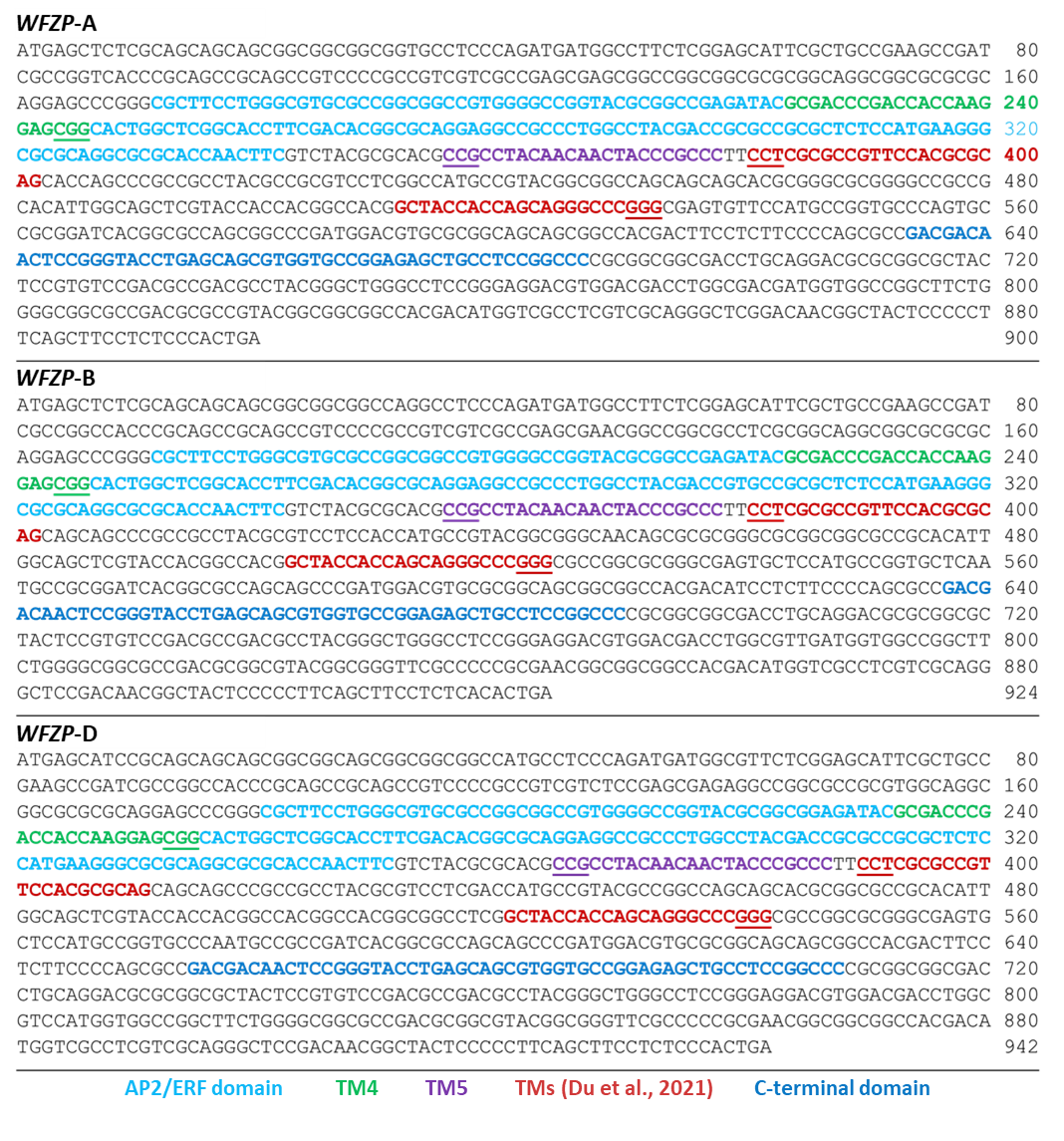


**Figure S17** Coding sequences of *WFZP* homoeoalleles; light blue: AP2/ERF domain; dark blue: C-terminal domain; target motifs TM4 (green), TM5 (violet), TMs from Du et al., 2021 (red); PAM underlined.


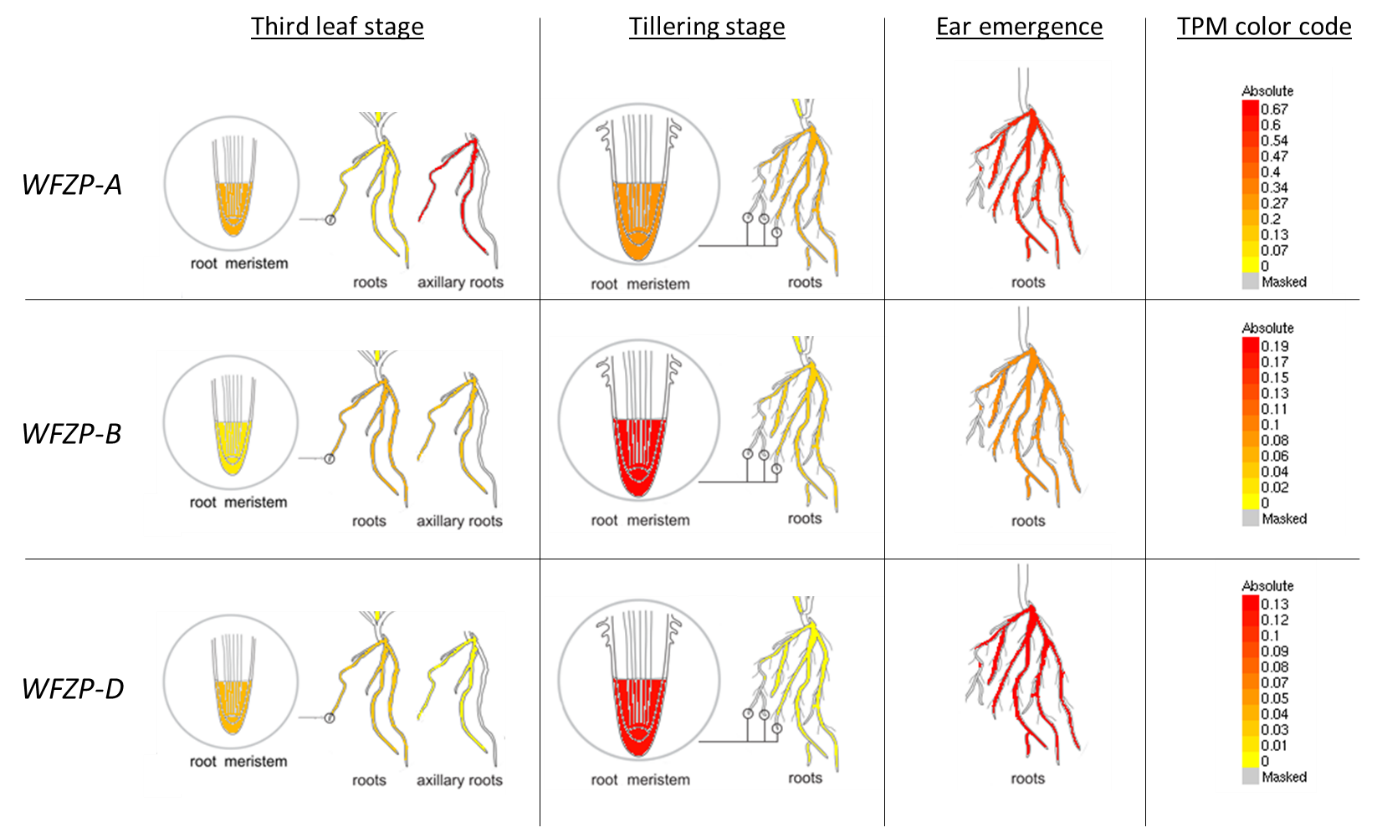


**Figure S18** Root expression of *WFZP* homoeoalleles; colors from yellow to red present transcript per million (TPM) values, allele-specific scales are shown; graphs and data are taken from Wheat eFP Browser (<http://bar.utoronto.ca/efp_wheat/cgi-bin/efpWeb.cgi>; Ramirez-Gonzalez et al., 2018).

1. *Supporting tables*

**Table S1** Germplasm screened for rtt1 segregation analysis; Gen: Generation, ind: individuum

| ***rtt1*_IPK_Gen 1** | **Spike phenotype_IPK_Gen 1** | **Progeny screen_IPK_Gen 2** | **Spike phenotype_IPK_Gen 2** | ***rtt1* population** |
| --- | --- | --- | --- | --- |
| *rtt1.a_Ind.1* | Unbranched spike | 17 individuals | No segregation | No |
| *rtt1.a_Ind.2* | Unbranched spike | 24 individuals | No segregation | No |
| *rtt1.a_Ind.3* | Unbranched spike | 22 individuals | No segregation | No |
| *rtt1.a_Ind.4* | Unbranched spike | 27 individuals | No segregation | No |
| *rtt1.a_Ind.5* | Unbranched spike | 17 individuals | No segregation | No |
| *rtt1.a_Ind.6* | Unbranched spike | 29 individuals | 18 unbranched + 11 branched | Yes; *rtt1_pop1* |
| *rtt1.a_Ind.7* | Unbranched spike | 17 individuals | No segregation | No |
| *rtt1.a_Ind.8* | Unbranched spike | 19 individuals | No segregation | No |
| *rtt1.a_Ind.9* | Unbranched spike | 24 individuals | No segregation | No |
| *rtt1.a_Ind.10* | Unbranched spike | 24 individuals | 20 unbranched + 4 branched | Yes; *rtt1_pop2* |
| *rtt1.a_Ind.11* | *Branched spike (rtt1.a)* | - | - | - |
| *rtt1.a_Ind.12* | *Branched spike* | - | - | - |
| *rtt1.a_Ind.13* | Unbranched spike | 16 individuals | No segregation | No |
| *rtt1.a_Ind.14* | Unbranched spike | 17 individuals | No segregation | No |
| *rtt1.a_Ind.15* | Unbranched spike | 14 individuals | No segregation | No |
| *rtt1.a_Ind.16* | Unbranched spike | 17 individuals | No segregation | No |
| *rtt1.a_Ind.17* | Unbranched spike | 18 individuals | No segregation | No |
| *rtt1.a_Ind.18* | Unbranched spike | 54 individuals | No segregation | No |
| *rtt1.a_Ind.19* | Unbranched spike | 54 individuals | 43 unbranched + 11 branched | Yes; *rtt1_pop3* |
| *rtt1.a_Ind.20* | Unbranched spike | 54 individuals | No segregation | No |
| *rtt1.a_Ind.21* | Unbranched spike | 54 individuals | No segregation | No |
| *rtt1.a_Ind.22* | Unbranched spike | 54 individuals | No segregation | No |
| *rtt1.a_Ind.23* | Unbranched spike | 54 individuals | No segregation | No |
| *rtt1.a_Ind.24* | Unbranched spike | 54 individuals | No segregation | No |
| *rtt1.a_Ind.25* | Unbranched spike | 54 individuals | No segregation | No |
| *rtt1.a_Ind.26* | *Branched spike (rtt1.a)* | - | - | - |
| *rtt1.a_Ind.27* | *Branched spike (rtt1.a)* | - | - | - |
| *rtt1.a_Ind.28* | Unbranched spike | 54 individuals | No segregation | No |
| *rtt1.a_Ind.29* | Unbranched spike | 54 individuals | No segregation | No |
| *rtt1.a_Ind.30* | Unbranched spike | 54 individuals | No segregation | No |
| *rtt1.a_Ind.31* | Unbranched spike | 54 individuals | No segregation | No |
| *rtt1.a_Ind.32* | Unbranched spike | 54 individuals | No segregation | No |
| *rtt1.a_Ind.33* | Unbranched spike | 54 individuals | No segregation | No |
| *rtt1.a_Ind.34* | Unbranched spike | 54 individuals | No segregation | No |
| *rtt1.a_Ind.35* | Unbranched spike | 54 individuals | 33 unbranched + 21 branched | Yes; *rtt1_pop4* |
| *rtt1.a_Ind.36* | Unbranched spike | 54 individuals | No segregation | No |
| *rtt1.a_Ind.37* | Unbranched spike | 54 individuals | No segregation | No |
| *rtt1.a_Ind.38* | Unbranched spike | 54 individuals | No segregation | No |
| *rtt1.a_Ind.39* | Unbranched spike | 54 individuals | No segregation | No |
| *rtt1.a_Ind.40* | Unbranched spike | 54 individuals | No segregation | No |
| *rtt1.a_Ind.41* | Unbranched spike | 54 individuals | No segregation | No |
| *rtt1.a_Ind.42* | Unbranched spike | 54 individuals | No segregation | No |
| *rtt1.a_Ind.43* | Unbranched spike | 99 individuals | 72 unbranched + 27 branched | Yes; *rtt1_pop5* |
| *rtt1.a_Ind.44* | Unbranched spike | 54 individuals | No segregation | No |
| *rtt1.a_Ind.45* | Unbranched spike | 54 individuals | No segregation | No |
| *rtt1.a_Ind.46* | Unbranched spike | 54 individuals | No segregation | No |
| *rtt1.a_Ind.47* | Unbranched spike | 54 individuals | No segregation | No |
| *rtt1.a_Ind.48* | Unbranched spike | 54 individuals | No segregation | No |
| *rtt1.a_Ind.49* | Unbranched spike | 54 individuals | No segregation | No |
| *rtt1.a_Ind.50* | Unbranched spike | 54 individuals | No segregation | No |
| *rtt1.a_Ind.51* | Unbranched spike | 54 individuals | No segregation | No |
| *rtt1.a_Ind.52* | Unbranched spike | 54 individuals | No segregation | No |
| *rtt1.a_Ind.53* | *Branched spike (rtt1.a)* | - | - | - |
| *rtt1.a_Ind.54* | Unbranched spike | 54 individuals | No segregation | No |
| *rtt1.a_Ind.55* | Unbranched spike | 54 individuals | No segregation | No |
| *rtt1.a_Ind.56* | Unbranched spike | 54 individuals | No segregation | No |
| *rtt1.a_Ind.57* | Unbranched spike | 54 individuals | No segregation | No |

**Table S2** Oligonucleotides and vectors used

| *Oligonucleotides* |  |  |
| --- | --- | --- |
| **Name** | **Sequence** (overhangs underlined) | **Reference** |
| *Genetic marker for rtt1* |  |  |
| rtt1_CAPS_F | ATGATGGCGTTCTCGGAGCATTCG | current study |
| rtt1_CAPS_R | GCGCGTAGACGAAGTTGGTGC | current study |
| *Cloning of specific gRNA unit* | | |
| TM4-F | CTTGCGACCCGACCACCAAGGAG | current study |
| TM4-R | AAACCTCCTTGGTGGTCGGGTCG | current study |
| TM5-F | CTTGGGCGGGTAGTTGTTGTAGG | current study |
| TM5-R | AAACCCTACAACAACTACCCGCC | current study |
| *Vector sequencing* |  |  |
| pSH91_S1 | AGGCCATGAAACTGAAGG | current study |
| CH159-p6i-RB | GAGTTCCCGCCACAGACC | current study |
| CH160-p6i-E9 | TGTCAAATCGTGGCCTCTAA | current study |
| *Amplification of target regions* | | |
| CH24-WFZP-A-F | GGCGGGAGCAGTAGTATAG | current study |
| CH26-WFZP-A-R | CCGCCACTTTAATTCCACAC | current study |
| CH29-WFZP-B-F | AGTGCTCTCAGCCTCTCACTC | current study |
| CH31-WFZP-B-R | TGCCGGTGCATTTGCTTCAG | current study |
| CH5-WFZP-D-F | GCCTCACTTCACTTCAGTTC | current study |
| CH6-WFZP-D-R | CCACGTACTGTGACTGATCG | current study |
| *T-DNA screening* |  |  |
| TaU6-F1 | GCCCGTTATTCTGACAGTTC | current study |
| Bie475 | TTTAGCCCTGCCTTCATACG | Gerasimova et al., 2020 |
| zCas9-R1 | TTAATCATGTGGGCCAGAGC | Gerasimova et al., 2020 |
| 35S-F2 | CATGGTGGAGCACGACACTCTC | Gerasimova et al., 2020 |
| Hyg-R5 | GATTCCTTGCGGTCCGAATG | Gerasimova et al., 2020 |
| *Vectors* |  |  |
| **Name** | **Description** | **Reference** |
| pSH121 | Generic vector for genome editing with OsU3p | Gerasimova et al., 2020; GenBank-ID MW145140.1 |
| pNB38 | Generic vector for genome editing with TaU6p | current study; GenBank-ID OR479081 |
| p6i-d35S-TE9 | Generic binary vector | DNA Cloning Service, Hamburg, Germany) |
| pCH26_WFZP-TM4-int | Intermediate vector for *WFZP* genome editing in TM4 | current study |
| pCH27_WFZP-TM5-int | Intermediate vector for *WFZP* genome editing in TM5 | current study |
| pCH28_WFZP-TM4 | Binary vector for *WFZP* genome editing in TM4 | current study |
| pCH29_WFZP-TM5 | Binary vector for *WFZP* genome editing in TM5 | current study |

**Table S3** SNP and deletion mapping in rtt1.a and RTT1.b based on WGS.

(see extra file)

**Table S4** irregular spike mutants with spike branching screened for identifying mutations in COM2; NGB - Nordic Genebank - NordGen; GSHO - barley genotype ID from National Small Grains Collection, Aberdeen Idaho

| **Mutant name** | **NGB/GSHO number** | **Progenitor** | **Mutagen** | **Spike phenotype** | ***COM2 cds position 273*** | ***COM2 cds position 280*** | ***COM2 cds position 667*** |
| --- | --- | --- | --- | --- | --- | --- | --- |
| *irregular spike 2* | NGB113452 | BONUS | fast neutrons | Branched spike | C | G | G |
| *irregular spike 3* | NGB113453 | BONUS | X-rays | Branched spike | C | G | G |
| *irregular spike 5* | NGB113455 | BONUS | ethylene oxide | Branched spike | C | G | G |
| *irregular spike 12* | NGB113462 | FOMA | spontaneous | Branched spike | C | G | G |
| *irregular spike 19* | NGB113469 | FOMA | neutrons | Branched spike | C | G | G |
| *irregular spike 29* | NGB113479 | FOMA | ethyl methanesulfonate | Branched spike | C | G | G |
| *irregular spike 30* | NGB113480 | FOMA | ethylene imine | Branched spike | C | G | G |
| *irregular spike 37* | NGB113487 | FOMA | ethyl methanesulfonate | Branched spike | C | A | G |
| *irregular spike 41* | NGB113491 | FOMA | neutrons + neutrons | Branched spike | C | G | G |
| *irregular spike 103* | NGB113552 | FOMA | ethyl methanesulfonate | Branched spike | C | G | G |
| *irregular spike 139* | NGB113594 | KRISTINA | ethyl methanesulfonate | Branched spike | C | G | A |
| *irregular spike 149* | NGB113604 | BONUS | sodium azide | Branched spike | C | G | G |
| *irregular spike 151* | NGB113606 | BONUS | ethyl methanesulfonate | Branched spike | C | G | G |
| *irregular spike 94* | NGB113543 | FOMA | ethyl methanesulfonate | Branched spike | C | G | G |
| *irregular spike 95* | NGB113544 | FOMA | ethyl methanesulfonate | Branched spike | C | G | G |
| *rattail 1.a* | GSHO 216 | Goldfoil | spontaneous | Branched spike | A | G | G |

**Table S5** Differentially expressed genes in the rtt1 mutant.

(see extra file)

**Table S6** FIMO motif scanning data in rtt1-dependently up- and down-regulated genes.

(see extra file)

**Table S7** XSTREME motif enrichment data in rtt1-dependently up- and down-regulated genes.

(see extra file)

**Table S8** **Off-target analyses in wheat**

| **target motif** | **BLAST Hit** | **Sequence** |
| --- | --- | --- |
| **TM4** | *TraesCS2A02G116900 (WFZP-A1)* | GCGACCCGACCACCAAGGAGCGG |
|  | *TraesCS2B02G136100 (WFZP-B1)* |  |
|  | *TraesCS2D02G118200 (WFZP-D1)* |  |
|  | Chr. 1D (OFF-Target Region 1) | ---ACCCGACCACCAAGGAGC-- |
|  | Chr. 4D (OFF-Target Region 2) | ------CGACCACCAAGGAGCGG |
|  | Chr. 3A (OFF-Target Region 3) | -CGACCCGACCACCAAGG----- |
| **TM5** | *TraesCS2A02G116900 (WFZP-A1)* | GGGCGGGTAGTTGTTGTAGGCGG |
|  | *TraesCS2B02G136100 (WFZP-B1)* |  |
|  | *TraesCS2D02G118200 (WFZP-D1)* |  |
|  | Chr. 2D (OFF-Target Region 4) | ------GTAGTTGTTGTAGGCGG |

**Table S9** Genotyping of primary WFZP mutants and their progeny; green and *: in frame mutation; red: no grains; wt: wild-type allele; M1-6: Mutated generation 1 to 6; F1-5: filial generation 1 to 5 of primary crosses; BC-F1-3: filial generation 1 to 3 of backcrosses; DH: doubled haploid regenerants; het: heterozygous; TM: target motif; Δ: deletion

| **Generation** | **Plant ID** | **Allelic code** | ***WFZP*-A** | ***WFZP*-B** | ***WFZP*-D** | **T-DNA** |
| --- | --- | --- | --- | --- | --- | --- |
| *Wild-type control derived from M1 plants* | | | | | | |
|  | E108 | Bobwhite | *wt* | *wt* | *wt* |  |
| *Not-heritated M1 plants* | | | | | | |
|  | E52 | abd (chimeric) | *wt*  Δ1 (TM4)  Δ7 (TM5) | *wt*  +1 (TM4)  Δ10 (TM4)  Δ11 (TM4)  Δ22 (TM5) | *wt*  Δ2 (TM4)  Δ16 (TM4)  Δ3 (TM5)  Δ9/+6 (TM5)  Δ58/+7 (TM5) | gRNA4, gRNA5, *cas9, hpt* |
|  | E97 |  | *wt* / +1 | *wt* | *wt* | gRNA4 |
|  | E110 |  | *wt*/+1/Δ1 | *wt* | *wt* |  |
| *Plant family 6* | | | | | | |
| **M1** | E6 |  | *wt*  Δ20 (TM4)  Δ1 (TM5) | *wt* | *wt*  Δ15 (TM4)  Δ64 (TM5) | gRNA4, gRNA5, *cas9, hpt* |
| **>M2** | E6-3 |  | Δ20 | *wt* (het) | Δ15 | gRNA4, gRNA5, *cas9, hpt* |
| **>>M3** | E6-3-2 |  | Δ20 | Δ6 | Δ15 | gRNA4, gRNA5, *cas9, hpt* |
| **>>>M4** | E6-3-2-2 |  | Δ20 | Δ6 | Δ15 | gRNA4, *cas9, hpt* |
| **>>>>M5** | E6-3-2-2-1 |  | Δ20 | Δ6 | Δ15 | gRNA4, *cas9, hpt* |
| **>>>>>M6** | E6-3-2-2-1-1 | ab*d* bΔ6bp | Δ20 | Δ6 | Δ15 | gRNA4, *cas9, hpt* |
| **>>M3** | E6-3-4 |  | Δ20 | Δ36 | Δ15 | gRNA4, gRNA5, *cas9, hpt* |
| **>>>M4** | E6-3-4-5 |  | Δ20 | Δ36 | Δ15 | gRNA4, *cas9, hpt* |
| **>>>>M5** | E6-3-4-5-10 |  | Δ20 | Δ36 | Δ15 | gRNA4, *cas9, hpt* |
| **>>>>>M6** | E6-3-4-5-10-1 | ab*d* bΔ36bp | Δ20 | Δ36 | Δ15 | gRNA4, *cas9, hpt* |
| **>>M3** | E6-3-8 |  | Δ20 | Δ6 | Δ15 | gRNA4, gRNA5, *cas9, hpt* |
| *Plant family 101* | | | | | | |
| **M1** | E101 |  | *wt* | *wt* / +1 | *wt* |  |
| **>M2** | E101-1 |  | *wt* | +1 | *wt* |  |
| **>>M3** | E101-1-1 |  | *wt* | +1 | *wt* |  |
| **>>>M4** | E101-1-1-1 |  | *wt* | +1 | *wt* |  |
| **>>>>M5** | E101-1-1-1-2 |  | *wt* | +1 | *wt* |  |
| **>>>>>M6** | E101-1-1-1-2-1 | AbD | *wt* | +1 | *wt* |  |
| *Plant family 111* | | | | | | |
| **M1** | E111 |  | *wt* / Δ1 | *wt* | *wt* | gRNA4, *cas9, hpt* |
| **>M2** | E111-4 |  | *wt* (het) | *wt* | *wt* | gRNA4, *cas9, hpt* |
| **>>M3** | E111-4-2 |  | Δ1 | *wt* | *wt* | gRNA4, *cas9, hpt* |
| **>>>M4** | E111-4-2-1 |  | Δ1 | *wt* | *wt* |  |
| **>>>>M5** | E111-4-2-1-1 |  | Δ1 | *wt* | *wt* |  |
| **>>>>>M6** | E111-4-2-1-1-1 | aBD | Δ1 | *wt* | *wt* |  |
| **>>M3** | E111-4-8 |  | *wt* (het) | *wt* | *wt* (het) | gRNA4, *cas9, hpt* |
| **>>>DH** | E111-4-8-DH1 | ABd | *wt* | *wt* | Δ18/+7 |  |
|  | E111-4-8-DH3 | aBd | Δ1 | *wt* | Δ18/+7 |  |
| *Plant family 113* | | | | | | |
| **M1** | E113 |  | *wt* / Δ11 | *wt* | *wt* | gRNA4, *cas9, hpt* |
| **>M2** | E113-3 |  | *wt* (het) | *wt* (het) | *wt* | gRNA4, *cas9, hpt* |
| **>>M3** | E113-3-1 |  | *wt* | Δ39 | *wt* (het) | gRNA4, *cas9, hpt* |
| **>>>M4** | E113-3-1-2 |  | *wt* | Δ39 | Δ1 | gRNA4, *cas9, hpt* |
| **>>>>M5** | E113-3-1-2-2 |  | *wt* | Δ39 | Δ1 |  |
| **>>>>>M6** | E113-3-1-2-2-1 | Ab*d | *wt* | Δ39 | Δ1 |  |
| **>>M3** | E113-3-7 | AbD | *wt* | Δ11 | *wt* |  |
| *97x113 primary crossing filial family 3* | | | | | | |
| **F1** | 97x113-3 |  | *wt* | *wt* (het) | *wt* | gRNA4, *cas9, hpt* |
| **>F2** | 97x113-3-1 |  | *wt* | Δ10/+13 | *wt* | gRNA4, *cas9, hpt* |
| **>>F3** | 97x113-3-1-2 |  | *wt* | Δ10/+13 | *wt* | *cas9, hpt* |
| **>>>F4** | 97x113-3-1-2-3 |  | *wt* | Δ10/+13 | *wt* | *cas9* |
| **>>>>F5** | 97x113-3-1-2-3-1 | Ab*D | *wt* | Δ10/+13 | *wt* |  |
| *97x113 primary crossing filial family 4* | | | | | | |
| **F1** | 97x113-4 |  | *wt* (het) | *wt* | *wt* | gRNA4, *cas9, hpt* |
| **>F2** | 97x113-4-3 |  | *wt* (het) | *wt* / Δ24 | *wt* (het) | gRNA4, *cas9, hpt* |
| **>>F3** | 97x113-4-3-2 | abd | Δ1 | Δ1 / +26 | Δ1 | gRNA4, *cas9, hpt* |
| **>>F3** | 97x113-4-3-6 |  | *wt* (het) | Δ24 | *wt* (het) | gRNA4, *cas9, hpt* |
| **>>>F4** | 97x113-4-3-6-4 | ab*d | Δ1 | Δ24 | Δ32 | gRNA4, *cas9, hpt* |
| **>>>F4** | 97x113-4-3-6-5 | ab*d | Δ14 | Δ24 | Δ32 | gRNA4, *cas9, hpt* |
| **>>>F4** | 97x113-4-3-6-6 |  | Δ14 | Δ24 | *wt* (het) | gRNA4, *cas9, hpt* |
| **>>>>F5** | 97x113-4-3-6-6-9 | ab*d | Δ14 | Δ24 | +1 | gRNA4, *cas9, hpt* |
| **>>>F4** | 97x113-4-3-6-7 | ab*d | Δ14 | Δ24 | Δ32 | gRNA4, *cas9, hpt* |
| **>>>F4** | 97x113-4-3-6-10 | ab*d | Δ14 | Δ24 | Δ32 | gRNA4, *cas9, hpt* |
| **>>DH** | 97x113-4-3-DH4 | abd | Δ14 | Δ5/+31 | +1 | gRNA4, *cas9, hpt* |
|  | 97x113-4-3-DH5 | abd | +1 | Δ5/+31 | Δ2 | gRNA4, *cas9, hpt* |
|  | 97x113-4-3-DH8 | abd* | Δ1 | Δ5/+31 | Δ8/+2 | gRNA4, *cas9, hpt* |
| *97x113 primary crossing filial family 5* | | | | | | |
| **F1** | 97x113-5 |  | *wt* | *wt* | *wt* (het) | gRNA4 |
| **>F2** | 97x113-5-3 |  | *wt* | *wt* | Δ11/+1 |  |
| **>>F3** | 97x113-5-3-1 |  | *wt* | *wt* | Δ11/+1 |  |
| **>>>F4** | 97x113-5-3-1-1 |  | *wt* | *wt* | Δ11/+1 |  |
| **>>>>F5** | 97x113-5-3-1-1-1 | ABd | *wt* | *wt* | Δ11/+1 |  |
| **>F2** | 97x113-5-5 |  | *wt* | *wt* | Δ11/+1 |  |
| *C2/A (E111-4-2 x 97x113-5-5) back-crossing 2 filial family 3* | | | | | | |
| **BC-F1** | C2/A-3 |  | *wt* | *wt* | *wt* / Δ11/+1 |  |
| **>BC-F2** | C2/A-3-2 |  | Δ1 | *wt* | *wt* / Δ11/+1 |  |
| **>>BC-F3** | C2/A-3-2-8 | aBd | Δ1 | *wt* | Δ11/+1 |  |
| *C3/A (E101-1-1 x 97x113-5-3) back-crossing 3 filial family 1* | | | | | | |
| **BC-F1** | C3/A-1 |  | *wt* | *wt* / +1 | *wt* / Δ11/+1 |  |
| **>BC-F2** | C3/A-1-2 |  | *wt* | +1 | Δ11/+1 |  |
| **>>BC-F3** | C3/A-1-2-1 | Abd | *wt* | +1 | Δ11/+1 |  |
| *C4/A (E101-1-1 x E111-4-2) back-crossing 4 filial family 4* | | | | | | |
| **BC-F1** | C4/A-4 |  | *wt* / Δ1 | *wt* / +1 | *wt* |  |
| **>BC-F2** | C4/A-4-9 |  | Δ1 | *wt* / +1 | *wt* |  |
| **>>BC-F3** | C4/A-4-9-4 | abD | Δ1 | +1 | *wt* |  |
| *C6/A (Bobwhite x E6-3-8) back-crossing 6 filial family 1* | | | | | | |
| **BC-F1** | C6/A-1 |  | *wt* / Δ20 | *wt* / Δ6 | *wt* / Δ15 | gRNA4, gRNA5, *cas9, hpt* |
| **>BC-F2** | C6/A-1-15 |  | *wt* / Δ20 | Δ6 | *wt* |  |
| **>>BC-F3** | C6/A-1-15-13 | Ab*D | *wt* | Δ6 | *wt* |  |
|  | C6/A-1-15-15 | ab*D | Δ20 | Δ6 | *wt* |  |
| **>BC-F2** | C6/A-1-17 |  | Δ20 | *wt* | *wt* / Δ15 |  |
| **>>BC-F3** | C6/A-1-17-16 | aBd* | Δ20 | *wt* | Δ15 |  |
| **>BC-F2** | C6/A-1-20 |  | Δ20 | *wt* / Δ6 | *wt* / Δ15 |  |
| **>>BC-F3** | C6/A-1-20-5 | aBD | Δ20 | *wt* | *wt* |  |

**Table S10** Ploidy and fertility of haploids generated via anther culture

| **Plant-ID** | **Number of donor spikes for anther excision** | **Number of regenerant~~e~~s (fertile/sterile/dead)** | | | | **Doubled haploid mutant plants** |
| --- | --- | --- | --- | --- | --- | --- |
|  |  | ***total*** | ***allohexaploid*** | ***trihaploid*** | ***nonaploid*** |  |
| E6-3-2 | 5 | 2 | 2 *(2/0/0)* |  |  |  |
| E6-3-4 | 3 | 1 |  |  | 1 *(0/1/0)* |  |
| E101-2-2 | 4 | 9 | 9 *(3/6/0)* |  |  |  |
| E111-1-7 | 4 | 13 | 5 *(1/2/2)* | 8 *(0/3/5)* |  |  |
| E111-1-8 | 7 | - |  |  |  |  |
| E111-4-3 | 5 | 1 |  | 1 *(0/0/1)* |  |  |
| E111-4-7 | 5 | 17 | 14 *(13/1/0)* | 3 *(0/0/3)* |  |  |
| E111-4-8 | 5 | 14 | 5 *(4/1/0)* | 9 *(1/2/6)* |  | 2 |
| E113-3-1 | 8 | 11 | 2 *(2/0/0)* | 9 *(1/3/5)* |  |  |
| E113-3-5 | 5 | 1 |  | 1 *(1/0/0)* |  |  |
| E113-4-3 | 3 | - |  |  |  |  |
| 97x113-3-1 | 4 | - |  |  |  |  |
| 97x113-3-7 | 6 | - |  |  |  |  |
| 97x113-4-3 | 7 | 10 | 5 *(2/3/0)* | 5 *(0/1/4)* |  | 2 |
| 97x113-4-8 | 5 | 3 | 2 *(2/0/0)* | 1 *(0/0/1)* |  |  |

**Table S11** Overview of heritable WFZP alleles generated; * in-frame mutation; green: WFZP-target motif 4 with underlined PAM; red: mutation; blue: microhomology

| ***WFZP*-homoeo-allele** | **Mutation** | **DNA sequence of target region (5'>3')** |
| --- | --- | --- |
| ***WFZP-*A** | Bobwhite | **ACGCGGCCGAGATACGCGACCCGACCACCAAGGAGCGGCACTGGCTCGGCACCTTCGACACGGCGCAG** |
|  | Δ1 bp | **ACGCGGCCGAGATACGCGACCCGACCACCAA-GAGCGGCACTGGCTCGGCACCTTCGACACGGCGCAG** |
|  | Δ20 bp | **ACGCGGCCGAGATACGCGACCCGA--------------------CTCGGCACCTTCGACACGGCGCAG** |
| ***WFZP-*B** | Bobwhite | **ACGCGGCCGAGATACGCGACCCGACCACCAAGGAGCGGCACTGGCTCGGCACCTTCGACACGGCGCAG** |
|  | +1 bp | **ACGCGGCCGAGATACGCGACCCGACCACCAAGGGAGCGGCACTGGCTCGGCACCTTCGACACGGCGCAG** |
|  | Δ6 bp* | **ACGCGGCCGAGATACGCGACCCGACC------GAGCGGCACTGGCTCGGCACCTTCGACACGGCGCAG** |
|  | Δ11 bp | **ACGCGGCCGAGATACGCGACCCGACCACCA-----------TGGCTCGGCACCTTCGACACGGCGCAG** |
|  | Δ36 bp* | **ACGCGGCCGAGATACGCGACCCGACCACCA------------------------------------AG** |
|  | Δ39 bp* | **AC---------------------------------------TGGCTCGGCACCTTCGACACGGCGCAG** |
|  | Δ10/+13bp* | **ACGCGGCCGAGATACGCGACCCGCGCACGTCCAGGAAGCGGCACTGGCTCGGCACCTTCGACACGGCGCAG** |
|  | Δ5/+31 bp | **ACGCGGCCGAGATACGCGACCCGACCACCTTCGACACGGCCTACGACCACCTCGTGGCAGGCGGCACTGGCTC** |
| ***WFZP-*D** | Bobwhite | **ACGCGGCGGAGATACGCGACCCGACCACCAAGGAGCGGCACTGGCTCGGCACCTTCGACACGGCGCAG** |
|  | Δ1 bp | **ACGCGGCGGAGATACGCGACCCGACCACCAA-GAGCGGCACTGGCTCGGCACCTTCGACACGGCGCAG** |
|  | Δ15 bp* | **ACGCGGCGGAGATACGCGA---------------GCGGCACTGGCTCGGCACCTTCGACACGGCGCAG** |
|  | Δ18/+7 bp | **ACGCGGCGGAGATACGCG-----GCCGTGG------GGCACTGGCTCGGCACCTTCGACACGGCGCAG** |
|  | Δ11/+1 bp | **ACGCGGCGGAGATACGCGACC----A------GAGCGGCACTGGCTCGGCACCTTCGACACGGCGCAG** |
|  | Δ8/+2 bp* | **ACGCGGCGGAGATACGCGACCCGACC---CG---GCGGCACTGGCTCGGCACCTTCGACACGGCGCAG** |

**Table S12** Overview of gene products of mutated WFZP alleles; blue: AP2 domain; red: altered amino acid sequence; /: deleted amino acids

| **Modified WFZP proteins** | **Amino acid sequence** |
| --- | --- |
| **WFZP-A** | MSSRSSSGGGGASQMMAFSEHSLPKPIAGHPQPQPSPPSSPSERPAARGRRRAQEPGRFLGV  RRRPWGRYAAEIRDPTTKERHWLGTFDTAQEAALAYDRAALSMKGAQARTNFVYAHAAYNNY  PPFLAPFHAQHQPAAYAASSAMPYGGQQQHAGAGPPHIGSSYHHGHGYHQQGPGECSMPVPS  AADHGASGPMDVRGSSGHDFLFPSADDNSGYLSSVVPESCLRPRGGDLQDARRYSVSDADAY  GLGLREDVDDLATMVAGFWGGADAPYGGGHDMVASSQGSDNGYSPFSFLSH |
| Δ1 bp | MSSRSSSGGGGASQMMAFSEHSLPKPIAGHPQPQPSPPSSPSERPAARGRRRAQEPGRFLGV  RRRPWGRYAAEIRDPTTKSGTGSAPSTRRRRPPWPTTAPRSP |
| Δ20 bp | MSSRSSSGGGGASQMMAFSEHSLPKPIAGHPQPQPSPPSSPSERPAARGRRRAQEPGRFLGV  RRRPWGRYAAEIRDPTRHLRHGAGGRPGLRPRRALHEGRAGAHQLRLRARRLQQLPALPRAV  PRAAPARRLRRVLGHAVRRPAAARGRGAAAHWQLVPPRPRLPPAGPGRVFHAGAQCRGSRRQ  RPDGRARQQRPRLPLPQRRRQLRVPEQRGAGELPPAPRRRPAGRAALLRVRRRRLRAGPPGG  RGRPGDDGGRLLGRRRRAVRRRPRHGRLVAGLGQRLLPLQLPLPL |
| **WFZP-B** | MSSRSSSGGGQASQMMAFSEHSLPKPIAGHPQPQPSPPSSPSERPAPRGRRRAQEPGRFLGV  RRRPWGRYAAEIRDPTTKERHWLGTFDTAQEAALAYDRAALSMKGAQARTNFVYAHAAYNNY  PPFLAPFHAQQQPAAYASSTMPYGGQQRAGAAAPHIGSSYHGHGYHQQGPGAGAGECSMPVL  NAADHGASSPMDVRGSGGHDILFPSADDNSGYLSSVVPESCLRPRGGDLQDARRYSVSDADA  YGLGLREDVDDLALMVAGFWGGADAAYGGFAPANGGGHDMVASSQGSDNGYSPFSFLSH |
| Δ10/ +13 bp | MSSRSSSGGGQASQMMAFSEHSLPKPIAGHPQPQPSPPSSPSERPAPRGRRRAQEPGRFLGV  RRRPWGRYAAEIRDPRTSRKRHWLGTFDTAQEAALAYDRAALSMKGAQARTNFVYAHAAYNN  YPPFLAPFHAQQQPAAYASSTMPYGGQQRAGAAAPHIGSSYHGHGYHQQGPGAGAGECSMPV  LNAADHGASSPMDVRGSGGHDILFPSADDNSGYLSSVVPESCLRPRGGDLQDARRYSVSDAD  AYGLGLREDVDDLALMVAGFWGGADAAYGGFAPANGGGHDMVASSQGSDNGYSPFSFLSH |
| Δ6 bp | MSSRSSSGGGQASQMMAFSEHSLPKPIAGHPQPQPSPPSSPSERPAPRGRRRAQEPGRFLGV  RRRPWGRYAAEIRDPT//ERHWLGTFDTAQEAALAYDRAALSMKGAQARTNFVYAHAAYNNY  PPFLAPFHAQQQPAAYASSTMPYGGQQRAGAAAPHIGSSYHGHGYHQQGPGAGAGECSMPVL  NAADHGASSPMDVRGSGGHDILFPSADDNSGYLSSVVPESCLRPRGGDLQDARRYSVSDADA  YGLGLREDVDDLALMVAGFWGGADAAYGGFAPANGGGHDMVASSQGSDNGYSPFSFLSH |
| Δ36 bp | MSSRSSSGGGQASQMMAFSEHSLPKPIAGHPQPQPSPPSSPSERPAPRGRRRAQEPGRFLGV  RRRPWGRYAAEIRDPTTK////////////EAALAYDRAALSMKGAQARTNFVYAHAAYNNY  PPFLAPFHAQQQPAAYASSTMPYGGQQRAGAAAPHIGSSYHGHGYHQQGPGAGAGECSMPVL  NAADHGASSPMDVRGSGGHDILFPSADDNSGYLSSVVPESCLRPRGGDLQDARRYSVSDADA  YGLGLREDVDDLALMVAGFWGGADAAYGGFAPANGGGHDMVASSQGSDNGYSPFSFLSH |
| Δ39 bp | MSSRSSSGGGQASQMMAFSEHSLPKPIAGHPQPQPSPPSSPSERPAPRGRRRAQEPGRFLGV  RRRPWGRY/////////////WLGTFDTAQEAALAYDRAALSMKGAQARTNFVYAHAAYNNY  PPFLAPFHAQQQPAAYASSTMPYGGQQRAGAAAPHIGSSYHGHGYHQQGPGAGAGECSMPVL  NAADHGASSPMDVRGSGGHDILFPSADDNSGYLSSVVPESCLRPRGGDLQDARRYSVSDADA  YGLGLREDVDDLALMVAGFWGGADAAYGGFAPANGGGHDMVASSQGSDNGYSPFSFLSH |
| +1 bp | MSSRSSSGGGQASQMMAFSEHSLPKPIAGHPQPQPSPPSSPSERPAPRGRRRAQEPGRFLGV  RRRPWGRYAAEIRDPTTKGAALARHLRHGAGGRPGLRPCRALHEGRAGAHQLRLRARRLQQL  PALPRAVPRAAAARRLRVLHHAVRRATARGRGGAAHWQLVPRPRLPPAGPGRRRGRVLHAGA  QCRGSRRQQPDGRARQRRPRHPLPQRRRQLRVPEQRGAGELPPAPRRRPAGRAALLRVRRRR  LRAGPPGGRGRPGVDGGRLLGRRRRGVRRVRPRERRRPRHGRLVAGLRQRLLPLQLPLTL |
| Δ11 bp | MSSRSSSGGGQASQMMAFSEHSLPKPIAGHPQPQPSPPSSPSERPAPRGRRRAQEPGRFLGV  RRRPWGRYAAEIRDPTTMARHLRHGAGGRPGLRPCRALHEGRAGAHQLRLRARRLQQLPALP  RAVPRAAAARRLRVLHHAVRRATARGRGGAAHWQLVPRPRLPPAGPGRRRGRVLHAGAQCRG  SRRQQPDGRARQRRPRHPLPQRRRQLRVPEQRGAGELPPAPRRRPAGRAALLRVRRRRLRAG  PPGGRGRPGVDGGRLLGRRRRGVRRVRPRERRRPRHGRLVAGLRQRLLPLQLPLTL |
| Δ5/ +31 bp | MSSRSSSGGGQASQMMAFSEHSLPKPIAGHPQPQPSPPSSPSERPAPRGRRRAQEPGRFLGV  RRRPWGRYAAEIRDPTTFDTAYDHLVAGGTGSAPSTRRRRPPWPTTVPRSP |
| **WFZP-D** | MSIRSSSGGSGGGHASQMMAFSEHSLPKPIAGHPQPQPSPPSSPSERPAPRGRRRAQEPGRFLGVRRRPWGRYAAEIRDPTTKERHWLGTFDTAQEAALAYDRAALSMKGAQARTNFVYAHAAYNNYPPFLAPFHAQQQPAAYASSTMPYAGQQHAAPHIGSSYHHGHGHGGLGYHQQGPGAGAGECSMPVPNAADHGASSPMDVRGSSGHDFLFPSADDNSGYLSSVVPESCLRPRGGDLQDARRYSVSDADAYGLGLREDVDDLASMVAGFWGGADAAYGGFAPANGGGHDMVASSQGSDNGYSPFSFLSH |
| Δ8/ +2 bp | MSIRSSSGGSGGGHASQMMAFSEHSLPKPIAGHPQPQPSPPSSPSERPAPRGRRRAQEPGRFLGVRRRPWGRYAAEIRDPT/R/RHWLGTFDTAQEAALAYDRAALSMKGAQARTNFVYAHAAYNNYPPFLAPFHAQQQPAAYASSTMPYAGQQHAAPHIGSSYHHGHGHGGLGYHQQGPGAGAGECSMPVPNAADHGASSPMDVRGSSGHDFLFPSADDNSGYLSSVVPESCLRPRGGDLQDARRYSVSDADAYGLGLREDVDDLASMVAGFWGGADAAYGGFAPANGGGHDMVASSQGSDNGYSPFSFLSH |
| Δ15 bp | MSIRSSSGGSGGGHASQMMAFSEHSLPKPIAGHPQPQPSPPSSPSERPAPRGRRRAQEPGRFLGVRRRPWGRYAAEIR/////ERHWLGTFDTAQEAALAYDRAALSMKGAQARTNFVYAHAAYNNYPPFLAPFHAQQQPAAYASSTMPYAGQQHAAPHIGSSYHHGHGHGGLGYHQQGPGAGAGECSMPVPNAADHGASSPMDVRGSSGHDFLFPSADDNSGYLSSVVPESCLRPRGGDLQDARRYSVSDADAYGLGLREDVDDLASMVAGFWGGADAAYGGFAPANGGGHDMVASSQGSDNGYSPFSFLSH |
| Δ1 bp | MSIRSSSGGSGGGHASQMMAFSEHSLPKPIAGHPQPQPSPPSSPSERPAPRGRRRAQEPGRFLGVRRRPWGRYAAEIRDPTTKSGTGSAPSTRRRRPPWPTTAPRSP |
| Δ11/ +1 bp | MSIRSSSGGSGGGHASQMMAFSEHSLPKPIAGHPQPQPSPPSSPSERPAPRGRRRAQEPGRFLGVRRRPWGRYAAEIRDQSGTGSAPSTRRRRPPWPTTAPRSP |
| Δ18/ +7 bp | MSIRSSSGGSGGGHASQMMAFSEHSLPKPIAGHPQPQPSPPSSPSERPAPRGRRRAQEPGRFLGVRRRPWGRYAAEIRGRGALARHLRHGAGGRPGLRPRRALHEGRAGAHQLRLRARRLQQLPALPRAVPRAAAARRLRVLDHAVRRPAARGAAHWQLVPPRPRPRRPRLPPAGPGRRRGRVLHAGAQCRRSRRQQPDGRARQQRPRLPLPQRRRQLRVPEQRGAGELPPAPRRRPAGRAALLRVRRRRLRAGPPGGRGRPGVHGGRLLGRRRRGVRRVRPRERRRPRHGRLVAGLRQRLLPLQLPLPL |

**Table S13** Overview of non-inherited WFZP alleles generated; * in-frame mutation; green: WFZP-target motifs 4 or 5 with underlined PAM; red: mutation; blue: microhomology

| **TM and *WFZP*-homoeoallele** | **Mutation** | **DNA sequence of target region (5'>3')** |
| --- | --- | --- |
| **TM4/ *WFZP*-A** | Bobwhite | **ACGCGGCCGAGATACGCGACCCGACCACCAAGGAGCGGCACTGGCTCGGCACCTTCGACA** |
|  | +1 bp | **ACGCGGCCGAGATACGCGACCCGACCACCAAAGGAGCGGCACTGGCTCGGCACCTTCGACA** |
|  | Δ14 bp | **ACGCGGCCGAGATACGCGACCCG--------------GCACTGGCTCGGCACCTTCGACA** |
| **TM4/ *WFZP*-B** | Bobwhite | **ACGCGGCCGAGATACGCGACCCGACCACCAAGGAGCGGCACTGGCTCGGCACCTTCGACA** |
|  | Δ10 bp | **ACGCGGCCGAGATACGCGACCC----------GAGCGGCACTGGCTCGGCACCTTCGACA** |
|  | Δ24 bp* | **ACGCGGCCGAGATACGCGACCCGAC------------------------CACCTTCGACA** |
| **TM4/ *WFZP*-D** | Bobwhite | **ACGCGGCGGAGATACGCGACCCGACCACCAAGGAGCGGCACTGGCTCGGCACCTTCGACA** |
|  | Δ2 bp | **ACGCGGCGGAGATACGCGACCCGACCACCA--GAGCGGCACTGGCTCGGCACCTTCGACA** |
|  | Δ16 bp | **ACGCGGCGGAGATACGCGACCCGACC----------------GGCTCGGCACCTTCGACA** |
|  | Δ32 bp | **ACGCGGC--------------------------------ACTGGCTCGGCACCTTCGACA** |
| **TM5/ *WFZP*-A** | Bobwhite | **CGCGCACCAACTTCGTCTACGCGCACGCCGCCTACAACAACTACCCGCCCTTCCTCGCGC** |
|  | Δ1 bp | **CGCGCACCAACTTCGTCTACGCGCACGCCGCC-ACAACAACTACCCGCCCTTCCTCGCGC** |
|  | Δ7 bp | **CGCGCACCAACTTCGTCTACGCGCACGCCG-------CAACTACCCGCCCTTCCTCGCGC** |
| **TM5/ *WFZP*-B** | Bobwhite | **CGCGCACCAACTTCGTCTACGCGCACGCCGCCTACAACAACTACCCGCCCTTCCTCGCGC** |
|  | Δ22 bp | **CGCGCACCAACTTCGTCTACGCGCAC----------------------CCTTCCTCGCGC** |
| **TM5/ *WFZP*-D** | Bobwhite | **CGCGCACCAACTTCGTCTACGCGCACGCCGCCTACAACAACTACCCGCCCTTCCTCGCGC** |
|  | Δ3 bp* | **CGCGCACCAACTCCGTCTACGCGCACGCCG---ACAACAACTACCCGCCCTTCCTCGCGC** |
|  | Δ58/+7 bp* | **C------------------------CGCCCCA---------------------------C** |
|  | Δ64 bp | **…---------------------------------CAACAACTACCCGCCCTTCCTCGCGC** |
|  | Δ9/+6 bp* | **CGCGCACCAACTCCGTCTACGCGC--GCGCCG-ACAACAACTACCCGCCCTTCCTCGCGC** |

**Table S14** Grain number per spike from WFZP mutants growing under greenhouse conditions; * in-frame mutation

|  | **WT** | **aBd** | **ab*d** | **abd** | **abd*** | **ab*d* bΔ6bp** | **ab*d* bΔ36bp** |
| --- | --- | --- | --- | --- | --- | --- | --- |
| Plant IDs | **E108-1-1** | **C2/A-3-2-8**  **E111-4-8-DH3** | **97x113-4-3-6-6-9**  **97x113-4-3-6 -4/-5/-7/-10** | **97x113-4-3-DH4/DH5**  **97x113-4-3-2** | **97x113-4-3-DH8** | **E6-3-2-2-1-1** | **E6-3-4-5-10-1** |
| n (plants) | 6 | 7 | 5 | 3 | 5 | 4 | 5 |
| **Grain number per spike** | 64 | 45 | 0 | 0 | 0 | 2 | 3 |
|  | 39 | 45 | 0 | 0 | 1 | 12 | 1 |
|  | 22 | 14 | 0 | 0 | 0 | 0 | 1 |
|  | 53 | 25 | 0 | 0 | 1 | 6 | 0 |
|  | 24 | 45 | 0 | 0 | 1 | 0 | 2 |
|  | 67 | 27 | 0 | 0 | 0 | 2 | 2 |
|  | 26 | 44 | 0 | 0 | 0 | 1 | 0 |
|  | 52 | 45 | 0 | 0 | 0 | 4 | 1 |
|  | 19 | 6 | 0 | 0 | 0 | 3 | 0 |
|  | 14 | 16 | 0 | 0 | 0 | 0 | 5 |
|  | 16 | 38 | 0 | 0 | 0 | 1 | 1 |
|  | 44 | 21 | 0 | 0 | 0 | 7 | 2 |
|  | 43 | 46 | 0 | 0 | 0 | 8 | 2 |
|  | 24 | 19 | 0 |  | 0 | 0 | 1 |
|  | 28 | 23 | 0 |  | 0 | 0 | 0 |
|  | 18 | 33 | 0 |  | 1 | 0 | 2 |
|  | 16 | 33 | 0 |  | 0 | 7 | 1 |
|  | 32 | 34 | 0 |  | 0 | 3 | 2 |
|  | 57 | 14 | 0 |  | 0 | 2 | 6 |
|  | 53 | 46 | 0 |  | 1 | 0 | 0 |
|  | 15 | 37 | 0 |  | 0 | 4 | 1 |
|  | 51 | 38 | 0 |  | 0 | 2 | 0 |
|  | 16 | 35 | 0 |  | 0 | 0 | 6 |
|  | 20 | 23 | 0 |  | 0 | 6 | 5 |
|  | 33 | 53 | 0 |  | 0 | 12 | 0 |
|  | 57 | 12 | 0 |  |  | 0 |  |
|  | 64 | 32 | 0 |  |  | 9 |  |
|  | 44 | 17 | 0 |  |  | 11 |  |
|  | 34 | 15 | 0 |  |  | 0 |  |
|  | 17 | 17 | 0 |  |  |  |  |
|  | 11 |  | 0 |  |  |  |  |
|  | 63 |  | 0 |  |  |  |  |
|  | 71 |  | 0 |  |  |  |  |
|  | 41 |  | 0 |  |  |  |  |
|  | 69 |  | 0 |  |  |  |  |
|  | 29 |  | 0 |  |  |  |  |
|  |  |  | 0 |  |  |  |  |
| ***Median*** | **33,5** | **32,5** | **0,0** | **0,0** | **0,0** | **2,0** | **1,0** |
| ***Standard deviation*** | **18,8** | **13,0** | **0,0** | **0,0** | **0,4** | **3,9** | **1,9** |

**Table S15** Phenotyping of WFZP mutants growing under field-like conditions; grey: outliers with individual MAD >2,5xMAD of all values; * in-frame mutation.

(see extra file)

**Table S16** MARVIN grain analyses of WFZP mutants grown under field-like conditions; grey: outliers with individual MAD >2,5xMAD of all values; * in-frame mutation.

(see extra file)

**Table S17** Grain yield from WFZP mutants grown under greenhouse conditions; grey: outliers with individual MAD >2,5xMAD of all values; * in-frame mutation.

(see extra file)

**Table S18** Automatic root phenotyping of WFZP mutants - saRIA-data; grey: outliers with individual MAD >2,5xMAD of all values.

(see extra file)

**Table S19** Automatic shoot phenotyping of WFZP mutants - fresh and dry mass; grey: outliers with individual median absolute deviation (MAD) >2,5xMAD of all values

| **Genotype** | **Plant ID** | **Fresh weight [g]** | **Dry weight [g]** |
| --- | --- | --- | --- |
| E108 WT BW/Fl | 2010CH1171 | 18,7 | 2,6 |
|  | 2010CH1204 | 17,6 | 2,7 |
|  | 2010CH1286 | 19,8 | 3,1 |
|  | 2010CH1270 | 18,3 | 2,8 |
|  | 2010CH1142 | 19,3 | 3,4 |
|  | 2010CH1125 | 29,4 | 4,9 |
|  | 2010CH1221 | 32,9 | 5,2 |
|  | *Median* | 18,7 | 2,8 |
|  | *Standard deviation* | 0,9 | 0,3 |
| E111-4-2-1 *WFZP*-A-KO | 2010CH1199 | 15,1 | 2,3 |
|  | 2010CH1099 | 19,2 | 3,0 |
|  | 2010CH1303 | 23,3 | 3,8 |
|  | 2010CH1333 | 24,6 | 4,1 |
|  | 2010CH1089 | 21,3 | 3,7 |
|  | 2010CH1313 | 24,1 | 4,1 |
|  | 2010CH1242 | 25,5 | 3,9 |
|  | *Median* | 23,7 | 3,9 |
|  | *Standard deviation* | 2,4 | 0,2 |
| E101-1-1-1 *WFZP*-B-KO | 2010CH1134 | 11,2 | 1,5 |
|  | 2010CH1067 | 11,4 | 1,8 |
|  | 2010CH1394 | 13,2 | 2,1 |
|  | 2010CH1157 | 25,6 | 3,8 |
|  | 2010CH1023 | 20,0 | 3,1 |
|  | 2010CH1090 | 21,8 | 3,5 |
|  | 2010CH1058 | 18,4 | 3,1 |
|  | *Median* | 18,4 | 3,1 |
|  | *Standard deviation* | 5,6 | 0,9 |

**Table S20** Automatic shoot phenotyping of WFZP mutants- IAP-data; grey: outliers with individual MAD >2,5xMAD of all values; analyzed parameters.

(see extra file)

**Table S21** Predicted structures of modified COM2 and WFZP proteins; predicted protein structures are indicated and colored by AlphaFolds confidence with the corresponding multiple sequence alignment and the predicted aligned error.

(see extra file)

**Table S22** GO enrichment analysis of the rtt1 DEGs.

(see extra file)

**Table S23** Media composition for ballistic wheat transformation and production of wheat doubled haploids

| **Medium** | **Composition** |
| --- | --- |
| *Ballistic plant transformation and regeneration* | |
| Callus formation medium (CFM) | 4.4 g/L MS medium 36 g/L maltose 0.5 g/L glutamine 0.1 g/L casein hydrolysate 0.5 mL/L 10 mM CuSO_4_ 10 mL/L 2,4-D (2 mg/L) optional: 30 g/L (pre-culture) or 5 g/L (callus induction) centrophenoxine 20 or 50 mg/L hygromycin pH 5.8 3.5 g/L phytagel |
| CFM-Mannitol | 4.4 g/L MS medium 36 g/L maltose 63.75 g/L mannitol (0.35 M) 0.5 g/L glutamine 0.1 g/L casein hydrolysate 0.5 mL/L 10 mM CuSO_4_ 10 mL/L 2,4-D (2 mg/L) pH 5.8 3.5 g/L phytagel |
| K4N macro salts (20x) | 1.6 g/L NH_4_NO_3_ 40.4 g/L KNO_3_ 6.8 g/L KH_2_PO_4_ 8.82 g/L CaCl_2_x2H_2_O 4.92 g/L MgSO_4_x7H_2_O |
| K4N micro salts (1000x) | 1.12 g/100 mL MnSO_4_x4H_2_O 0.31 g/100 mL H_3_BO_3_ 0.72 g/100 mL ZnSO_4_x7H_2_O 12 mg/100 mL Na_2_MoO_4_x2H_2_O 2.5 mg/100 mL CuSO_4_x5H_2_O 2.4 mg/100 mL CoCl_2_x6H_2_O 16 mg/100 mL KI |
| Shoot formation medium | 50 mL/L K4N macro salts (20x) 1 mL/L K4N micro salts (1000x) 100 mL/L 1 M maltose 1 mL/L 75 mM NaFeEDTA 1 mL/L B5 vitamins (1000x, 112 g/L) 4 mL/L 0.25 M L-glutamine 490 µL/L 10 mM CuSO_4_x5H_2_O 1 mL/L 1 mM 6-BAP 25 mg/L Hygromycin (not for DH production) pH 5.8 3,5 g/L phytagel |
| Root regeneration medium | 33.34 mL/L K4N macro salts (20x) 670 µL/L K4N micro salts (1000x) 66.67 mL/L 1 M maltose 670 µL/L 75 mM NaFeEDTA 670 µL/L B5 vitamins (1000x, 112 g/L) 2.67 mL/L 0.25 M L-glutamine 330 µL/L 10 mM CuSO_4_x5H_2_O 20 mg/L Hygromycin pH 5.8 3,5 g/L phytagel |
| *Anther culture* |  |
| Wheat anther culture medium (WAM) macro elements (20x) | 23 g/L KNO_3_ 0.7 g/L KCl 2 g/L (NH_4_)_2_SO_4_ 4 g/L KH_2_PO_4_ 2 g/L Ca(NO_3_)_2_x4H_2_O 2.5 g/L MgSO_4_x7H_2_O |
| WAM iron source (500x) | 18.65 g/L Na_2_EDTA 13.9 g/L FeSO_4_x7H_2_O |
| WAM | 50 mL/L WAM macro elements (20x) 2 mL/L WAM iron source (500x) 7.5 mL/L 2,4-D (20 mg/100 mL) 1 mL/L kinetin (1000x, 50 mg/100 mL) 80 g/L maltose 100 g/L Ficoll Sigma PM400 optional: 4 mL/L K4N micro salts (1000x) pH 5.8 1 mL/L thiamine HCl (1 g/L) |

1. *Supporting experimental procedures*

**Methods S1** Whole-genome shotgun sequencing

For whole-genome shotgun sequencing (WGS), the *RTT* wild-type and mutant individuals were identified based on the *HvFZP* CAPS marker screening in *RTT* segregation progeny. DNAs from five wild-type and mutant individuals (genotypically/phenotypically) were separately pooled and subjected to WGS. The genomic DNA library preparation and sequencing steps were outsourced to Novogene (Novogene Co., Ltd; Cambridge, UK). Genomic DNAs were sequenced using PE150 NovaSeq 6000 platform (Illumina), with an output of ≥100 Gb sequence data per sample (≈20X coverage). Paired-end reads were first aligned to the Morex reference genome version 2 (Monat et al., 2019) using bwa-mem (0.7.17; Li, 2013). Duplicated, unmapped and low quality (quality ≤ 20) reads were removed using SAMtools (v1.9; Li et al., 2009) (−view −F 4 −bq 20 and –rmdup). Then, variants were called with SAMtools/Bcftools mpileup (v1.9). SNP density in a 1-kb bin size was calculated using VCFtools (Danecek et al., 2011) and visualized with R. (Repository ID PRJEB85878).

**Methods S2** Transcriptome studies of barley rtt1

For RNAseq, the spike meristems at lemma primordium (W 3.0) were collected from the progeny of heterozygous *RTT1.b/rtt1.a* individuals. The spike meristems from genotypically wild-type and mutant individuals (identified based on FZP CAPS marker) from *rtt* population were sampled and pooled. Four biologically replicated samples were collected for each condition. Total RNA was prepared via TRIzol (Invitrogen)-based extraction and precipitated with 2-propanol (Poursarebani et al., 2020). Residual genomic DNA contamination was removed with TURBO DNA-free™ kit (Invitrogen).

The RNAseq library construction and sequencing were outsourced to Novogene (UK) Company Limited. The RNA integrity and quantity were determined using the Agilent 2100 Bioanalyzer. Briefly, the total mRNA was enriched with oligo(dT) beads and randomly fragmented, unstranded *mRNA* library was prepared for each sample. Size selected libraries were sequenced using PE150 NovaSeq 6000 platform (Illumina), with an output of up to 50 Mio high quality reads per sample. The RNAseq reads were initially trimmed-off the adaptor sequences using Trimmomatic v.0.39 (Bolger et al., 2014) using default criteria set in the Galaxy web suite. Read abundances for each sample were estimated by mapping trimmed sequence data against Morex.v3 reference using Kallisto (Bray et al., 2016). The differentially expressed genes (DEG) analysis was conducted in a pair-wise manner between *RTT1* wild-type and mutant samples using edgeR package implemented in BEST DEGs pipeline (Sangket et al., 2022). Threshold LOG2 fold change ≥0.5 and an false discovery rate (FDR) adjusted *p*-value of <0.05 were applied to declare the genes as differentially regulated. (Repository ID PRJEB85879)

All upregulated and downregulated DEGs were independently subjected to gene ontology (GO) enrichment analysis in Metascape with default settings (Zhou et al., 2019). Closest *Arabidopsis* orthologs of barley genes (BLAST e-value threshold <1.0×10^−5^) were used as background reference for GO enrichment. Significance of enrichment was estimated by q-value statistics (threshold ≤ 0.0001). DEG clustering and GO terms were visualized using the SRplot pipeline (Tang et al., 2023; Table S22).

For FZP binding motif search and motif enrichment analyses, 1700 bp upstream sequence from the start codon (promoter + 5’ UTR) of *RTT* DEGs (LOG2 FC ≥1.0; FDR: <0.05) was used. The motif finding tool FIMO was used to search for the known *FZP* GCC-box binding motif (SCCGCC) employing default parameters (*p*-value threshold <0.001). For motif enrichment analysis, the XSTREME tool from MEME suite (Bailey et al., 2015) was employed with default parameters. Significance of enrichment was estimated by Fisher exact test (E-value threshold <0.05).

**Methods S3** Construction of generic vector pNB38 and gRNA/cas9 vectors

To create the generic gRNA/*cas9* vector pNB38 (Figure **S6**; GenBank-ID OR479081), the synthesized (GenScript Biotech B.V., Leiden, The Netherlands) TaU6 promoter (TaU6p; Shan et al., 2013) and the generic vector pSH121 (GenBank-ID MW145140.1; Gerasimova et al., 2020) were digested with HindIII and purified. To prevent self-ligation, the pSH121 fragment was dephosphorylated. To form pNB38, TaU6p was ligated into pSH121 backbone and selection was performed by using Kanamycin. A test digestion with HindIII and Sanger sequencing using pSH91_S1 primer were performed to validate the vector.

The pNB38 vector was digested with BsaI and purified. Target motif-specific primers (TM4-F/-R or TM5-F/R; Table **S2**) were hybridized forming a double stranded DNA with 4 bp overhangs and subsequently ligated into pNB38, resulting in the intermediate vectors pCH26_WFZP-TM4-int and pCH27_WFZP-TM5-int. These vectors were Sanger sequenced using pSH91_S1 primer.

To form binary vectors, a fragment containing the expression cassettes of gRNA and *cas9* were excised using SfiI and subsequently ligated into binary vector p6i-d35S-TE9 (DNA Cloning Service, Hamburg, Germany) resulting in pCH28_WFZP-TM4 and pCH29_WFZP-TM5. Correct ligation was tested with restriction enzymes NotI and XhoI, and by Sanger sequencing with CH159-p6i-RB and CH160-p6i-E9 primers.

**Methods S4** Procedure of ballistic gene transfer to wheat and plant regeneration

Immature wheat caryopses harvested and collected on ice were surface-sterilized with 2.4% sodium hypochlorite (NaClO) and then rinsed in sterile water. The wheat transformation was performed according to the protocol of Ismagul and colleagues (2014) with some modifications (Table **S23**). The embryos were excised, positioned with the scutellum up on dark medium and cultured for five days at 24 °C in the dark. Half of the embryos were cultured on callus formation medium (CFM) containing centrophenoxine. In preparation for ballistic DNA transfer, the embryos were exposed to CFM-Mannitol for 4-6 hours at 24°C in the absence of light to reduce the cell turgor. 50 embryos were placed on each plate.

For coating, 25 µL gold (particle size 0.6, about 1500 µg) from glycerol stock solution containing
60 mg/mL gold was mixed with 25 µL 50% glycerol, 2 µg each of binary vectors pCH28_WFZP-TM4 and pCH29_WFZP-TM5, 20 µL spermidine and 50 µL calcium chlorite and then mixed for four minutes. Gold particles were sedimented, washed with 70% ethanol and resuspended in 60 µL of pure ethanol. To each plate of embryos, we prepared 5 µL of suspension containing 125 µg of gold and 0.33 µg of DNA for one shot. Ballistic gene transfer was performed using a *PDS-1000/He* particle gun (BIO-RAD, Munich, Germany) with a vacuum of 27 inHG and a burst pressure of 1100 pound-force per square inch.

After ballistic DNA transfer, embryos were incubated overnight on CFM-Mannitol and then transferred to CFM for callus formation. The incubation lasted for 7 days without, 14 days with 20 mg/L, and 7 days with 50 mg/L hygromycin. Half of the embryos that formed callus were moved to CFM containing supplementary centrophenoxine. Thereafter, the callus was transferred to shoot formation medium containing 25 mg/L hygromycin and were grown for six weeks at 24 °C with 10 hours light per day while changing the medium every two weeks. Emerged shoots were transferred to a root formation medium with a hygromycin concentration of 20 mg/L. Plantlets were then transferred to Substrate 1 (Klasmann-Deilmann GmbH, Geeste, Germany).

**Methods S5** Practical hints about crosses and production of doubled haploids in wheat

Mother plants were emasculated at the green anther stage to prevent self-pollination and facilitate crossings. Subsequently, within three days, a pollen-shedding anther from the father plant was transferred to a fanned-out stigma and spikes were covered with plastic bags.

To generate doubled haploids, anther culture was executed. Spikes from selected plants were harvested at the late microspore stage along with the flag leaf, and incubated in water at 4 °C for a duration of two weeks. The leaf blades were removed and the spike surface was sterilised using 70% ethanol. Anthers from two spikes were then transferred to 2.5 mL of wheat anther medium (WAM) (Table **S23**) and incubated for three days at 32 °C in the dark. Following this, six freshly isolated Bobwhite pistils that had been longitudinally cut were added (Lippmann et al., 2015) followed by 2.5 mL of fresh WAM. The anthers were cultured for four to six weeks at 25 °C in the dark to form callus. Embryogenic callus was moved to shoot formation medium at a temperature of 22 °C with 10 hours of light until shoots and roots appeared. To avoid light-induced stress, the plates were covered with paper for the initial three days. After regeneration, the plants were transferred to Substrate 1 (Klasmann-Deilmann GmbH, Geeste, Germany).

The ploidy status of the regenerated plants was determined using the *CyFlow Ploidy Analyzer* (Sysmex Partec GmbH, Görlitz, Germany). Leaf pieces were ground in *SYSMEX CyStain UV Ploidy buffer* (Sysmex Partec GmbH) and the extract filtered using *Cell Tric 30 µm* (Sysmex Partec GmbH) according to the manufactorer’s instructions. To double the genome of trihaploid plants, colchicine treatment was employed (Otto et al., 2015).

**Methods S6** Wheat plant phenotyping

*Manual phenotyping of plant and spike parameters*

Plants and mature spikes were photographed with a *FinePix S100FS* digital camera (FUJIFILM). In order to capture detailed images, *Leica MZFLIII* (Leica Microsystems GmbH, Wetzlar, Germany) and *Stemi 2000-C* (Carl Zeiss AG, Oberkochen, Germany) stereomicroscopes as well the *Keyence VHX-5000* digital microscope (Keyence Deutschland GmbH, Neu-Isenburg, Germany) were utilized.

The characteristics of plant architecture including plant height, number of tillers and shoot dry mass, as well as spike development traits like number of spikes and sterile spikes, spike length, number of spikelets and grains per spike were obtained manually.

*Semi-automated phenotyping of grains*

Grain size, length and width as well as thousand kernel weight were analysed using the *MARVin Seed Analyzer* (MARViTECH GmbH, Wittenburg, Germany).

*Calculated parameters*

Following traits were calculated:

spikelets per 1 cm spike = spikelets per spike / spike length

grains per 1 cm spike = grains per spike / spike length

grains per spikelet = grains per spike / spikelets per spike

grain yield per spike = thousand kernel weight * grains per spike / 1000

grain yield per plant = grain yield per spike * number of spikes

*Automatic phenotyping of roots and shoots*

Plants were cultivated for 44 days under controlled conditions in specialized root phenotyping pots (Shi et al., 2023) that were designed for the LemnaTec Scanalyzer system (LemnaTec AG, Aachen, Germany) for medium and large plants, and contained Potground P soil (Klasman-Deilmann GmbH, Geeste, Germany). The LemnaTec Scanalyzer system (LemnaTec AG, Aachen, Germany) for large plants at IPK Leibniz Institute Gatersleben, Germany was used for this purpose (Junker et al., 2015). The temperature was maintained between 26 °C during the day and 20 °C during the night, with 10 hours of light supplied each day. Root and shoot growth phenotyping was performed on seven replicates of *WFZP*-A and -B mutants, and Bobwhite as wild-type control, following Narisetti and colleagues (2021) with documentation at 16 time points (near infrared light - NIR) for roots and 17 time points (visible light - VIS, RGB; fluorescence light - FLUO) for shoots. The imaging was performed 3, 6, 8 (only shoots), 10, 14, 15, 17, 20, 22, 24, 27, 29, 31, 34, 37, 42, 44 days after sowing (DAS). The final shoot material was harvested and the fresh and dried mass were measured. *Semi-automated Root Image Analysis* software (saRIA, Narisetti et al., 2019) was used for analysing root branching, length, volume and surface area (Table **S1**). The *Integrated Analysis Platform* software (IAP, Klukas et al., 2014) was employed to quantify both plant height and shoot surface (Table **S20**). (Raw images and image analysis results available at <https://doi.org/10.5447/ipk/2026/3>)

1. *Supporting animations*

**Movie S1** Molecular dynamics simulation of the protein-DNA complex in Bowman. Residues R68 and R72 are shown as stick representations to emphasize their interactions with DNA. (see extra file)

**Movie S2** Molecular dynamics simulation of the protein-DNA complex for *rattail 1.a*. Residues R68 and R72 are shown as stick representations to emphasize their interactions with DNA. (see extra file)
